# Simultaneous Downregulation of *MTHFR* and *COMT* in Switchgrass Affects Plant Performance and Induces Lesion-Mimic Cell Death

**DOI:** 10.3389/fpls.2017.00982

**Published:** 2017-06-20

**Authors:** Sijia Liu, Chunxiang Fu, Jiqing Gou, Liang Sun, David Huhman, Yunwei Zhang, Zeng-Yu Wang

**Affiliations:** ^1^Department of Grassland Science, China Agricultural University, National Energy R&D Center for BiomassBeijing, China; ^2^Forage Improvement Division, The Samuel Roberts Noble Foundation, ArdmoreOK, United States; ^3^Qingdao Institute of Bioenergy and Bioprocess Technology, Chinese Academy of SciencesQingdao, China; ^4^BioEnergy Science Center, Oak Ridge National Laboratory (DOE), Oak RidgeTN, United States; ^5^Computing Services, The Samuel Roberts Noble Foundation, ArdmoreOK, United States; ^6^Plant Biology Division, The Samuel Roberts Noble Foundation, ArdmoreOK, United States

**Keywords:** bioenergy crop, caffeic acid *O*-methyltransferase (COMT), lesion-mimic cell death, lignin, methylenetetrahydrofolate reductase (MTHFR), *Panicum virgatum*, switchgrass, transgenic plants

## Abstract

Switchgrass (*Panicum virgatum*) has been developed into a model lignocellulosic bioenergy crop. Downregulation of caffeic acid *O*-methyltransferase (COMT), a key enzyme in lignin biosynthesis, has been shown to alter lignification and increase biofuel yield in switchgrass. Methylenetetrahydrofolate reductase (MTHFR) mediates C1 metabolism and provides methyl units consumed by COMT. It was predicted that co-silencing of *MTHFR* and *COMT* would impact lignification even more than either of the single genes. However, our results showed that strong downregulation of *MTHFR* in a *COMT*-deficient background led to altered plant growth and development, but no significant change in lignin content or composition was found when compared with *COMT* plants. Another unexpected finding was that the double *MTHFR/COMT* downregulated plants showed a novel lesion-mimic leaf phenotype. Molecular analyses revealed that the lesion-mimic phenotype was caused by the synergistic effect of *MTHFR* and *COMT* genes, with *MTHFR* playing a predominant role. Microarray analysis showed significant induction of genes related to oxidative and defense responses. The results demonstrated the lack of additive effects of *MTHFR* and *COMT* on lignification. Furthermore, this research revealed an unexpected role of the two genes in the modulation of lesion-mimic cell death as well as their synergistic effects on agronomic performance.

## Introduction

As an essential cofactor, tetrahydrofolate (THF) mediates the transfer of one-carbon (C1) units in various methylated anabolic pathways, mainly those for lignin, alkaloids and betaines (Hanson et al., 2000). Methylenetetrahydrofolate reductase (MTHFR) catalyzes the reduction of 5, 10-methylene-THF to 5-methyl-THF, which is the most reduced C1 derivative (Roje et al., 1999; Hanson et al., 2000). The latter then provides a methyl group to generate methionine (Met) from homocysteine (Roje et al., 1999; Hanson et al., 2000). Subsequently, nearly 80% of Met is converted to *S*-adenosyl-L-methionine (SAM), a universal methyl donor that is consumed by various methylation reactions in plant primary and secondary metabolic pathways (Giovanelli et al., 1985; Hanson et al., 2000; Hung et al., 2013).

In plants, MTHFRs have been cloned and identified in Arabidopsis (*Arabidopsis thaliana*), maize (*Zea mays*) and tobacco (*Nicotiana tabacum*) (Roje et al., 1999; Hung et al., 2013). Downregulation of *NtMTHFR1* dramatically induced the expression of a nicotine *N*-demethylase gene *CYP82E4* and altered the alkaloid profile in transgenic tobacco (Hung et al., 2013). A study in maize has confirmed that the *brown midrib 2* (*bm2*) phenotype is caused by MTHFR mutation (Tang et al., 2014). The *bm2* mutant with reduced transcriptional level of *MTHFR* showed decreased lignin content and altered lignin composition (Tang et al., 2014).

Caffeic acid *O*-methyltransferase (COMT) is one of the key enzymes involved in lignin biosynthesis. The maize *brown midrib 3* (*bm3*) and sorghum *bmr12* phenotypes characterized by the reddish-brown coloration at the leaf midribs are both caused by *COMT* mutation (Vignols et al., 1995; Bout and Vermerris, 2003); the mutants showed a reduction in lignin content and a strong decrease in S-lignin units (Kuc et al., 1968; Muller et al., 1972; Barrière et al., 2007; Guillaumie et al., 2008; Palmer et al., 2008). Suppressing COMT activity in switchgrass resulted in 6.4–14.7% decrease of acetyl bromide (AcBr) lignin content and reduced S/G ratio (Fu et al., 2011). In addition to the function associated with lignin biosynthesis, COMT has also been reported as a defense-related protein due to its strong response during pathogen infection and fungal-elicitor treatment (Quentin et al., 2009; Funnell-Harris et al., 2010). The Arabidopsis *comt1* mutants were more susceptible to two necrotophic fungi, one biotrophic fungus and two bacterial pathogens, but showed higher resistance to the biotrophic oomycete *Hyaloperonospora arabidopsis*, possibly due to the overaccumulation of 5-hydroxyferuloyl malate (OH-FM) in the mutant (Quentin et al., 2009). These evidences suggested a role of COMT in host defense mechanisms.

Tetrahydrofolate-mediated C1 metabolism controls carbon flux from MTHFR to methyltransferases (e.g., COMT) through the intermediate compound SAM. Thus, perturbing *MTHFR* expression would putatively impair the production of SAM, further reduce the availability of substrates required by enzymes involved in lignin biosynthesis. In a recent study involving folylpolyglutamate synthetase (FPGS), Srivastava et al. (2015) reported that the reduced lignin in Arabidopsis *fpgs1* mutants might not only be due to reduced flux of methyl units to lignin precursors, but also a result of changes in the expression of genes related to lignin biosynthesis and cell wall remodeling (Srivastava et al., 2015). Disruption of MTHFR may have similar effects because FPGS is an enzyme that acts upstream of MTHFR in the C1 pathway.

Gene stacking approaches for co-silencing both *COMT* and a gene involved in C1 metabolism could develop into potential strategies for further lignin manipulation. In maize, each single *bm2* or *bm4* mutant caused an 11% reduction in Klason lignin content in the midribs, and this effect is additive (20%) in the *bm2*-*bm4* double mutant (Vermerris et al., 2010). The *bm4* phenotype was recently identified to be caused by *FPGS* mutation (Li et al., 2015). In the case of maize *bm2-bm3* (*MTHFR-COMT*) double mutant, only plant height was measured and no significant difference was found between the mutant and wild type (Vermerris et al., 2010). Growth defects were observed in triple mutants containing *bm2* and *bm4*. *Bm1-bm2-bm4* stopped growing at an early seedling stage, while the combination of *bm2-bm3-bm4* arrested growth even prior to seedling emergence (Vermerris et al., 2010).

Switchgrass (*Panicum virgatum*) is a dedicated lignocellulosic bioenergy crop due to its high biomass productivity and low input requirements (Schmer et al., 2008). Molecular tools have been applied to genetic improvement of switchgrass (Casler et al., 2011; Wang and Brummer, 2012; Nageswara-Rao et al., 2013; Li et al., 2014). We generated low lignin switchgrass by *COMT* downregulation and showed that the transgenic plants had improved sugar release and ethanol yield (Fu et al., 2011). Field studies further confirm that *COMT* downregulation in switchgrass can confer real-world improvements in biofuel yield without negative impact on biomass productivity, disease susceptibility, soil chemistry or carbon storage potential (Baxter et al., 2014, 2015, 2016; DeBruyn et al., 2016; Dumitrache et al., 2016; Li et al., 2016).

In this study, we target *MTHFR* and *COMT* to investigate the function of these two genes in lignification and their potential synergetic effects on morphology in switchgrass. Our results showed that strong downregulation of *MTHFR* in a *COMT*-deficient background led to reduced plant growth. However, lignin content of the *MTHFR*/*COMT*-RNAi plants was similar to that of the single *COMT* downregulated plant. To our surprise, a novel lesion-mimic leaf phenotype was observed. Molecular analyses revealed that the lesion-mimic phenotype was regulated by both *MTHFR* and *COMT*, with *MTHFR* playing a predominant role. Microarray analysis demonstrated significant induction of genes related to oxidative and defense responses. This research not only demonstrated the synergistic effects of *MTHFR* and *COMT* on plant performance, but also revealed an unexpected role of the two genes in the modulation of lesion-mimic cell death.

## Materials and Methods

### Plant Materials

Lowland switchgrass cultivar Alamo (2*n* = 4 × = 36) was used for all the experiments described in this study. Plants were grown in the greenhouse with 16-h light photoperiod (390 μ Em^-2^ S^-1^) and 26°C. Three biological replications of plants were re-potted by splitting the same number of tillers. The development of switchgrass was divided into five elongation (E1, E2, E3, E4, and E5) and three reproductive (R1, R2, and R3) stages according to Hardin et al. (2013).

When switchgrass plants reached R1 stage, dry matter biomass, plant height, tiller number, leaf blade length and width, leaf sheath length, internode length, internode diameter and number, and flowering time were measured. Internode three (I3) and the leaf and leaf sheath of I3 were used for measurement.

### Vector Construction and Plant Transformation

Full-length mRNA sequence of *MTHFR* was identified from the switchgrass EST database^1^. Two *MTHFR* sequences (Pavir.Ia00159 and Pavir.J04665.1) were cloned, these sequences showed 98.5% similarity at the amino acid level. A 506-bp of RNAi cDNA fragment was amplified by PCR with primers shown in Supplementary Table S9 and cloned into the pENTR/D-TOPO Cloning vector (Invitrogen, Chicago, IL, United States). The pENTR-*MTHFR* plasmid was ligated to pANIC8D gateway vector through the LR Gateway cloning reaction (Invitrogen). The binary construct was transformed into *Agrobacterium tumefaciens* strain AGL1. Embryogenic calli induced from immature inflorescence of wild-type switchgrass plants were used for transformation (Xi et al., 2009). The *COMT*-RNAi transgenic plants were developed as previously described (Fu et al., 2011). The double *MTHFR/COMT*-RNAi transgenics were generated by transforming the *MTHFR*-RNAi vector into embryogenic calli induced from immature inflorescence of *COMT*-RNAi transgenics.

### Molecular Identification of Transgenic Plants

Genomic DNA was isolated from young leaves of wild-type and transgenic plants using 2xCTAB method (Doyle, 1987). Positive transgenics were identified by PCR amplification with specific *bar* and *hph* primers (Supplementary Table S9). The expected sizes of PCR amplification product for *bar* and *hph* were 444- and 403-bp, respectively.

Total RNA was isolated from switchgrass young leaves by TRIzol reagent (Invitrogen). First-strand cDNA was synthesized from the purified total RNA using Superscript III Kit (Invitrogen) and used for quantitative RT-PCR (qRT-PCR). Primers used for qRT-PCR were listed in Supplementary Table S9. Calculation of cycle threshold, qRT-PCR condition and data normalization was performed as described by Fu et al. (2012). *PvUbiquitin1* transcripts (GenBank accession number: FL899020) were used as internal control.

### Determination of Lignin Content and Composition

Stem tissues were harvested at R1 stage. Lyophilized extractive-free cell wall residues (CWR) were obtained as described by Chen and Dixon (2007), and used for determination of lignin content and composition. Lignin content was quantified by the Acetyl bromide (AcBr) method (Hatfield et al., 1999). Lignin composition was determined by the thioacidolysis method (Lapierre et al., 1995). Lignin-derived monomers (H, S, and G units) were identified and quantified by gas chromatography mass spectrometry (GC/MS) using a Hewlett-Packard 5890 series II gas chromatograph with a 5971 series mass selective detector (Fu et al., 2011).

### Trypan Blue and Diaminobenzidine (DAB) Staining

Leaves with lesion-mimic-like phenotype were collected and placed in trypan blue solution (Cat # 93595, Sigma, St. Louis, MO, United States) for determination of cell death. Chlorophyll was removed by soaking the stained leaves in 95% ethanol for 1–2 days until the leaves are free of chlorophyll. Leafs 2–5 collected at R1 stage were stained with 1 mg/ml (pH 3.8) 3,3C-diaminobenzidine (DAB) for determination of H_2_O_2_ as described by Daudi and O’Brien (2012). ImageJ software was used to quantify the area of the lesions on the leaf surface.

### Microarray Analysis

High-quality total RNA from duplicate biological replicates of the transgenic lines MT/CO-37 and MT/CO-47 (severe phenotype group), MT/CO-38 and MT/CO-67 (moderate phenotype group) was isolated from young leaves (Leaf 4) at E4 stage using Spectrum^TM^ Plant Total RNA Kit (Sigma–Aldrich). To eliminate background differences among transgenics, except wild-type plant, MT/CO-60 with no more than 40% decrease in expression of both *MTHFR* and *COMT* genes was also selected as a control. Leaf 4 at E4 stage was used because the lesion phenotype fully emerged at this stage in leaf 2, moderately developed in leaf 3, but could not be observed in leaf 4. RNA amplification, labeling and hybridization were done as described by Fu et al. (2012). Data normalization was performed by using the robust multi-array average (RMA) (Irizarry et al., 2003). Differentially expressed genes were selected based on associative *t*-test (Dozmorov and Centola, 2003) using Matlab (MathWorks, Natick, MA, United States). By using the method described by Dozmorov and Centola (2003), residual for each gene between sample groups was compared against residual of a group of background stable genes to obtain significant *P*-values. Bonferroni corrected threshold was applied to this *P*-value in order to remove false positives. Common differentially expressed genes were analyzed by venn diagram software^2^. Functional enrichments were conducted by PageMan (Usadel et al., 2006). Significance of pathway analysis was conducted by MAPMAN (Thimm et al., 2004). Nine upregulated genes were selected for qRT-PCR verification. Primers used for qRT-PCR were listed in Supplementary Table S9.

### Metabolite Analysis and Quantification of Amino Acids

Primary metabolites were analyzed using gas chromatography mass spectrometry (GC-MS). Leaf tissues (leafs 2 and 4) at E4 stage were collected and lyophilized. Sample grinding and extraction were achieved by a Labman Automation Robot (Labman Automation, Inc.). Totally, 10 mg of pulverized tissue was used for polar and non-polar extractions; the derivatization of extracts and GC-MS analysis followed the descriptions by Broeckling et al. (2005). Mass spectra deconvolution and metabolite identification were carried out using AMDIS software^3^ and a custom in-house EI-MS metabolite library. Peak picking, alignment and quantification were conducted using MET-IDEA software (Broeckling et al., 2006).

For quantification of amino acids, derivatization of polar extracts was done as described above. Polar samples were also analyzed by gas chromatography with Time-of-Flight mass spectrometer (GC-TOF-MS). Data format conversion was performed by R software^4^ and peak area of each amino acid was calculated by MET-IDEA software as mentioned. Authentic amino acid mixture (Sigma) was used as reference for quantitative analyses.

### Statistical Analysis

Data were analyzed using means ± SE of triplicate samples. Data from each trait were subjected to one-way ANOVA. Duncan’s multiple range test (*P* < 0.05 or 0.01) was applied to analyze the significant differences among means using SPSS software.

## Results

### Generation, Molecular Identification and Categorization of Transgenic Plants

To generate single *MTHFR* knockdown transgenic plants, a 506-bp RNAi cDNA fragment was designed at the conserved region of switchgrass *MTHFR* to downregulate the two homologous alleles after multiple alignment analysis (Supplementary Figure S1). An antisense and a sense *MTHFR* cDNA fragment were connected by a gus-linker to generate the hairpin RNA (Supplementary Figure S2a). Resistant embryogenic calli harboring the *MTHFR* RNAi vector were obtained after *Agrobacterium*-mediated transformation and phosphinothricin (PPT) selection. Fifteen positive transgenic *MTHFR*-RNAi plants were generated. qRT-PCR was performed to detect *MTHFR* expression levels (Supplementary Figure S3a). Out of the 15 transgenic events, two lines (MT-20 and MT-15) with approximately 50% decrease of *MTHFR* expression were selected for further analysis (Supplementary Figure S3a). It should be noted that compared to other gene constructs, it was surprisingly difficult and much more effort was needed to generate transgenic plants using the *MTHFR*-RNAi construct.

A *COMT*-RNAi line with more than 90% downregulation of *COMT* expression was selected as the background plant for *MTHFR*-RNAi vector transformation. Resistant calli of double *MTHFR/COMT*-RNAi plants were obtained after hygromycin and PPT selection. More than forty double *MTHFR/COMT* knockdown transgenic events were obtained. Positive events were detected by the presence of both *hph* and *bar* genes using PCR (Supplementary Figure S2b). Unlike single *MTHFR* knockdown, *MTHFR* in *COMT*-deficient background was easily downregulated. Some lines (MT/CO-37, 46, 47) showed more than 90% decrease in both *MTHFR* and *COMT* expression (Supplementary Figure S3b).

**FIGURE 1:**
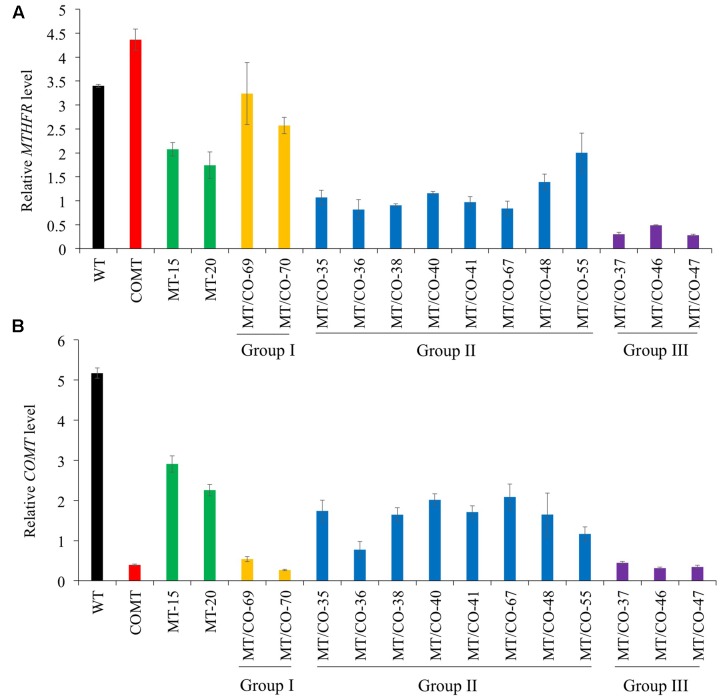
Molecular identification and categorization of transgenic switchgrass plant. Relative transcript levels of *MTHFR* **(A)** and *COMT* **(B)** in wild-type (WT) and transgenic plants detected by quantitative real-time PCR (qRT-PCR). Switchgrass *Ubiquitin 1* was used as the reference gene.

The expression profiles of selected transgenic plants were further confirmed by three independent qRT-PCR experiments, and consistent results were obtained (**Figures 1A,B**). We categorized the double gene knockdown transgenic switchgrass plants into three groups based on *MTHFR* and *COMT* expression levels. Group I plants were heavily downregulated in *COMT* but the *MTHFR* expression level was similar to wild type. Group II plants showed moderate downregulation of both *MTHFR* and *COMT*. Group III plants exhibited severe downregulation of both *MTHFR* and *COMT* (**Figures 1A,B**).

### Effects of *MTHFR* and *COMT* Downregulation on Plant Growth and Development

**FIGURE 2:**
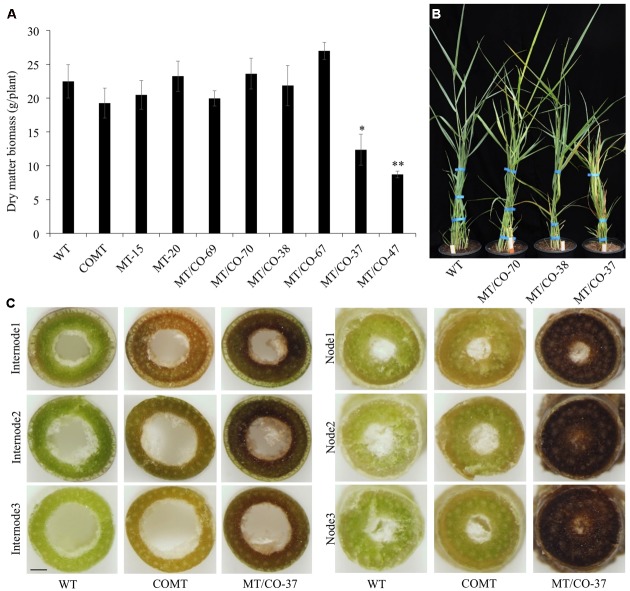
Dry matter biomass, plant growth and cross sections of WT and transgenic plants at Rl stage. **(A)** Dry matter biomass at Rl stage. One or two asterisks indicate significant difference of *P* < 0.05 or *P* < 0.01 by one way ANOVA. **(B)** Morphological observation of WT and representative transgenic plants (MTV CO-70 from group I, MT/CO-38 from group II, MT/CO-37 from group III). **(C)** Coloration of the cross sections of internode and node (without staining). Bar, 1 mm.

To evaluate whether downregulation of single *MTHFR* or double *MTHFR/COMT* affects switchgrass growth and development, transgenic lines were analyzed regarding their morphological characteristics. Single *MTHFR* knockdown lines MT-15 and MT-20 displayed normal morphology with no obvious changes in plant height, leaf width, leaf sheath length, internode length, internode diameter, or internode number (Supplementary Table S1), nor were significant changes observed in tiller number or biomass yield (Supplementary Figure S4 and **Figure 2A**). The only change observed in MT-15 and MT-20 was delayed flowering time (Supplementary Table S1). In double *MTHFR/COMT* knockdown plants, morphological traits, tiller number and dry matter biomass were not affected in group I (MT/CO-69 and MT/CO-70) and group II (MT/CO-38 and MT/CO-67) plants (**Figures 2A,B**, Supplementary Figure S4 and Table S1). The group III transgenic lines (MT/CO-37 and MT/CO-47), however, displayed severe defects in plant growth and development. They showed 33–46% reduction in plant height, 16–34% reduction in leaf blade length, 15–27% reduction in leaf blade width, 16–31% reduction in leaf sheath length (Supplementary Table S1). Even though tiller number was not affected (Supplementary Figure S4), the strong growth defects of group III lines led to 45–61% reduction in biomass yield (**Figure 2A**). Besides, the group III plants also exhibited delayed flowering time (Supplementary Table S1). These results revealed that decreasing *MTHFR* expression delays switchgrass flowering time without affecting plant normal growth, while strong downregulation of both *MTHFR* and *COMT* affects switchgrass growth and development.

Cross section analysis (without staining) showed obvious dark-brown coloration at each node and basal internode of group III lines compared with wild type and the original *COMT*-RNAi plants (**Figure 2C**). This coloration trend weakens in the younger internode, but it was not reduced in the younger node (**Figure 2C**).

### Effects of *MTHFR* and *COMT* Downregulation on Lignin Content and Lignin Composition

**FIGURE 3:**
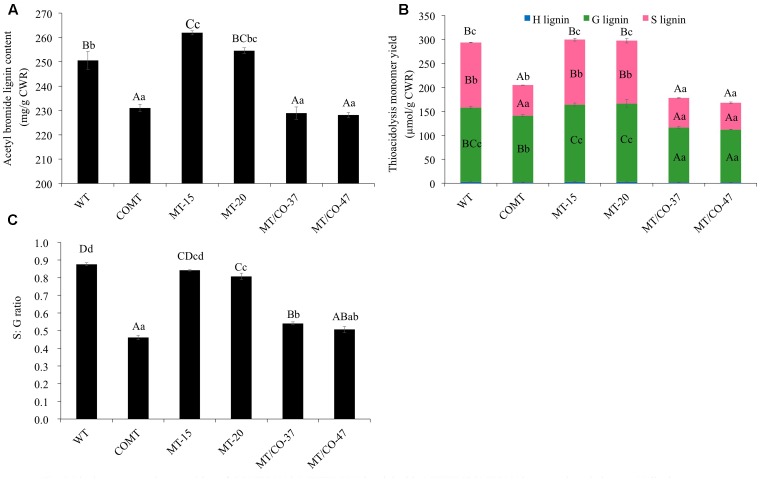
Lignin content and composition of *COMT*-RNAi, *MTHFR*-RNAi and double *MTHFR/COMT*-RNAi transgenic switchgrass. **(A)** Lignin content of transgenic plants as determined by acetyl bromide method, **(B)** lignin composition of transgenic plants as determined by thioacidolysis. S, syringyl unit; G, guaiacyl unit; H, p-hydroxyphenyl unit, **(C)** S: G ratio of transgenic plants from thioacidolysis. CWR, cell wall residue. Values are mean ± SE (*n* = 3). Three independent experiments were carried out for the analyses. Capital or small letters indicate a significant difference of *P* < 0.01 or *P* < 0.05 by one way ANOVA, multiple comparison.

Transgenic lines (MT/CO-37 and MT/CO-47) with heavily downregulated *MTHFR* and *COMT* were subjected to lignin analysis. Single gene knockdown lines COMT, MT-15, and MT-20 were also analyzed. Double transgenic lines showed a significant decrease in AcBr lignin content (8–9%) compared to wild type (**Figure 3A**). The reduction of AcBr lignin content in double gene knockdown plants was similar to that of the *COMT*-RNAi line. Lignin composition was also altered in double transgenic plants. Compared with the wild-type plant, the total thioacidolysis lignin yield, S lignin, G lignin, and S/G ratios, respectively, decreased 39–43%, 55–59%, 27–29%, 38–42% in MT/CO-37 and MT/CO-47 lines. However, compared with the *COMT*-RNAi line, only G lignin showed a reduction (9–13%) in the double gene knockdown lines, while S lignin and H lignin were not altered (**Figure 3B**). Lignin composition, measured by S/G ratio, did not show consistent change in the *MTHFR/COMT*-RNAi lines when compared with the *COMT*-RNAi line (**Figure 3C**). In single *MTHFR* knockdown lines, lignin content was not reduced and lignin composition was not consistently altered (**Figure 3**).

### Induction of Lesion-Mimic-Like Phenotype in Double Gene Knockdown Transgenic Switchgrass

**FIGURE 4:**
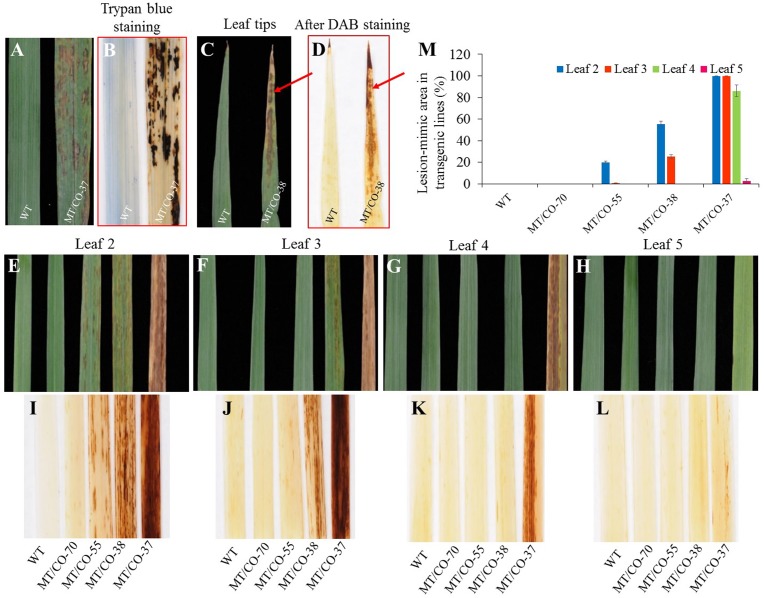
Lesion-mimic-like phenotypes identified on the leaves of double *MTHFR/COMT*-RNAi transgenic switchgrass. **(A)** Lesion-mimic-like phenotype. **(B)** Cell death revealed by trypan blue staining. **(C)** Lesion-mimic-like phenotype initiated from leaf tip. **(D)** Corresponding accumulation of reactive oxygen species on leaf tip as revealed by DAB staining. **(E–H)** Lesion-mimic-like phenotypes observed from the 2nd leaf (leaf 2, old leaf) to the 5th (leaf 5, young leaf) of wild-type and transgenic switchgrass at Rl stage. **(I–L)** Corresponding accumulation of reactive oxygen species on 2nd leaf to 5th leaf revealed by DAB staining. **(M)** Average lesion area per leaf of wild-type and transgenic switchgrass. Values are mean ± SE (*n* = 4). Significance as determined by one way ANOVA.

Under normal greenhouse conditions, a lesion-mimic-like cell death (LMD) phenotype was observed on the leaf surfaces of groups II and III plants (**Figure 4**). The lesion-like necrosis resembled lesions caused by hypersensitive cell death and were spontaneously initiated without any stresses or chemical treatments. When transgenic plants reached E3 or E4 stage, the oldest leaves displayed a lesion phenotype that initiated from the leaf tips and continuously spread over the whole leaves until the oldest leaves became wilted and died (**Figures 4C,E**). Trypan blue staining revealed that these necrotic lesions were formed due to cell death (**Figure 4B**). To further study the development of the necrotic lesions, we selected representative transgenic plants from group I (MT/CO-70), II (MT/CO-38 and MT/CO-55) and III (MT/CO-37) displaying different densities of lesions at R1 stage (**Figures 4E–H**). Lesions were not observed in group I plants. The development of the necrotic lesions in groups II and III plants had similar regular patterns showing that the density of the lesions was much stronger in the older leaves (Leaf 2) compared with the younger leaves (Leaf 5) (**Figures 4E–H**). The areas of lesions from groups II and III plants were substantially higher compared to the wild type (**Figure 4M**). These results indicated the necrotic lesions were developmentally regulated in transgenic switchgrass.

Diaminobenzidine staining showed the accumulation of reddish-brown colorations in the lesions in the groups II and III plants. These staining results were highly associated with the formation of the lesions, indicating that the necrotic lesions corresponded to the over-accumulation of H_2_O_2_ (**Figures 4E–L**).

### Formation of the Lesion-Mimic Cell Death Phenotype Is Regulated by Both *MTHFR* and *COMT* Genes

**FIGURE 5:**
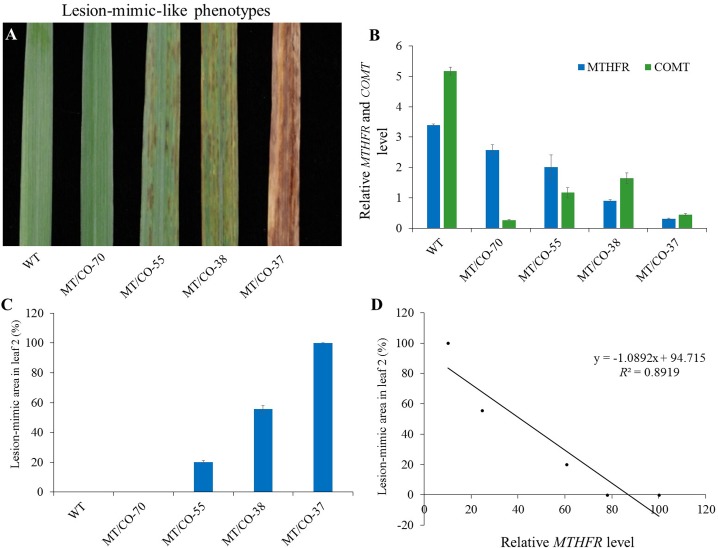
Relationship between the lesion-mimic-like phenotype and the downregulation of single *MTHFR* and double *MTHFR/COMT* in switchgrass. **(A)** Lesion-mimic-like phenotypes of the 2nd leaf in different groups of double gene knockdown switchgrass (group I: 70; group II: 38 and 55; group III: 37). **(B)** Relative expression of *MTHFR* in different groups of plants. **(C)** Average lesion area in the 2nd leaf. **(D)** Correlation analysis between lesion-mimic area and *MTHFR* gene expression level. Values are mean ± SE (*n* = 4). Significance as determined by one way ANOVA.

We found that the density of the lesions in double gene knockdown transgenic switchgrass related to the levels of *MTHFR* and *COMT* expression. Plants with lowest expression of *MTHFR* (10–15% residue level) in the *COMT*-deficient (8% residue level) background (group III plants) showed a high density of lesions; plants with moderate expression of *MTHFR* (25–60%) and *COMT* (18–48%) (group II plants) exhibited a moderate density of lesions (**Figure 5A**). These lesions were not observed in group I and single *MTHFR* knockdown lines (**Figure 5A**). Because it was impossible to obtain the single *MTHFR* transgenic lines with strong downregulation level of *MTHFR*, and, in order to figure out which gene regulated the lesion-mimic-like cell death phenotype, we made the following comparisons (**Figures 1A,B**, **5A**): First, in the *COMT*-RNAi line, even when *COMT* expression decreased more than 90%, there were no lesions, indicating that the downregulation of *COMT per se* was not the reason for lesion formation. Second, the gene expression level of group I plants (no lesions, e.g., MT/CO-70 line) was compared to that of group III plants (severe lesion phenotype, e.g., MT/CO-37 line), *COMT* expression was similar, but *MTHFR* expression level was significantly different (**Figure 5B**), indicating the lesions were caused by suppression of either the *MTHFR* gene alone or by both *MTHFR* and *COMT* genes. Third, the gene expression levels in group II plants were compared to those of single *MTHFR* knockdown lines (e.g., MT-15 line, **Figure 1A**), *MTHFR* expression levels were sometimes similar (e.g., MT/CO-55 line, **Figure 1A**), while *COMT* expression in group II was significantly lower (**Figure 1B**); this suggests that *COMT* also contributed to lesion formation, but only in the case of concurrence with *MTHFR*. Thus, the above evidences suggest that the lesion-mimic-like phenotype is regulated by both *MTHFR* and *COMT* genes. By correlation analysis, we found the area of the lesions in double gene knockdown plants was highly negatively correlated with the expression levels of *MTHFR* gene in the *COMT*-deficient background (*R*^2^ = 0.89) (**Figures 5B–D**), indicating a predominant effect of the *MTHFR* gene in regulating lesion formation.

### Microarray Analysis

**FIGURE 6:**
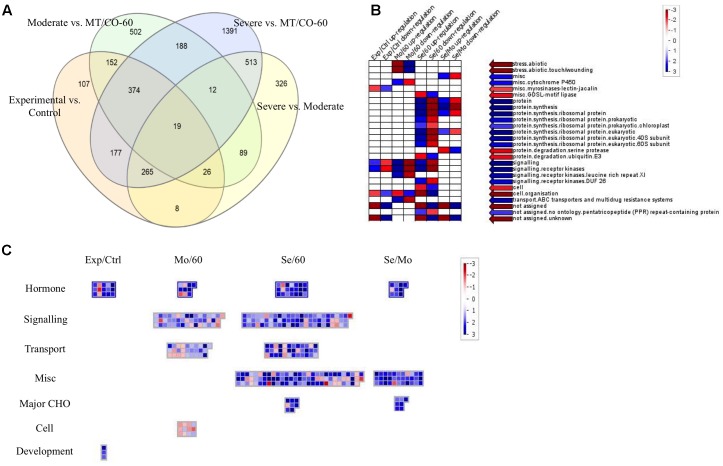
Comparison of differential expressed genes (DEGs) between various groups: severe vs. MT/CO-60, moderate vs. MT/CO-60, experimental vs. control group, and severe vs. moderate. **(A)** Venn diagram showing the commonly up- or down-regulated gene numbers. **(B)** Functional enrichment analysis of DEGs by PageMan. Red and blue color indicates under-represented and over-represented functional group, respectively. **(C)** Significant pathway analysis of DEGs by MapMan. Numbers indicate Log_2_-fold change ratios of pairwise comparison. Blue and red represents upregulated and downregulated genes, respectively.

To investigate the molecular mechanism of *MTHFR* and *COMT* in inducing lesion-mimic-like cell death in switchgrass, representative lines of MT/CO-37, MT/CO-47 (severe lesion phenotype, Se) and MT/CO-38, MT/CO-67 (moderate lesion phenotype, Mo) were selected for microarray analysis. The line MT/CO-60 was used as control because it showed wild-type phenotype and less than 40% reduction in the expression of both *MTHFR* and *COMT* (Supplementary Figure S5); a double gene transformant like this is an ideal control for minimizing the differences in genetic background. Differentially expressed genes were identified using four comparisons (Supplementary Table S2). In the Se/60 group (Severe vs. MT/CO-60), transcript abundance of 2940 genes was altered more than twofold, among which, 1693 genes were upregulated and 321 genes were downregulated. In the Mo/60 group (Moderate vs. MT/CO-60), out of 1361 differentially expressed genes, 409 genes were upregulated and 287 genes were downregulated. Between Se/60 and Mo/60 groups, 201 genes were simultaneously upregulated and 126 genes were simultaneously downregulated (Supplementary Tables S2–S4 and **Figure 6A**). Nine upregulated genes that associated with lesion-mimic-like phenotype among the 201 commonly transcripts were selected for qRT-PCR verification (Supplementary Table S3 and Figure S6). All the selected genes in qRT-PCR analysis showed similar expression patterns with those of the microarray analysis, implying high reliability of the microarray results. Furthermore, transcript levels of these genes were highly correlated with the developmental process of the lesion phenotype (Supplementary Figure S6). To narrow down differentially expressed genes, we designed two extra comparisons, Experimental/Control (mixed data of Se and Mo vs. mixed data of MT/CO-60 and wild type) group and Se/Mo (Severe vs. Moderate) group. Venn diagram analysis showed 19 differentially expressed transcripts in common based on our four comparisons (**Figure 6A**), among which, 13 genes with over 2.5-fold change in Se/60 and Mo/60 groups are shown (**Table 1**). Most of these genes are related to oxidative and defense responses, suggesting the high possibility of these genes triggering the initiation of lesion-mimic-like cell death.

**Table 1:** Differentially expressed genes selected based on Venn diagram analysis.

Gene annotation	Gene ID	Function	Fold-change			
			Severe vs. MT/CO-60	*P*	Moderate vs. MT/CO-60	*P*
Limonoid glucosyltransferase	Pavir.Gb02476	Detoxify virulence factors produced by pathogens.	113.02	0	5.31	4.35E-62
Serine carboxypeptidase I	Pavir.Ia02669	Catalyze the production of secondary plant metabolites involved in host defense.	53.79	0	14.05	0
Unknown	Pavir.Ca00053	–	46.29	0	5.95	9.34E-293
Na+-driven multidrug efflux pump	Pavir.Ia01364	Defense mechanisms	42.52	0	8.59	0
TT virus (TTV)	Pavir.Ab02305	–	33.56	0	3.92	3.30E-97
DNA polymerase III subunits gamma	Pavir.Ea03135	–	30.22	0	3.66	8.40E-172
Horseradish peroxidase	Pavir.Bb03601	Hydrogen peroxide detoxification, auxin catabolism and lignin biosynthesis, and stress response.	21.76	0	4.43	1.73E-47
Cytochrome P450	Pavir.Ab01975	Carotenoid biosynthesis	14.2	0	3.46	0
Glycosyl hydrolase	Pavir.J01050	Degradation of biomass and bacterial walls, remodeling of hyphal walls, protein processing	12.73	0	2.57	5.18E-45
Unknown	Pavir.J27056	Plant–pathogen interaction	12.44	0	3.06	3.49E-26
Unknown	Pavir.Da00733	–	12.26	0	3	3.98E-94
Tic62-NAD(P)-related group II protein	None	Redox sensor, may possibly act as a regulator during the translocation process.	8.49	0	3.43	1.71E-58
ATP-dependent Clp protease proteolytic subunit	Pavir.Ia03797	Plant growth and development, especially for chloroplast function.	8.33	0	2.72	1.11E-203

*Numbers indicate Log_2_-fold change ratios of pairwise comparison. Genes with more than 2.5-fold change from both two comparisons were shown in the table. “–” represents unknown gene function. *P*, Bonferroni-corrected *P*-value*.

To identify possible functions of the altered genes from the four comparisons, functional enrichment analysis by PageMan was performed (**Figure 6B**). In three of these comparisons (Experimental/Control, Mo/60 and Se/60), the up-regulated genes were over-represented in signaling, while the downregulated genes were over-represented in cell organization. The up-regulated genes in Se/60 as well as in the Se/Mo comparisons were also over-represented in protein synthesis. These results indicated that genes involved in signaling and protein synthesis processes were strongly induced in *MTHFR* and *COMT* knockdown switchgrass. To further clarify which metabolic pathway had been significantly affected, the differentially expressed genes from the comparisons were mapped onto MapMan (**Figure 6C**). The data showed that the expression levels of many genes were altered in hormone metabolism, signaling, transport, major carbohydrate (CHO), cell, and development processes (**Figure 6C** and Supplementary Table S5). Among these alterations, most of the genes involved in hormone metabolism were induced in all four comparisons. Additionally, in the biotic stress pathway, marked induction of the genes involved in signaling, JA metabolism and decreased expression of bZIP and MYB-related transcription factors were observed (Supplementary Figure S7 and Table S6), suggesting that these metabolic processes might directly participate or exhibit cross-talk effects in the activation of defense response.

### Metabolic Profiling Was Altered in Transgenic Switchgrass

**FIGURE 7:**
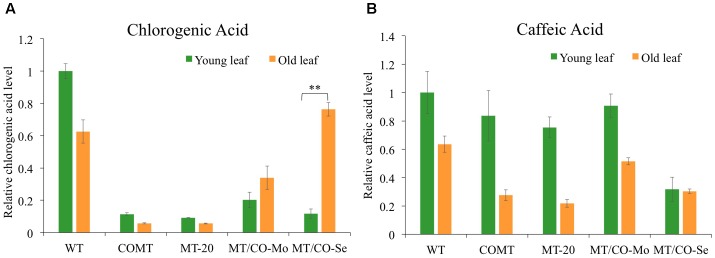
Relative chlorogenic **(A)** and caffeic acid **(B)** levels in young and old leaves of wild-type and transgenic switchgrass. Young leaves (leaf 4) and old leaves (leaf 2) were harvested at E4 stage. MT/CO-Mo or MT/CO-Se means the mixture of two lines from moderate lesion phenotype (group II) or severe lesion phenotype (group III) transgenic switchgrass, respectively. Values are mean ± SE (*n* = 3). Two asterisks indicate significant difference of *P* < 0.01 by one way ANOVA.

Gas chromatography mass spectrometry analysis revealed significant alterations of phenolic compounds in transgenic plants relative to wild type. Chlorogenic acid showed 80–90% decrease in young leaves of MT/CO-Se (group III plants with severe phenotype), MT/CO-Mo (group II plants with moderate phenotype) lines, as well as COMT and MT-20 lines (**Figure 7A**), indicating that perturbation of either *MTHFR* or *COMT* gene, or the two genes together, markedly affected the accumulation of chlorogenic acid in young leaves of switchgrass. In wild type, chlorogenic acid accumulated less in old leaves compared to young leaves, and the single *MTHFR*-RNAi (MT-20) and *COMT*-RNAi (COMT) lines showed a similar trend (**Figure 7A**). However, the trend is reversed in the double gene downregulation lines with MT/CO-Mo and MT/CO-Se, respectively, displaying 1.7- and 7-fold increased accumulation of chlorogenic acid in old leaves relative to that in young leaves (**Figure 7A**). Another soluble phenolic compound, caffeic acid, showed decreased accumulation of 70% only in young leaves of MT/CO-Se line compared to WT (**Figure 7B**). The accumulation of caffeic acid was lower in old leaves than in young leaves in most transgenic lines except MT/CO-Se, in which caffeic acid accumulated at almost the same level in young and old leaves (**Figure 7B**). The above data indicate that synthesis of phenolic compounds was affected by the downregulation of *MTHFR* and *COMT* in switchgrass, particularly when comparing the relative levels of young and old leaves.

The overall amino acid pools were not notably altered in young leaves except for Ile, an Asp family amino acid, which showed a slightly elevated accumulation in MT/CO-Mo and MT/CO-Se lines (Supplementary Table S7). In contrast to the metabolic patterns in young leaves, marked increased accumulation of Asp, Ile, Leu, Phe, Pro, Tyr, Glu, Ser, and Thr metabolism was observed in old leaves of both MT/CO-Se and MT/CO-Mo lines (Supplementary Tables S7, S8). Specifically, compared to WT the relative abundance of Ser increased by 28 and 6-fold in MT/CO-Se and MT/CO-Mo lines, respectively, and Glu increased by 2.6 and 2.5-fold (Supplementary Table S8). Moreover, Pro, which functions as an osmoprotectant, displayed 1.5–5-fold increased accumulation (Supplementary Table S7). Notably, elevated levels of most amino acids in old leaves were correlated with the intensity of the lesions in MT/CO-Se and MT/CO-Mo lines, indicating that the alteration of amino acid metabolism is related to lesion formation.

Furthermore, the main sugars including glucose, galactose, sucrose, and fructose showed markedly increased accumulations in old leaves of MT/CO-Se lines (Supplementary Table S8). Specifically, glucose and fructose levels increased by 57 and 12-fold relative to WT plants, respectively. However, most of the sugars except galactosyl glycerol did not show significant change in leaves of the MT/CO-Mo lines (Supplementary Table S8), suggesting that the sugar metabolism was mainly affected in plants with strong lesion-mimic-like phenotypes.

## Discussion

Grass lignin is composed of three hydroxycinnamyl alcohol units (coniferyl, sinapyl alcohol, and p-coumaryl alcohol), leading to the formation of three guaiacyl (G), syringyl (S), and p-hydroxyphenyl (H) types of lignin subunits (Barrière et al., 2007; Hatfield et al., 2017). Downregulation of *COMT* in switchgrass resulted in significant reduction of lignin content, which led to large increase in biofuel production (Fu et al., 2011; Baxter et al., 2014). Because MTHFR is directly involved in the production of the methyl donor, SAM, and lignin content is reduced in the maize *bm2* mutant, it was expected that simultaneous downregulation of *COMT* and *MTHFR* would lead to further alteration of lignin biosynthesis. However, strong downregulation of both *MTHFR* and *COMT* in switchgrass did not show any additive effect on lignin modification. Lignin content of the *MTHFR*/*COMT* double transformants was similar to that of the single *COMT* knockdown plants, indicating that when COMT activity is low, SAM is no longer a limiting factor in the process of lignin biosynthesis. Among the three lignin units, G lignin were moderately reduced in the double transformants. In the *O*-methylation reactions, except COMT, the methyl units derived from the C1 pathway are also consumed by caffeoyl-CoA *O*-methyltransferase (CCoAOMT). Previous reports have shown that downregulation of *CCoAOMT* led to reduction mainly in G lignin units in different dicot species (Chen et al., 2001; Guo et al., 2001; Pinçon et al., 2001). However, in switchgrass, the *CCoAOMT*-suppressed transgenics with approximately 10% residue activity displayed no changes in either lignin content or composition (Shen et al., 2013). Thus the role of *CCoAMT* in lignin biosynthesis is not clear in monocot species. Our results showed that the transcript levels of *CCoAOMT1* were significantly reduced in *MTHFR* and *MTHFR*/*COMT* double transformants, although there was a large variation in the levels of reduction in the double transformants (Supplementary Figure S10). To further confirm the impact of *MTHFR* downregulation, it would be interesting to analyze AdoMet level in the transgenic switchgrass in the future.

Both *COMT* knockdown switchgrass and a *bm2* maize mutant showed normal agronomic performance (Fu et al., 2011; Baxter et al., 2014; Tang et al., 2014). Phenotypically, it was observed that lower internodes of *COMT* knockdown switchgrass showed brownish color (Fu et al., 2011) while the maize *bm2* mutant had brownish color in the midribs of leaves (Tang et al., 2014). In the *MTHFR*/*COMT* double knockdown plants, no color change was observed in leaf midribs, however, dark brown coloration was found in nodes and internodes (**Figure 2C**). The coloration is much stronger than that of *COMT* knockdown switchgrass plants. The results show that *COMT* and *MTHFR* do have an additive impact on the accumulation of coloration compounds. Identification of the compounds remains to be an interesting topic for future research.

Unexpectedly, leaves of the *MTHFR*/*COMT* double knockdown plants developed necrotic lesions. After cutting the plants back, newly developed tillers still displayed such lesions, excluding the possibility that the lesions were associated with disease. No such phenotype was reported in the maize *bm2-bm3* (*MTHFR-COMT*) double mutant or other combinations of *bm* mutants. Because the lesions are spontaneous in the absence of pathogen infection, they are called lesion mimics. The spread of necrotic lesions in switchgrass is associated with plant development, indicating lesion formation may result from the activation of a programmed cell death (PCD) pathway. Consistent with the developmental pattern of the lesions, H_2_O_2_ is accumulated closely around the lesions on the leaves, suggesting that lesion formation is triggered by the alteration of levels of endogenous reactive oxygen species (ROS).

To clarify whether the lesion-mimic cell death phenotype was induced by single *MTHFR* silencing or double gene co-silencing, we classified transgenic switchgrass into three groups based on gene expression level and density of lesions. Comparisons between different groups showed that *MTHFR* and *COMT* synergistically induced lesions in switchgrass, suggesting an interactive effect of *MTHFR* and *COMT* in the cell death pathway. Although lacking any direct connections between lesion-mimic cell death phenotype and either of the two genes, previous studies did show that they had similar expression patterns at either transcriptional or protein level in response to defense. A proteome analysis of the cell death and resistance (*cdr2*) mutant in rice characterized differentially regulated proteins. Among these proteins, defense-related enzyme COMT and metabolic enzymes MTHFR and SAM2 showed similar up-regulated expression patterns, indicating that the PCD is associated with defense and active metabolic changes (Tsunezuka et al., 2005). Bhuiyan et al. (2007) reported that with the infection of *Blumeria graminis* f. sp. *tritici* (*Bgt*), the wheat *COMT* gene displayed a concomitant expression pattern with the genes involved in C1 pathway. In our study, *MTHFR* affects the lesion formation in a dose-dependent manner, thus we conclude that *MTHFR* plays a predominant role in inducing the lesion-mimic cell death phenotype. SAM, located downstream of MTHFR, functions as a precursor of ethylene (Moffatt and Weretilnyk, 2001), which has been reported to play a key role in controlling cell death and defense response in an Arabidopsis lesion mimic mutant *vad1* (Bouchez et al., 2007). A photorespiration-related gene serine hydroxymethyltransferase (*SHMT1*), catalyzing the conversion between serine and 5,10-methylene-THF, which serve as the substrate for MTHFR (Supplementary Figure S8), plays a crucial role in restricting pathogen-induced cell death (Moreno et al., 2005). Therefore, the expression levels of switchgrass *SAMS*, 1-aminocyclopropane-1-carboxylate oxidase (*ACO*) (the hallmark gene in ethylene biosynthesis) and *SHMT1* were examined. However, none of these gene expressions were correlated with lesion formation (Supplementary Figure S9). Thus, *MTHFR* and *COMT* mediated lesion-mimic cell death may go through a new pathway that has not been reported.

Transcriptomics and metabolomics analyses provide clues to decipher the relationships between lesion formation and gene functions. Microarray analysis identified some oxidative and defense related genes that are highly associated with the lesion-mimic cell death phenotype. Our results showed the lesion formation is the consequence of over-accumulation of H_2_O_2_. Given the crucial role of ROS in the initiation phase of different plants’ PCD (Mittler and Rizhsky, 2000; Van Breusegem and Dat, 2006), *MTHFR* and *COMT* may be involved in ROS-dependent cell death. It is well known that ROS are produced from enhanced enzymatic activity of plasma membrane-bound NADPH oxidases, cell-wall-bound peroxidases, and apoplastic amine oxidases during oxidative burst (Lamb and Dixon, 1997; Bolton, 2009). It has also been shown that *peroxidase* genes are involved ROS generation and cell death activation (Choi et al., 2007). Our microarray and qRT-PCR data demonstrated that the *horseradish peroxidase* gene was significantly induced. Horseradish peroxidase belongs to the class III plant peroxidases (Prxs) (EC 1.11.1.7), which are involved in a broad range of physiological processes (Almagro et al., 2009). Prxs mediates the oxidative coupling of three lignin subunits using H_2_O_2_ as the oxidant (Barceló,, 1997). Significantly altered lignin in *MTHFR*/*COMT* modified transgenic plants may result in surplus accumulation of Prxs and further induced H_2_O_2_. On the other hand, it has been reported that ROS has a strong interplay with other signaling molecules (phytohormones) during plant PCD (Overmyer et al., 2005; Van Breusegem and Dat, 2006). Hence, the striking up-regulation of genes involved in receptor kinases signaling and protein synthesis revealed by functional enrichment analysis suggests that the active defense response may be linked with ROS accumulation. Similarly, strong induction of hormone metabolism observed by pathway analysis is possibly the result of cross talk between phytohormones and ROS signal (Van Breusegem and Dat, 2006).

Phenolic acid metabolism in plants provides the precursors for lignin biosynthesis (Tamagnone et al., 1998), meanwhile, it also protects plants against oxidative damage (Schnablová, 2006). Disruption of monolignol production affects phenolic acid accumulation and further decreases plant defense ability (Tamagnone et al., 1998; Shen et al., 2012). In tobacco, inhibition of phenolic acid derivatives, especially chlorogenic acid, which is the most abundant phenolic ester, accounts for the induction of premature lesion-mimic cell death (Tamagnone et al., 1998). The underlying mechanism could be that the deficiency of antioxidants leads to an increase in the concentration of ROS above the threshold necessary to trigger cell death (Tamagnone et al., 1998). This also explains why lesions were more prevalent in mature and senescing leaves, where ROS were increasingly accumulated (Thompson et al., 1987; Prochazkova and Wilhelmova, 2007). However, the mechanism of inducing lesion-mimic cell death may be different in switchgrass. Although significantly decreased accumulation of chlorogenic acid was observed in young leaves of *MTHFR*/*COMT* modified plants, it was also reduced in single gene knockdown lines (**Figure 7**), where lesions were not observed. This suggests that in switchgrass, lesion-mimic-like phenotype is not caused by phenolics depletion. The total amino acids pool was not disturbed by modification of *MTHFR* and *COMT* genes in switchgrass. However, significant increase of amino acids was observed in old leaves of transgenics, especially in the MT/CO-Se line with strong lesion-mimic-like phenotype. This result indicates that the alterations of amino acids may be the consequence of active defense response. The strongly increased sugar metabolism in transgenic switchgrass probably functions as the supplier to replenish the depleted energy and carbon required for response to oxidative stress (Dauwe et al., 2007; Bolton, 2009). It has been suggested that allocating resources toward defense response occurs at the expense of plant fitness (Bolton, 2009). Substantial alterations in primary metabolism and gene expression in response to defense may compromise plant growth and development. Double transformants with severe lesion-mimic-like phenotype displayed reduced plant height, decreased dry matter biomass and delayed flowering (**Figures 2A,B** and Supplementary Table S1), suggesting strong co-silencing of *MTHFR* and *COMT* induced secondary effects in switchgrass.

In summary, unexpected results were obtained when co-silencing *MTHFR* and *COMT* in switchgrass. No significant additive effect between *MTHFR* and *COMT* was observed regarding lignification. However, *MTHFR* and *COMT* synergistically caused lesion-mimic-like cell death, and *MTHFR* played a predominant role in the process, suggesting a cross talk between the MTHFR-mediated C1 pathway and secondary metabolism (Supplementary Figure S8). In addition, simultaneous downregulation of *MTHFR* and *COMT* negatively affects plant growth and development.

## Author Contributions

SL, Z-YW, and YZ designed research. SL, CF, JG, and DH performed research. SL, JG, LS, Z-YW, and YZ analyzed data. SL, Z-YW, and YZ wrote the paper.

## Conflict of Interest Statement

The authors declare that the research was conducted in the absence of any commercial or financial relationships that could be construed as a potential conflict of interest.

## Footnotes

1 http://switchgrassgenomics.noble.org

2 http://bioinfogp.cnb.csic.es/tools/venny/

3 http://www.amdis.net/

4 https://www.r-project.org/
