# Supplementary material for: Simultaneous Downregulation of MTHFR and COMT in Switchgrass Affects Plant Performance and Induces Lesion-Mimic Cell Death
Source: Front Plant Sci. 2017 Jun 20;8:982. doi: 10.3389/fpls.2017.00982 (PMC5476930; doi:10.3389/fpls.2017.00982)
Supplement: Supplementary file 1 [file Presentation_1.PDF]

```
                         1         10        20        30        40        50        60
                         |         |         |         |         |         |         |
 ZmMTHFR GRMZM2G347056   ------------MKVIEKILEAAGD-GRTAFSFEYFPPKTEEGVENLFERMDRMVAHGPS
 ZmMTHFR GRMZM2G034278   MCMLLRKDSGHYLAIVVYVKCCSLEEERRK---ERIPTEL---MRSFILTSHTAPGRAPA
 PvMTHFR Pavir.Ia00159   ------------MKVIEKIQQAAAD-GRTAFSFEYFPPKTDEGVENLFERMDRMVAHGPS
OsMTHFR LOC_Os03g60090   ------------MKVIEKIQEAAAD-GRTVFSFEYFPPKTEEGLDNLFERMDRMVAHGPN
  Nt MTHFR2 AEL33268.1   ------------MKVIEKIQEAAKDENRVVFSFEFFPPKTEDGVENLFERMERMVAHNPS
  Nt MTHFR1 AEL33267.1   ------------MKVIEKIQEAAKDENRVVFSFEFFPPKTEDGVENLFERMERMVAHNPS
 MtMTHFR Medtr1g040105   ------------MKVIDKIRSANADPNKVVFSFEFFPPKTEDGVDNLFERMDRMVAHNPS
    AtMTHFR2 AT2G44160   ------------MKVIDKIQSLA-DEGKTAFSFEFFPPKTEDGVDNLFERMDRMVAYGPT
    AtMTHFR1 AT3G59970   ------------MKVVDKIKSVT-EQGQTAFSFEFFPPKTEDGVENLFERMDRLVSYGPT
                                                          *  *                     *

 ZmMTHFR GRMZM2G347056   FCDITWGAGGSTADLTLEIANRMQNMVCVETMMHLTCTNMPVEKIDHALETIKSNGIQNV
 ZmMTHFR GRMZM2G034278   ASSICNDRTRRRAELLSSYIYNSSTKVCVETMMHLTCTNMPVEKIDHALETIKFNGIHNV
 PvMTHFR Pavir.Ia00159   FCDITWGAGGSTADLTLEIANRMQNMVCVETMMHLTCTNMPVEKIDHALETIKSNGIQNV
OsMTHFR LOC_Os03g60090   FCDITWGAGGSTADLTLEIANRMQNMVCVETMMHLTCTNMPVEKIDDALTTIKSNGIQNV
  Nt MTHFR2 AEL33268.1   FCDITWGAGGSTADLTLEISKRMQNMVCVETMMHLTCTNMPVEKIDHALDTIKINGIQNV
  Nt MTHFR1 AEL33267.1   FCDITWGAGGSTADLTLEISKRMQNMVCVETMMHLTCTNMPVEKIDHALDTIKINGIQNV
 MtMTHFR Medtr1g040105   FCDITWGAGGTTADLTLEIANKMQNIICVETMMHLTCTNMPVDKIDHALETIKSNGIQNV
    AtMTHFR2 AT2G44160   FCDITWGAGGSTADLTLDIASRMQNVVCVESMMHLTCTNMPVEKIDHALETIRSNGIQNV
    AtMTHFR1 AT3G59970   FCDITWGAGGSTADLTLEIASRMQNVICVETMMHLTCTNMPIEKIDHALETIRSNGIQNV
                            *   *      *            *** **********  *** ** **  *** **

 ZmMTHFR GRMZM2G347056   LALRGDPPHGQDKFVQVEGGFACALDLVQHIRAKYGDYFGITVAGYPEAHPDAIQGEGGA
 ZmMTHFR GRMZM2G034278   LALRGDPPHGQDKFVQVEGGFACALDLVQHIRSKYGDYFGITVAGYPEAHPDAIQGEGGA
 PvMTHFR Pavir.Ia00159   LALRGDPPHGQDKFVQVEGGFACALDLVQHIRAKYGDYFGITVAGYPEAHPDAIQGEGGA
OsMTHFR LOC_Os03g60090   LALRGDPPHGQDKFVQVAGGFACALDLVQHIRAKYGDYFGITVAGYPEAHPDAIQSTEGA
  Nt MTHFR2 AEL33268.1   LALRGDPPHGQDKFVQVEGGFACALDLVKHIRAKYGDYFGITVAGYPEAHPDVIPANGIA
  Nt MTHFR1 AEL33267.1   LALRGDPPHGQDKFVQVEGGFACALDLVKHIRAKYGDYFGITVAGYPEAHPDVIPANGIA
 MtMTHFR Medtr1g040105   LALRGDPPHGQEKFVQTEGGFACARDLVQHIRSKYGDYFGITIAGYPEAHPDVIGADGLA
    AtMTHFR2 AT2G44160   LALRGDPPHGQDKFVQVEGGFDCALDLVNHIRSKYGDYFGITVAGYPEAHPDVIGENGLA
    AtMTHFR1 AT3G59970   LALRGDPPHGQDKFVQVEGGFACALDLVNHIRSKYGDYFGITVAGYPEAHPDVIEADGLA
                         *********** ****  *** ** *** *** ********* ********* *     *

 ZmMTHFR GRMZM2G347056   TLEAYSNDLAYLKRKVDAGADLIVTQLFYDTDIFLKFVNDCRQIGITCPIVPGIMPINNY
 ZmMTHFR GRMZM2G034278   TLEAYSNDLAYLKRKVDAGADLIVTQLFYDTDIFLKFVNDCRQIGITCPIVPGIMPINNY
 PvMTHFR Pavir.Ia00159   TLEAYTNDLAYLKRKVDAGADLIVTQLFYDTDIFLKFVNDCRQIGITCPIVPGIMPINNY
OsMTHFR LOC_Os03g60090   TPEAYSNDLAYLKQKVDAGADLIITQLFYDTDIFLKFVNDCRQIGITCPIVPGIMPINNY
  Nt MTHFR2 AEL33268.1   TQEIYENDLAYLKRKVDAGADLIVTQLFYDTDIFLKFVNDCRQLGITCPIVPGIMPINNY
  Nt MTHFR1 AEL33267.1   TQEIYENDLAYLKRKVDAGADLIVTQLFYDTDIFLKFVNDCRQIGITCPIVPGIMPINNY
 MtMTHFR Medtr1g040105   TAEGYQSDLAYLKSKVDAGADLIVTQLFYDTDIFLKFVNDCRQIGITCPIVPGIMPINNY
    AtMTHFR2 AT2G44160   SNEAYQSDLEYLKKKIDAGADLIVTQLFYDTDIFLKFVNDCRQIGISCPIVPGIMPINNY
    AtMTHFR1 AT3G59970   TPESYQSDLAYLKKKVDAGADLIVTQLFYDTDIFLKFVNDCRQIGINCPIVPGIMPISNY
                           * *  ** *** * ******* ******************* ** ********** **

 ZmMTHFR GRMZM2G347056   KGFLRMTGFCKTKIPSEITAALDPIKDNEEAVRQYGIHLGTEMCKKILATGIKTLHLYTL
 ZmMTHFR GRMZM2G034278   KGFMRMTGFCKTKIPSEITAALDPIKDNEEAVRAYGIHLGTEMCKKIIASGIKTLHLYTL
 PvMTHFR Pavir.Ia00159   KGFVRMTGFCKTKIPAEITAALDPIKDNEEAVKAYGIHLGTEMCKKILASGIKTLHLYTL
OsMTHFR LOC_Os03g60090   KGFLRMTGFCKTKIPAEITAALEPIKDNEEAVKAYGIHLGTEMCKKILATGIKTLHLYTL
  Nt MTHFR2 AEL33268.1   KGFLRMTGFCKTKIPEEIMAALEPIKDNEEAVKAYGIHLGTEMCKKILASGIKTLHLYTL
  Nt MTHFR1 AEL33267.1   KGFLRMTGFCKTKIPEEIMAALEPIKDSEEAVKAYGIHLGTEMCKKILASGIKTLHLYTL
 MtMTHFR Medtr1g040105   KGFIRMTGFCKTKIPAEIMAALEPIKDNEEAVKAYGIHLGTEMCKKIMAHGIKTVHLYTL
    AtMTHFR2 AT2G44160   RGFLRMTGFCKTKIPVEVMAALEPIKDNEEAVKAYGIHLGTEMCKKMLAHGVKSLHLYTL
    AtMTHFR1 AT3G59970   KGFLRMAGFCKTKIPAELTAALEPIKDNDEAVKAYGIHFATEMCKKILAHGITSLHLYTL
                          ** ** ******** *  *** ****  ***  ****  ******  * *    *****

 ZmMTHFR GRMZM2G347056   NMDKSAIGILMNLGLIEESKVSRPLPWRPATNVFRVKEDVRPIFWANRPKSYLKRTLGWD
 ZmMTHFR GRMZM2G034278   NVDKSALGILMNLGLIEESKVSRSLPWRPATNVFRVKEVVRPIFWASRPKSYLKRTLGWD
 PvMTHFR Pavir.Ia00159   NMEKSALAILMNLGLIEESKVSRPLPWRPPTNVFRVKEDVRPIFWANRPKSYITRTLGWD
OsMTHFR LOC_Os03g60090   NMEKSALGILMNLGLIEESKISRSLPWRPPTNVFRVKEDVRPIFWANRPKSYISRTLGWD
  Nt MTHFR2 AEL33268.1   NMEKSALAILMNLGLIEESKISRPLPWRRPTNVFRVKEDVRPIFWANRPKSYISRTLGWD
  Nt MTHFR1 AEL33267.1   NMEKSALSILMNLGLIEESKISRPLPWRRPTNVFRVKEDVRPIFWANRPKSYISRTLGWD
 MtMTHFR Medtr1g040105   NMEKSALAILTSLGLIEESKISRSLPWRRPTNVFRVKEDVRPIFWANRPKSYISRTIGWE
    AtMTHFR2 AT2G44160   NMEKSALAILMNLGMIDESKISRSLPWRRPANVFRTKEDVRPIFWANRPKSYISRTKGWE
    AtMTHFR1 AT3G59970   NVDKSAIGILMNLGLIDESKISRSLPWRRPANVFRTKEDVRPIFWANRPKSYISRTKGWN
                         *  ***  **  ** * *** ** ****   **** ** ******* *****  ** **

 ZmMTHFR GRMZM2G347056   QYPH--GRWGDSRNPSY-GALTDHQFTRPRGRGKKLQEEWAVPLKSVEDISERFTNFCQG
 ZmMTHFR GRMZM2G034278   QYPHEGGVILETHHMEHLGIVHKTTWTW*-------------------------------
 PvMTHFR Pavir.Ia00159   QYPH--GRWGDSRNPSY-GALTDHQFTRPRGRGKKLQEEWAVPLKSVEDISERFTNFCEG
OsMTHFR LOC_Os03g60090   QYPH--GRWGDSRNPSY-GALTDYQFTRPRGRGKKLQEEWAVPVKSVEDINERFMNFCQG
  Nt MTHFR2 AEL33268.1   EYPH--GRWGNAQNPSY-GALTDYQFMRARSRDKKLQEEWAVALNSVDDIYERFKDYCLG
  Nt MTHFR1 AEL33267.1   EYPH--GRWGNAQNPSY-GALSDYQFMRARSRDKKLQEEWVVALNSVEDTYEKFKDYCLG
 MtMTHFR Medtr1g040105   QYPH--GRWSDSGNPSY-GALTDYQFMRPRAKDKKLIEEWAVPLKSIEDIYERFRLFCLG
    AtMTHFR2 AT2G44160   DFPQ--GRWGDSRSASY-GALSDHQFSRPRARDKKLQQEWVVPLKSVEDIQEKFKELCLG
    AtMTHFR1 AT3G59970   DFPH--GRWGDSHSAAY-STLSDYQFARPKGRDKKLQQEWVVPLKSIEDVQEKFKELCIG
```

Supplementary Fig. S1 Multiple alignment of switchgrass MTHFR amino acid sequences with maize, rice, tobacco, *Arabidopsis* and *Medicago truncatula* orthologs. Alignment was performed using geneious (Geneious version 7.0 created by Biomatters. Available from http://www.geneious.com). The yellow color highlighted the amino acid sequences that were used to construct MTHFR-RNAi vector. * indicates the conversed amino acid of MTHFR among monocot and dicot species. Amino acid sequences were downloaded from phytozome (https://phytozome.jgi.doe.gov/pz/portal.html).

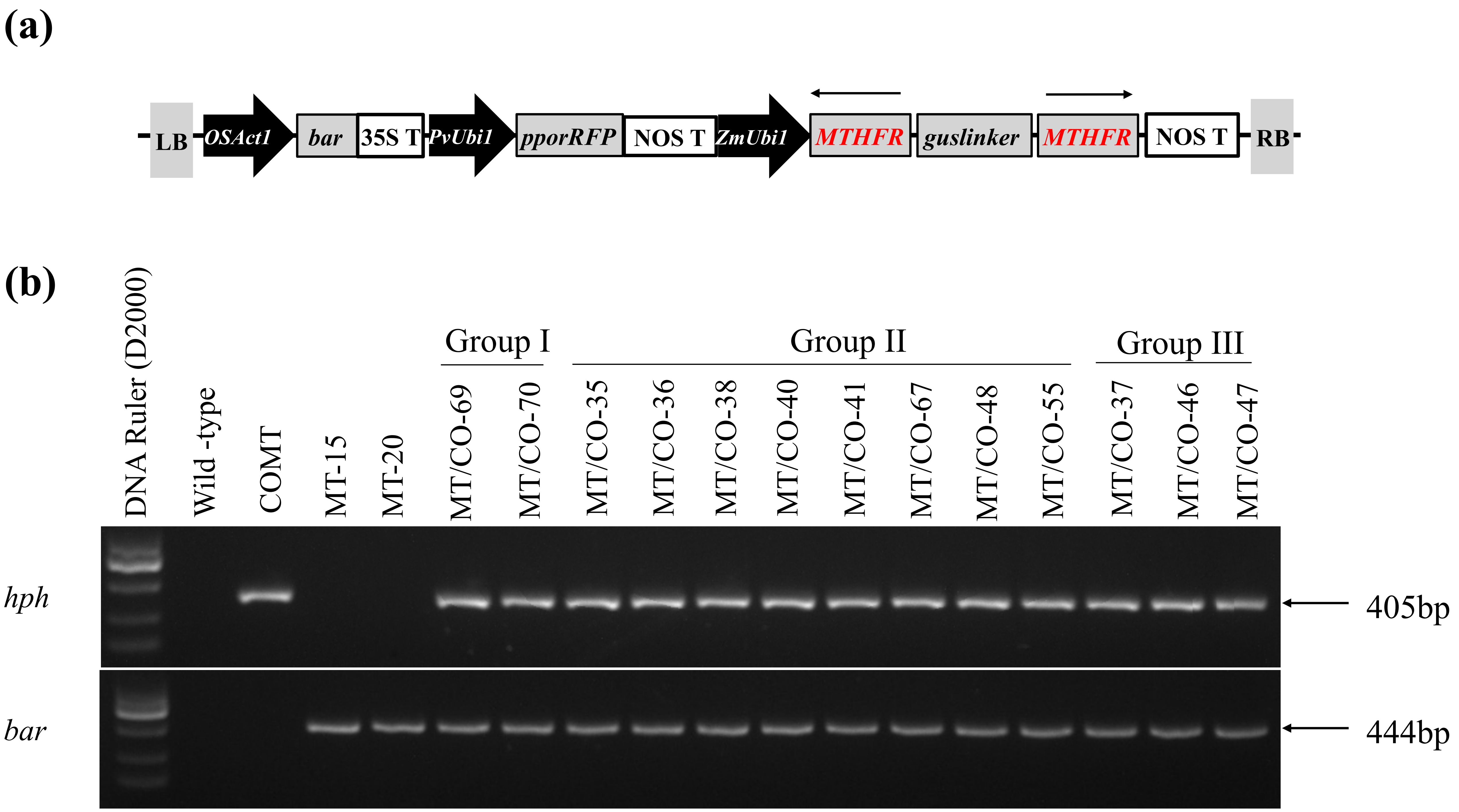

Supplementary Fig. S2 Vector construction and molecular identification. (a) A 506-bp *MTHFR* fragment was cloned into the binary vector pANIC 8D. Arrows indicates the direction of the fragment. (b) Positive transgenic plants with different selectable marker genes were identified by PCR amplification of switchgrass genomic DNA.

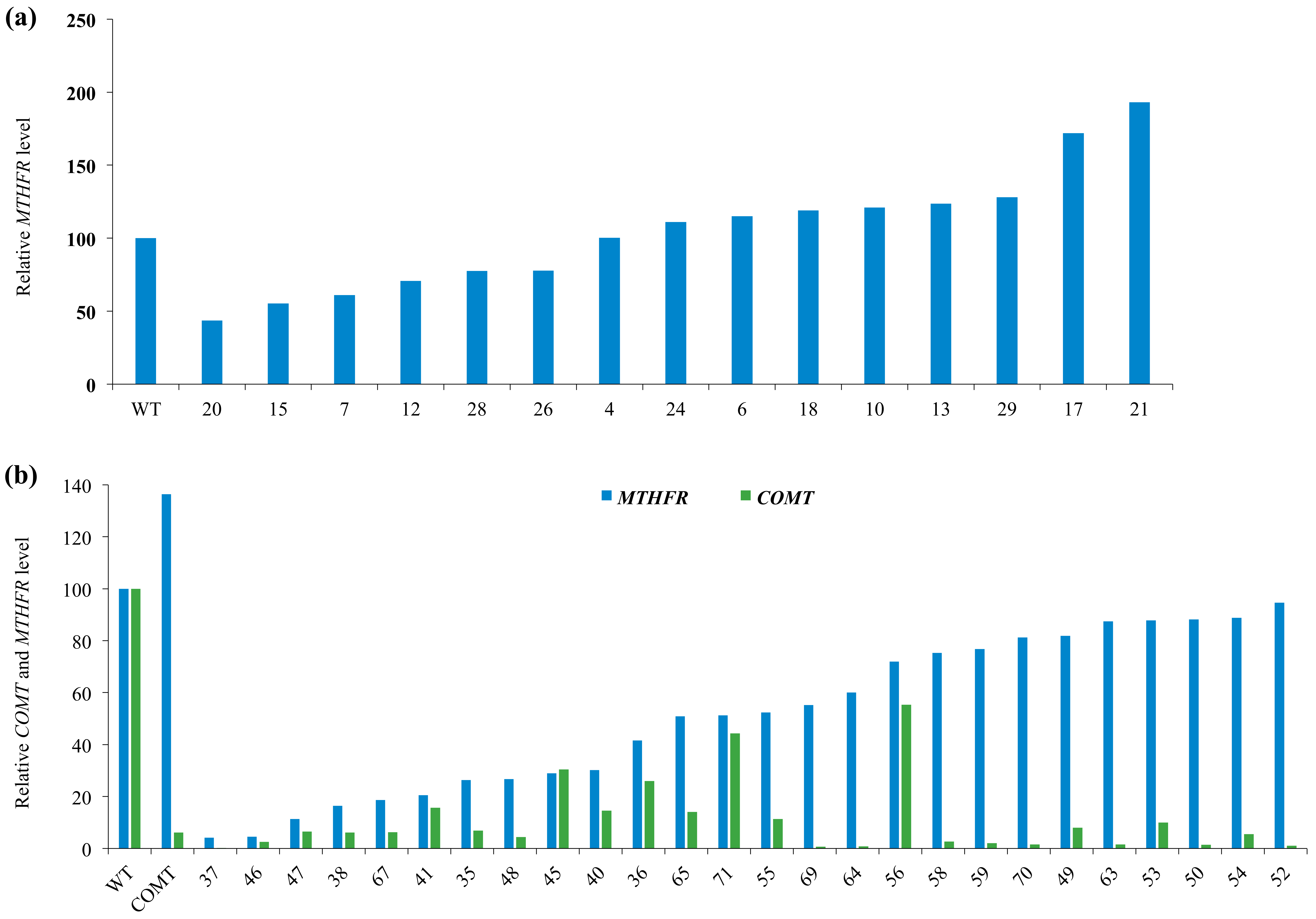

Supplementary Fig. S3 Relative transcript levels of *MTHFR* in single *MTHFR* knockdown transgenics (a) and relative transcript levels of *MTHFR*/*COMT* in double gene knockdown transgenics (b). *Ubiquitin1* was used as the reference gene.

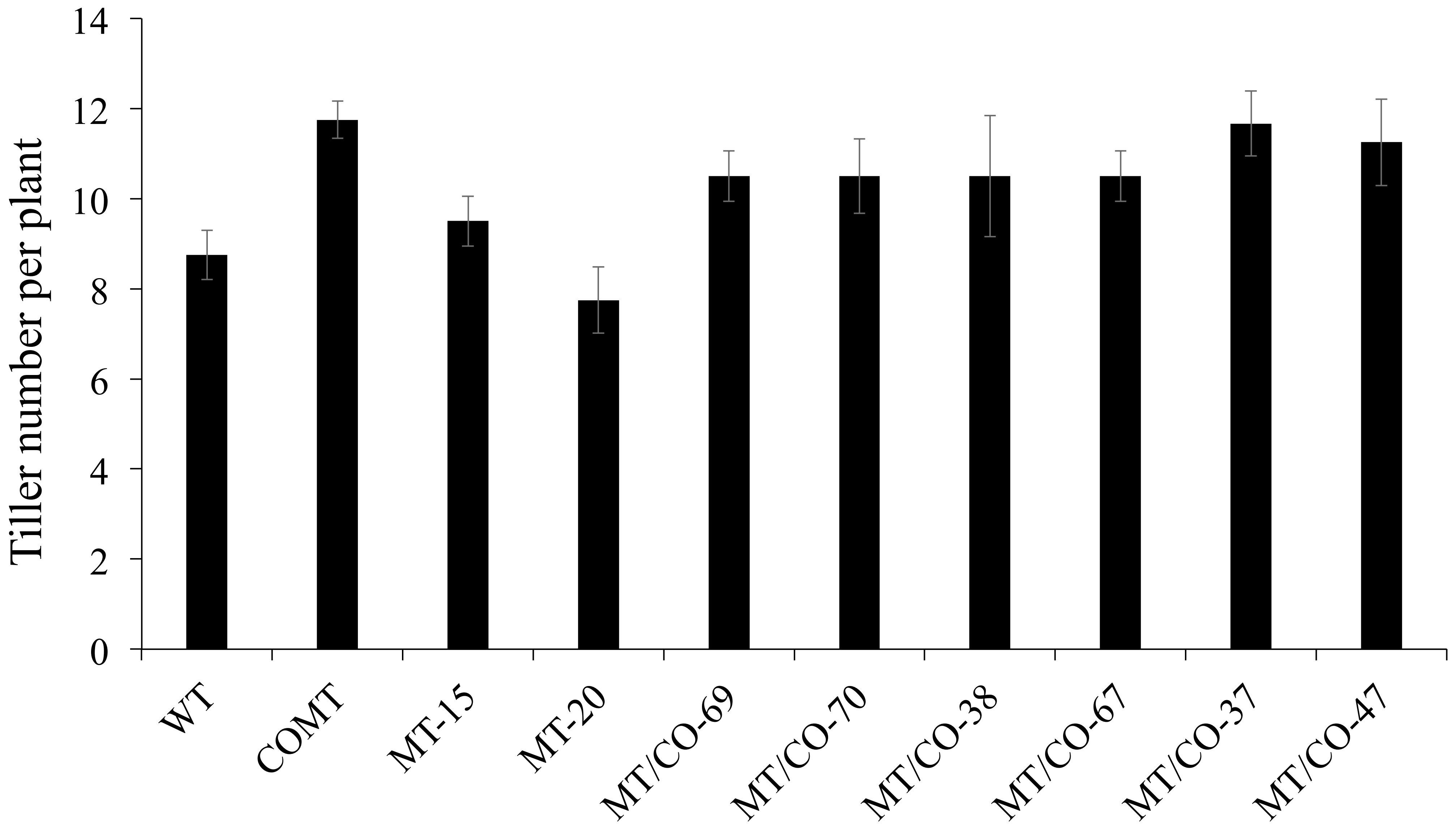

Supplementary Fig. S4 Tiller number of wild-type (WT) and transgenic plants at R1 stage.

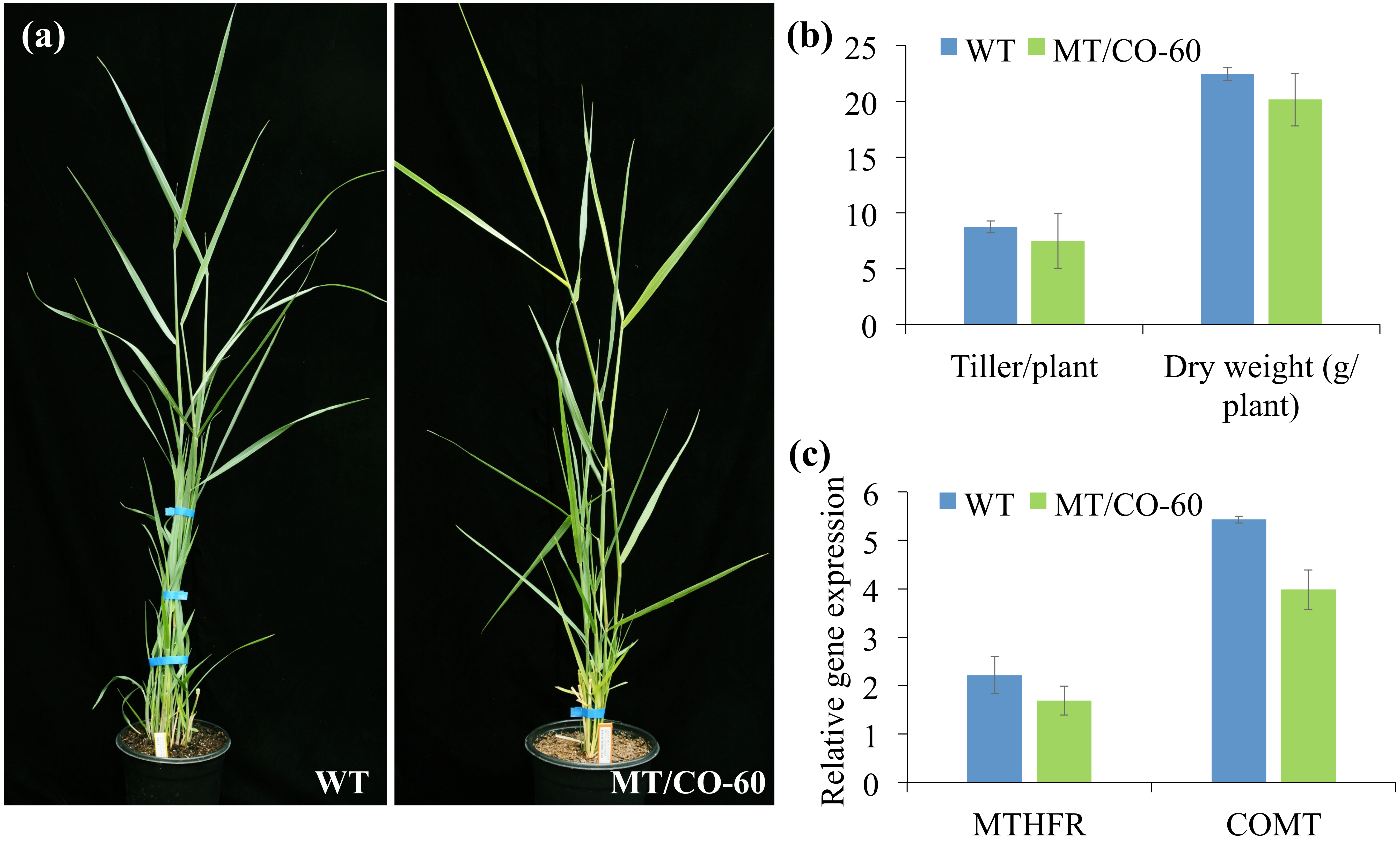

Supplementary Fig. S5 Comparison of phenotype, dry matter biomass and gene expression between MT/CO-60 line and wild-type plant. (a) Morphological characterization of WT and MT/CO-60 line at R1 stage. (b) Tiller number and dry matter biomass per plant. (c) Relative expression level of *MTHFR* and *COMT* in MT/CO-60 line and wild-type plant.

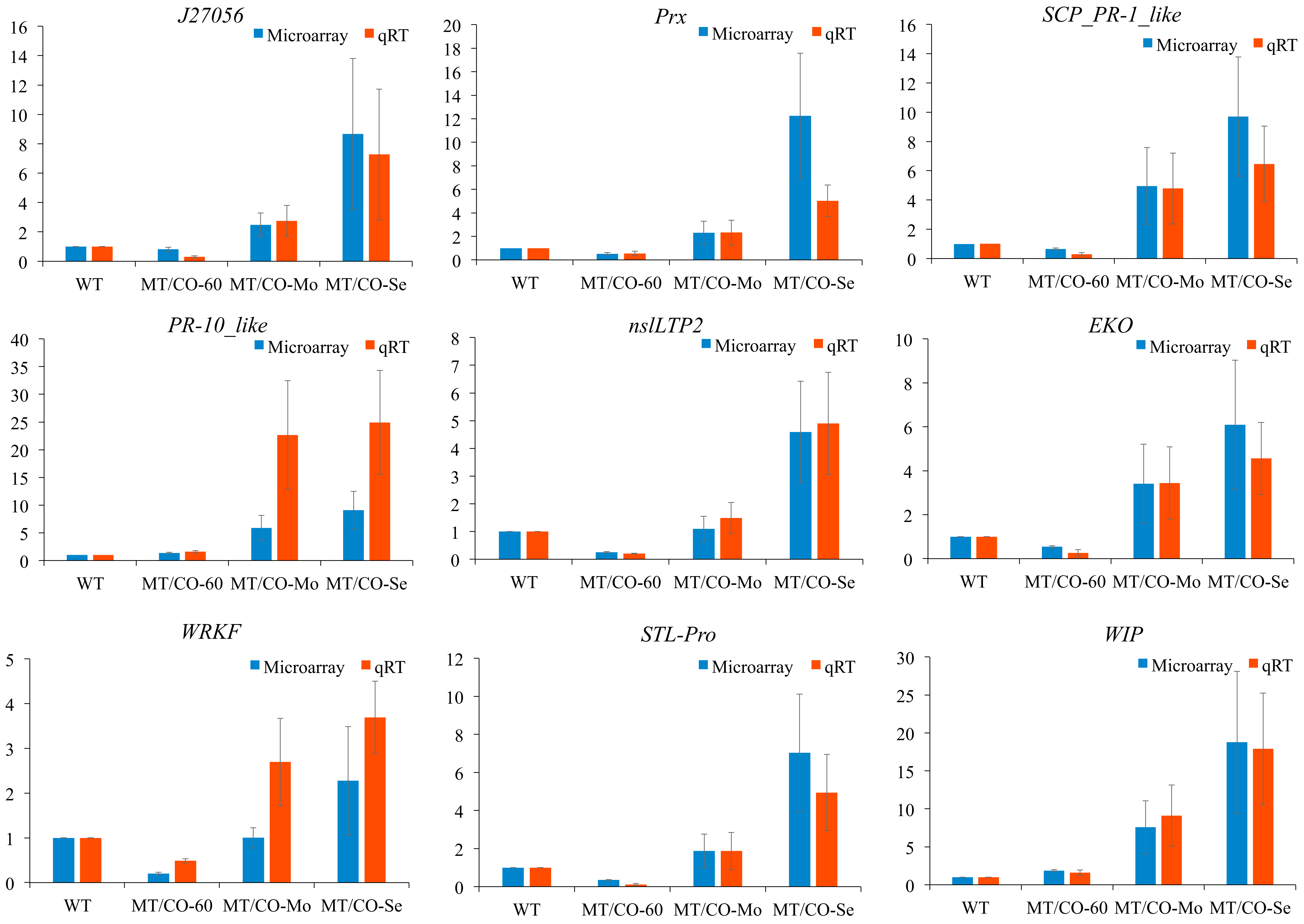

Fig. S6 Relative transcript levels of the selected genes related to lesion-mimic cell death phenotype in transgenic switchgrass. *Ubiquitin1* was used as the reference gene.

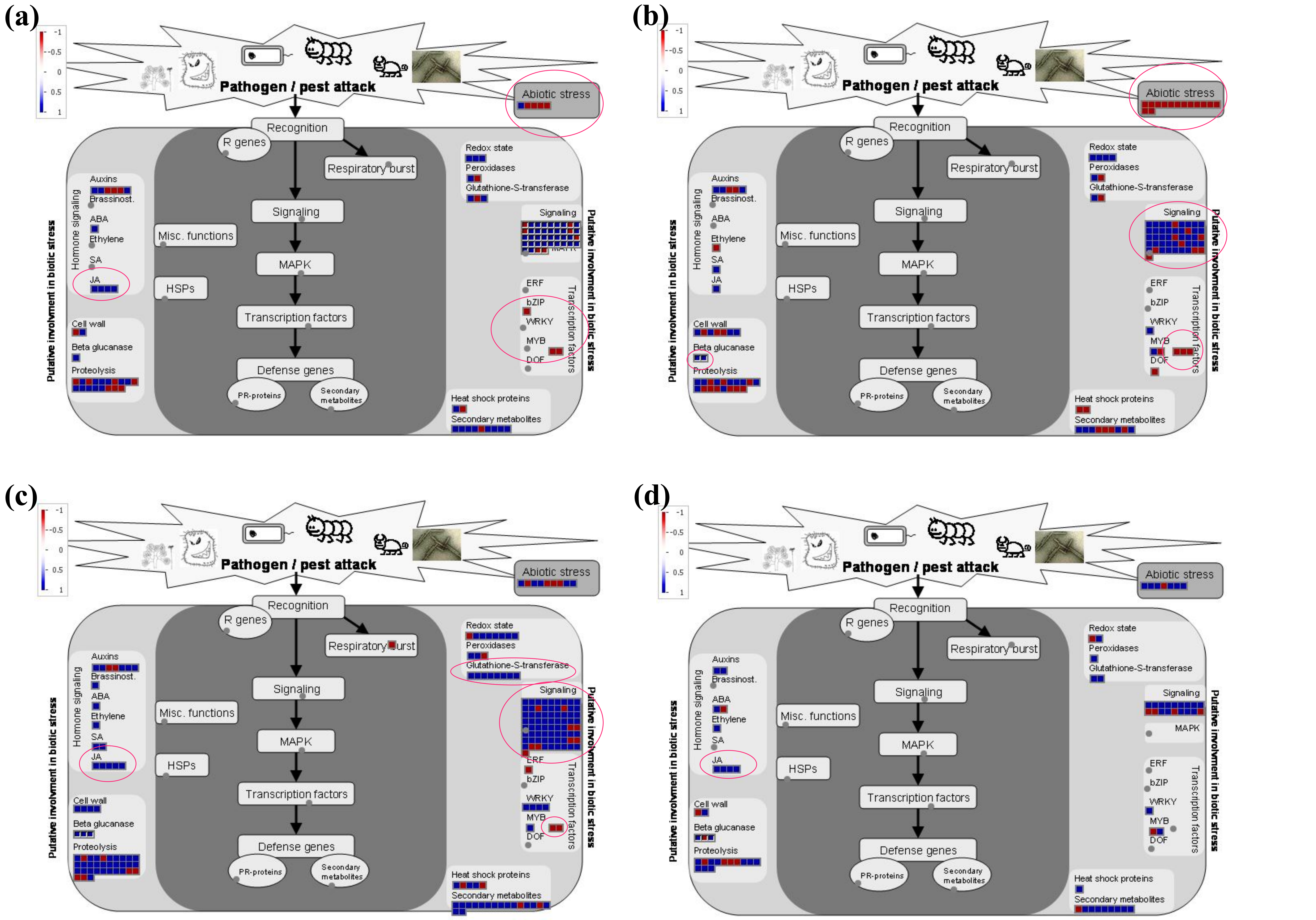

Supplementary Fig. S7 Significant DEGs related to biotic and abiotic stress pathways. DEGs from Exp/Ctrl group (a), Mo/60 group (b), Se/60 group (c), and Se/Mo group (d) were mapped onto MapMan 3.6.0RC1 using Log2-fold change ratios of pairwise comparison. Pink circle represents significant DEGs in each group.

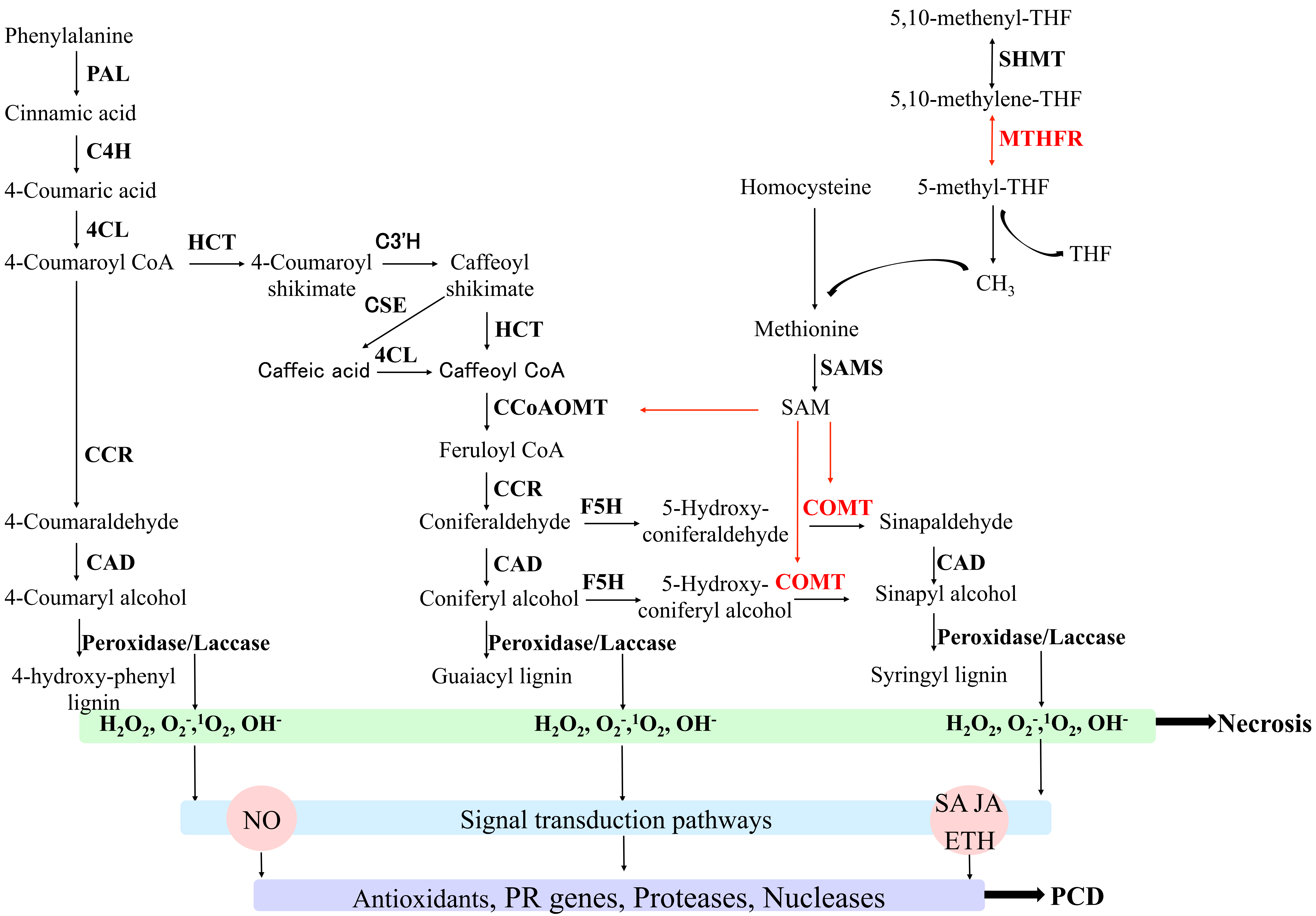

Supplementary Fig. S8 Possible connections between lignin biosynthesis pathway, C1 metabolism pathway and ROS-dependent cell death pathway. SA, salicylic acid; JA, jasmonic acid; ETH, ethylene; PR, pathogenesis-related; PCD, programmed cell death. Schematic was modified from Tang et al. (2014) and Van Breusegem & Dat (2006).

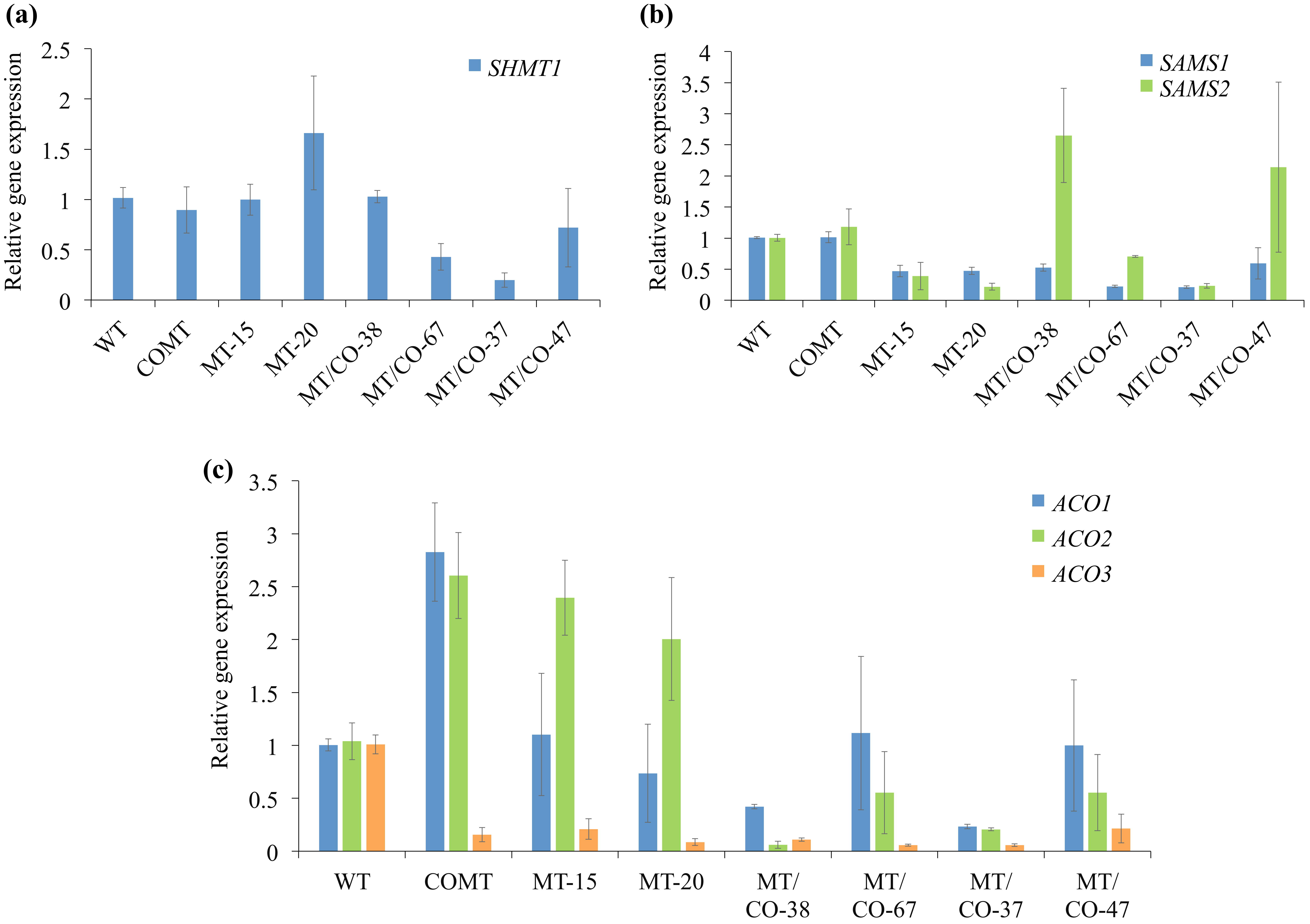

Supplementary Fig. S9 Relative transcript levels of *SHMT1* (a), *SAMS* (b) and *ACO* (c) in wild-type and transgenic switchgrass. Date shown are calculated by $2^{-\Delta\Delta Ct}$. method and normalized to wild-type plant. The accession number of each gene from phytozone is shown as follow: *SHMT1*, Pavir.Ia00634.1; *SAMS1*, Pavir.Ca00783.1 ; *SAMS2*, Pavir.Eb01268.1 ; *ACO1*, Pavir.J41046.1; *ACO2*, Pavir.J10323.1; *ACO3*, Pavir.J25986.1.

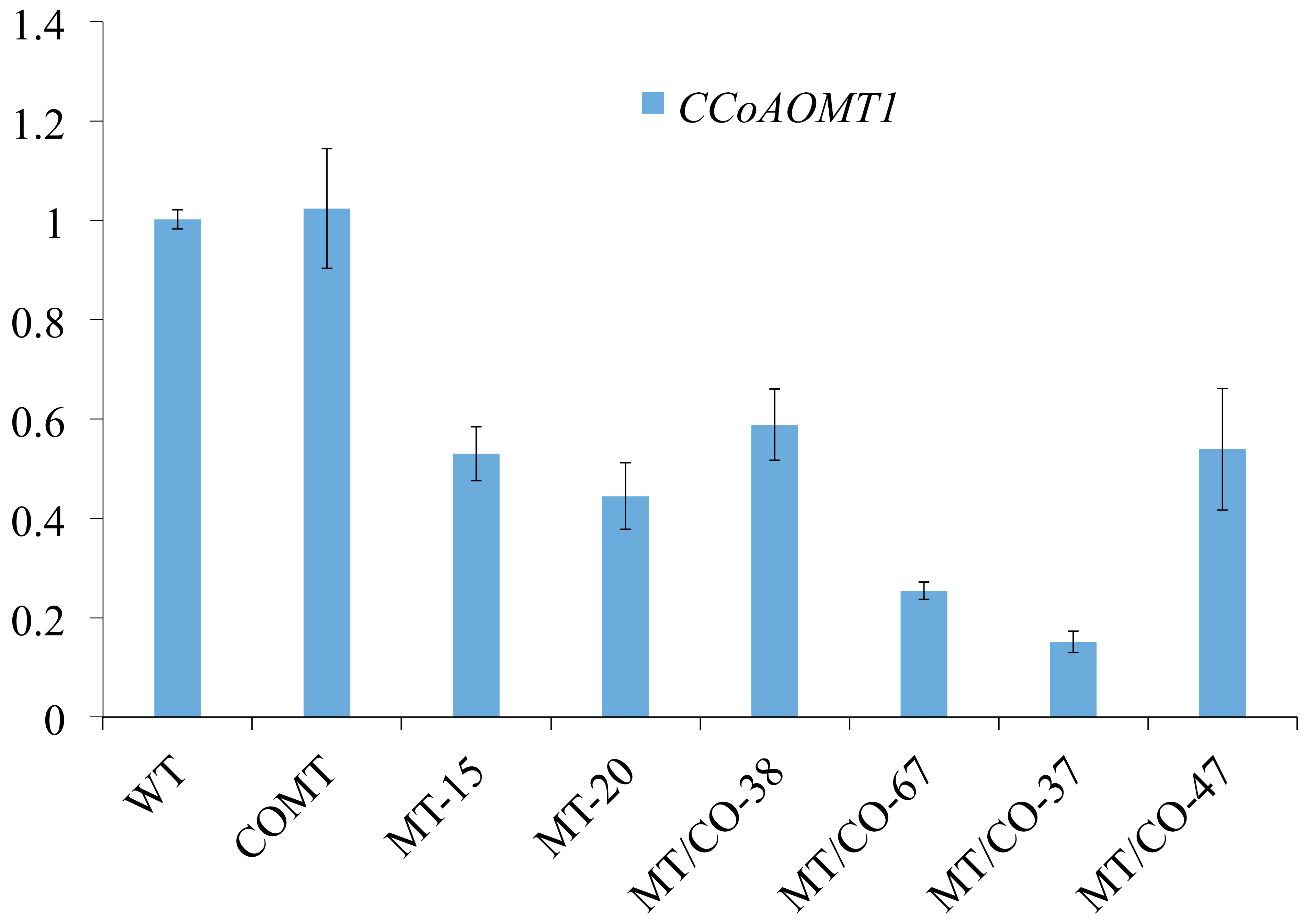

Supplementary Fig. S10 Relative transcript level of *CCoAOMT1* (Genebank accession: AB723825.1) in switchgrass..

Supplementary Table S1 Measurements of morphological characteristics of transgenic switchgrass plants

	Plant height (cm)	Leaf blade length (cm)	Leaf blade width (cm)	Leaf sheath length (cm)	Internode length (cm)	Internode diameter (mm)	Internod e number	Flowering day
WT	77.5 ± 2.1	34.6 ± 0.7	1.13 ± 0.03	12.3 ± 0.1	12.1 ± 1.0	3.6 ± 0.1	6-7	119 ± 1
COMT	72.8 ± 3.6	32.3 ± 1.3	1.03 ± 0.06	10.8 ± 0.4	12.0 ± 1.1	3.6 ± 0.2	5-6	113 ± 2*
MT-15	70.8 ± 2.2	28.8 ± 0.6	1.00 ± 0.04	11.1 ± 0.4	9.8 ± 0.3	3.6 ± 0.2	6	129 ± 2**
MT-20	70.5 ± 1.4	29.9 ± 0.5*	1.03 ± 0.05	11.2 ± 0.5	8.6 ± 1.6	3.5 ± 0.3	5-7	127 ± 2**
MT/CO-69	70.8 ± 1.4	32.6 ± 1.6	1.08 ± 0.03	11.9 ± 0.2	11.5 ± 1.0	3.4 ± 0.0	6-7	121 ± 3
MT/CO-70	79.8 ± 2.3	33.6 ± 1.0	1.03 ± 0.05	11.2 ± 0.6	12.5 ± 0.5	3.5 ± 0.1	5-6	119 ± 3
MT/CO-38	70.8 ± 1.3	32.5 ± 1.8	1.08 ± 0.06	12.1 ± 0.6	13.5 ± 1.4	3.7 ± 0.2	5-6	113 ± 2*
MT/CO-67	78.0 ± 4.0	34.6 ± 2.2	1.09 ± 0.03	12.4 ± 0.2	14.0 ± 0.8	3.6 ± 0.2	5-6	117 ± 1
MT/CO-37	52.0 ± 1.1**	29.0 ± 1.9*	0.96 ± 0.04*	10.3 ± 0.5**	7.0 ± 0.8**	3.2 ± 0.0	5-6	143 ± 4**
MT/CO-47	42.0 ± 1.2**	23.0 ± 2.9**	0.83 ± 0.03**	8.5 ± 0.1**	8.6 ± 0.6	3.0 ± 0.1*	5-6	146 ± 3**

Growth and development traits of switchgrass were measured at R1 stage. Four biological replications were measured for each line. Values are mean ±SE (n=4). One or two asterisks indicate significant difference of $P < 0.05$ or 0.01 by one way ANOVA, Dunnett' s test.

Supplementary Table S2 Differentially expressed genes (DEGs) of the four comparisons between severe and MT/CO-60 group, between moderate and MT/CO-60 group, between experimental and control group, and between severe and moderate group.

Comparisons	Total DEGs (fold change > 2.0)	Up regulated (fold change > 2.5)	Down regulated (fold change > 2.5)
Severe vs. MT/CO-60	2940	1693	321
Moderate vs. MT/CO-60	1363	409	287
Overlapped DEGs	593	201	126
Experimental vs. Control	1129	573	155
Severe vs. Moderate	1259	867	121

Experimental group means the combination of severe and moderate groups.

Control group means the combination of WT and MT/CO-60 groups.

Supplementary Table S7 Amino acid content in wild-type and transgenic plants

Amino acid	Young leaf					Old leaf				
	WT	COMT	MT-20	MT/CO-Mo	MT/CO-Se	WT	COMT	MT-20	MT/CO-Mo	MT/CO-Se
**Ala**	5.46 ± 1.42	26.86 ± 9.12	8.15 ± 1.25	13.71 ± 2.12	8.20 ± 1.85	3.48 ± 0.35	6.34 ± 2.21	6.56 ± 0.27**	4.33 ± 1.02	1.43 ± 0.08**
**Asp**	3.07 ± 0.63	7.18 ± 0.67*	4.66 ± 0.58	4.78 ± 0.89	6.21 ± 1.46	1.46 ± 0.02	3.55 ± 0.60*	4.65 ± 0.22*	2.83 ± 0.05**	2.78 ± 0.21**
**Gly**	1.29 ± 0.08	4.94 ± 0.65*	3.14 ± 0.53*	1.22 ± 0.02	1.30 ± 0.10	1.35 ± 0.10	1.24 ± 0.08	1.54 ± 0.21	1.26 ± 0.04	1.86 ± 0.08*
**Ile**	0.45 ± 0.01	1.59 ± 0.43	0.99 ± 0.02**	0.57 ± 0.01**	0.51 ± 0.01*	0.45 ± 0.01	0.76 ± 0.09*	0.64 ± 0.01	0.62 ± 0.03*	0.50 ± 0.01*
**Leu**	0.48 ± 0.02	2.91 ± 0.06**	1.21 ± 0.09**	0.59 ± 0.01*	0.53 ± 0.01	0.46 ± 0.01	0.89 ± 0.12*	0.69 ± 0.03	0.64 ± 0.03*	0.56 ± 0.01**
**Lys**	1.52 ± 0.06	4.27 ± 0.36**	2.33 ± 0.11**	1.49 ± 0.05	1.58 ± 0.06	1.36 ± 0.02	2.29 ± 0.26*	1.67 ± 0.06	1.44 ± 0.03	1.51 ± 0.02*
**Phe**	0.62 ± 0.05	1.63 ± 0.17**	1.15 ± 0.15*	0.70 ± 0.05	0.72 ± 0.05	0.46 ± 0.02	0.82 ± 0.08	0.75 ± 0.05*	0.68 ± 0.01**	0.83 ± 0.05*
**Pro**	0.61 ± 0.05	2.16 ± 0.74	0.98 ± 0.08*	0.82 ± 0.03*	0.75 ± 0.04	0.58 ± 0.01	0.98 ± 0.08**	1.04 ± 0.01**	0.90 ± 0.07*	3.10 ± 0.55*
**Tyr**	0.98 ± 0.02	10.31 ± 0.94**	3.81 ± 0.68	1.02 ± 0.03	1.06 ± 0.05	0.96 ± 0.01	2.01 ± 0.38	1.34 ± 0.07*	1.13 ± 0.03**	1.17 ± 0.12**
**Val**	0.72 ± 0.06	2.14 ± 0.60	1.15 ± 0.09*	0.98 ± 0.02*	0.84 ± 0.02	0.64 ± 0.01	1.00 ± 0.06	0.90 ± 0.01	0.94 ± 0.06*	0.68 ± 0.02

Ten amino acids were detected and analyzed by quantitative GC-TOF-MS method. Unit of the data was quantified to nmol/10mg DW (dry weight). Values are mean ±SE (n=3). Significance as determined by one Student' s t-*test*. *, $P < 0.05$; **, $P < 0.01$.

Supplementary Table S8 Altered abundance of metabolites in young and old leaves of wild-type and transgenic switchgrass.

Class	Compound	RT	Se/WT (Young leaf)		Mo/WT (Young leaf)		Se/WT (Old leaf)		Mo/WT (Old leaf)	
			**Fold change**	***P*-value**	**Fold change**	***P*-value**	**Fold change**	***P*-value**	**Fold change**	***P*-value**
**Organic acid**	Chlorogenic Acid	49.605	0.12**	0.000	0.20**	0.001	1.22	0.249	0.54	0.083
	Caffeic Acid	35.069	0.32*	0.032	0.91	0.681	0.48**	0.010	0.81	0.190
	Quinic acid	29.501	0.50	0.064	0.69	0.327	0.92	0.782	0.83	0.592
	Pipecolic Acid	18.196	0.68	0.556	1.47	0.631	16.41	0.076	58.83*	0.015
	Shikimic Acid	28.521	0.77	0.171	1.07	0.789	0.66	0.136	0.94	0.728
	Citric Acid	28.742	1.14	0.266	1.15	0.503	0.35**	0.001	1.37	0.054
	Maleic Acid	16.530	1.71	0.225	2.26	0.303	0.27*	0.022	1.41	0.155
	Succinic acid	16.806	2.41	0.060	2.22**	0.006	0.46	0.087	2.37*	0.011
	Propionic Acid, 2,3 Hydorxy	17.127	3.12*	0.022	2.93**	0.002	0.34**	0.000	1.98**	0.001
	Phosphoric Acid	15.574	3.46*	0.019	1.92	0.212	1.43	0.120	1.33	0.177
**Amino acid**	L-Alanine	10.995	2.98*	0.013	3.71**	0.005	0.38*	0.032	1.45	0.126
	L-Aspartic acid	22.033	2.39	0.150	2.01	0.242	3.32**	0.007	3.10**	0.000
	L-Asparagine	25.588	1.43	0.691	1.17	0.848	89.08*	0.020	2.56	0.383
	L-Glutamic acid	24.422	3.33	0.087	3.56	0.097	2.62*	0.039	2.49**	0.006
	Glycine	16.574	1.32	0.372	0.94	0.786	2.08*	0.047	1.05	0.845
	L-Proline	16.392	0.57	0.575	1.28	0.700	39.95	0.067	2.94	0.237
	Pyroglutamic acid	22.196	1.60	0.380	1.75	0.296	4.20*	0.014	2.43**	0.001
	L-Serine	17.944	2.39	0.313	2.26	0.205	28.08*	0.007	5.86*	0.023
	L-Threonine	18.602	2.15	0.055	4.43*	0.019	3.04**	0.008	2.15**	0.000
**Sugar**	D-(+)-Glucose	30.269	0.82	0.419	1.39	0.402	57.11**	0.002	8.23	0.133
	1-Methyl-alpha-D-glucopyranoside	29.299	0.83	0.095	1.37**	0.008	0.64	0.077	1.36	0.056
	D-(+)-Galactose	30.146	0.92	0.856	1.69	0.153	1.62*	0.017	1.16	0.548
	Sucrose	43.010	1.25	0.388	1.28	0.283	2.89**	0.001	1.28	0.306
	galactosyl glycerol	38.423	1.41*	0.015	2.24*	0.016	0.45*	0.043	1.69*	0.043
	D-(-)-Fructose	29.745	1.42	0.192	1.53	0.245	11.82**	0.001	2.87	0.081
**Fatty acid methyl esters**	Methyl Tricosanoate	53.978	0.12*	0.039	0.29	0.140	0.37	0.196	0.60	0.247
	Methyl hexadecanoate	48.007	0.35	0.082	0.64	0.196	0.45	0.125	0.89	0.634
	Methyl Tetracosanoate	45.231	0.40	0.225	0.64	0.177	0.40*	0.030	0.83	0.248
	Methyl Octadecanoate	35.544	1.21	0.085	1.48	0.135	1.31	0.267	1.28	0.125
	Methyl linoleate	34.989	1.23*	0.013	1.94**	0.007	2.63*	0.045	1.85*	0.012
	Methyl hexadecanoate	31.727	2.03*	0.034	2.64	0.074	5.42	0.108	1.76*	0.032
**Others**	alpha-glycerophosphate ester	28.044	0.23*	0.017	0.68*	0.020	1.07	0.663	0.93	0.764
	4-Hydroxypyridine	9.957	0.24**	0.002	0.46**	0.005	0.40	0.110	0.42	0.055
	beta-glycerophosphate	27.235	0.28*	0.015	0.70	0.073	1.26	0.264	1.04	0.617
	Umbelliferone	30.178	0.52	0.058	0.86*	0.040	0.82*	0.014	0.88**	0.009
	Trisiloxane, Octamethyl-	10.496	0.53	0.805	0.47*	0.020	0.76	0.447	0.74	0.432
	Glycerol	16.120	0.84	0.288	1.31**	0.020	0.72*	0.023	1.21*	0.036
	Ethanol Amine	16.033	1.10**	0.003	1.53*	0.021	0.85	0.506	1.22	0.274
	Myo-Inositol	34.055	1.95	0.066	1.51	0.169	0.36**	0.001	1.11	0.578

Fold change values represent the peak area of each compound after normalization to the mean peak area of wild-type. Values are mean ±SE (n=3). Significance as determined by Student's t-test. *, $P < 0.05$; **, $P < 0.01$. RT, retention time.

Table S9 Sequences of primers used in PCR and qRT-PCR experiments

Primer name	Forward (5'-3')	Reverse (5'-3')
MTHFR-RNAi	caccGCGCACGGCCCGTCCTTCT	ATTTGACGACAGTCATTCACA
MTHFR-q	GTATGGCCAAATCCGACAACT	ATGGGAGGAAATGGATAGATAACA
COMT-q	TACGAGAGGGAGTTCGAGGAG	CATGCGTTGGCGTAGATGTAG
bar	CCGTACCGAGCCGCAGGAAC	CAAATCTCGGTGACGGGCAGGAC
hyg	AAGGAATCGGTCAATACACTACATGG	AAGACCAATGCGGAGCATATACG
PvUbiquitin	TTCGTGGTGGCCAGTAAG	AGAGACCAGAAGACCCAGGTACAG
SHMT1	AGCAAGCAAAGATGCCAAGA	AAGATGCCATTCCACTTTCG
SAMS1	TCACTTTGGAAGGGATGACC	ATCACAACACTGGAGCCACA
SAMS2	GCTGCTGATGTTACCTGCAA	CCGAAGCCTTCTTGTTCATC
ACO1	AGCAGGCATGATCTGAACCT	CAGGGCACACTACCACACAT
ACO2	CGCTTTTTGCTTGTTGTTGA	AGCTGTACTTGACGGGATGC
ACO3	TCGGCAGCTTTACCTGAATAA	CGACACTCGACACAGACGTAA
STL-Pro	AGATCGGCGGTCCATATACA	AACCAGTAGCCCAGTTCACC
nsLTP2	GCCTTCGAGTTTACCCAACA	CCATTTATGAACCAGCAGCA
WIP	GATGACGACTACTCCATCACCA	CGCCATGTGGGGTTACATA
EKO	CCACATGGCTGATGGTACTG	CAGAGATGGTGTTCGGTTGA
WRKF	GTGGCCAGATATCCACATGC	CAGAACGAGGCTGTTCATCA
Prx	CCATCTCGCTTTCTTGATCC	CTCCATGCCTTCTTGTAGCC
SCP_PR1-like	ATCTAGCAATGGCGGAGAAG	CGACTCCGATCGAGAGATTA
PR10-like	AGCACCGAGAGCCAAGTC	GCCGAAGAAAAGAAAACACA
J27056	CGCCCTAAGTTACTCGTTGG	TGCTATGGCGTCATCTTCAT
CCoAOMT1	CCGTCTTTCTTTTTTTGGCTCTT	GCATGAAAATGATGACAGTTTCCA
