# Supplementary material for: Simultaneous Downregulation of MTHFR and COMT in Switchgrass Affects Plant Performance and Induces Lesion-Mimic Cell Death
Source: Front Plant Sci. 2017 Jun 20;8:982. doi: 10.3389/fpls.2017.00982 (PMC5476930; doi:10.3389/fpls.2017.00982)
Supplement: Supplementary file 2 [file Presentation_2.PDF]

Table S3. List of commonly up-regulated genes with more than 2.5 fold change between Se/60 and Mo/60 comparisons

Genes not corrolated with lesion development (Se/Mo<1) were ruled out and highlighted in red. P, Bonferroni-corrected P value.

probeset_id	PviUT sequence ID	CDD annotation	Panther annotation	Arabidopsis hit	Rice hit	Ratio(Se/60)	P	Ratio(Mo/60	P	Ratio(Se/Mo)	P
AP13ITG38511_s_at	AP13ISTG38511	PLN02555/limonoid gl	UDP-GLUCOSYLTRANS	/	LOC_Os04g12970.1|13104.m01188|protein/1E-82//	113.02	0	5.31	4.35E-62	21.29	0
AP13ITG67309_s_at	AP13ISTG67309	pfam00450/Serine car	SERINE CARBOXYPEPT	/	LOC_Os10g01134.1|13110.m00014|protein/0//	53.79	0	14.05	0	3.83	4.60E-20
KanlowCTG06220_s_at						46.29	0	5.95	9.34E-293	7.78	1.06E-54
KanlowCTG09824_s_at	KanlCTG09824	COG0534/Na+-driven	SUBFAMILY NOT NAM	/	LOC_Os03g37640.1|13103.m04087|protein/8E-53//	42.52	0	8.59	0	4.95	1.42E-17
AP13ITG65101_at	AP13ISTG65101	pfam02956/TT viral or	/	/	///	33.56	0	3.92	3.30E-97	8.56	3.96E-54
AP13CTG58961_at	AP13CTG58961	PRK12323/DNA polym	/	/	LOC_Os01g58140.1|13101.m06174|protein/0.0000000002//	30.22	0	3.66	8.40E-172	8.25	5.91E-62
AP13ITG71440_at	AP13ISTG71440	PLN02183/ferulate 5-h	SUBFAMILY NOT NAM	AT2G30750/Limonene	LOC_Os06g43384.1|13106.m04539|protein/1E-164//	22.21	0	5.47	0	4.06	3.37E-06
AP13CTG09437_at	AP13CTG09437	cd00693/Horseradish	/	AT5G05340/Phenylala	LOC_Os07g48040.1|13107.m05132|protein/2E-108//	21.76	0	4.43	1.73E-47	4.91	2.39E-15
AP13CTG26173_at	AP13CTG26173	pfam00173/Cytochron	FAMILY NOT NAMED/	/	LOC_Os02g43360.1|13102.m04815|protein/1E-41//	19.26	0	11.5	0	1.67	0.188811429
AP13CTG15831_s_at	AP13CTG15831	cd04852/Peptidase S8	SUBTILISIN-LIKE PROT	/	LOC_Os01g58280.1|13101.m06185|protein/0//	17.15	0	5.28	0	3.25	3.44E-05
AP13ITG42000_at	AP13ISTG42000	pfam00314/Thaumatir	/	/	LOC_Os12g43430.1|13112.m04577|protein/1E-68//	15.09	3.28E-130	11.13	3.09E-68	1.36	0.329096432
AP13ITG58844_at	AP13ISTG58844	PLN02987/cytochrome	SUBFAMILY NOT NAM	AT4G19230/Caroteno	LOC_Os07g33440.1|13107.m03397|protein/9E-175//	14.2	0	3.46	0	4.11	2.51E-09
AP13ITG59977_at	AP13ISTG59977	cd03185/GST_C family	GLUTATHIONE S-TRAN	/	LOC_Os10g38470.1|13110.m03503|protein/1E-79//	14.09	0	3.77	0	3.74	1.03E-06
KanlowCTG07131_s_at	KanlCTG07131	cd05381/SCP_PR-1_lik	CHLORIDE CHANNEL C	/	LOC_Os12g25200.1|13112.m02555|protein/2E-148//	14.05	0	5.34	0	2.63	0.009683484
KanlowSLT48175_s_at	KanlSGLT48175	//	/	/	LOC_Os07g18570.1|13107.m01946|protein/0.0000000000005//	13.92	0	4.77	8.28E-298	2.92	9.48E-12
AP13ITG52984_at	AP13ISTG52984	cd01959/nsLTP2: Non-	/	/	LOC_Os09g23370.2|13109.m02207|protein/4E-64//	13.14	0	4.28	0	3.07	1.22E-05
AP13CTG27003_at	AP13CTG27003	PLN02998/hydrolase, I	GLYCOSYL HYDROLASI	/	LOC_Os09g33680.1|13109.m03354|protein/0/Cyanoamino acid metabolism; Sta	12.73	0	2.57	5.18E-45	4.96	3.29E-68
AP13ITG63167_at	AP13ISTG63167	PLN02655/ent-kauren	SUBFAMILY NOT NAM	AT5G25900/Diterpen	LOC_Os06g37330.1|13106.m03864|protein/0/Diterpenoid biosynthesis; Metabo	12.62	0	6.25	0	2.02	0.094736978
AP13CTG58860_at	AP13CTG58860	cd05381/SCP_PR-1_lik	CYSTEINE-RICH SECRE	AT2G14610/Plant-pat	LOC_Os07g03710.1|13107.m00310|protein/6E-59//	12.48	0	7.51	0	1.66	0.280157866
AlamoCTG14398_s_at	AlamCTG14398	//	OS09G0517200 PROT	/Plant-pathogen inter	LOC_Os09g34160.1|13109.m03430|protein/1E-88//	12.44	0	3.06	3.49E-26	4.07	7.71E-17
OTHSWCTG20397_s_at	OthsCTG20397	pfam03060/2-nitropro	BLL5091 PROTEIN/0.0	/	LOC_Os08g37874.1|13108.m03998|protein/1E-96//	12.39	0	2.55	9.05E-39	4.85	1.07E-68
AP13ITG59511_at	AP13ISTG59511	//	/	/	///	12.26	0	3	3.98E-94	4.08	7.41E-17
OTHSWCTG15870_at	OthsCTG15870	pfam03106/WRKY DN	/	/	LOC_Os11g29870.1|13111.m02887|protein/0.0000000000000003//	12.21	0	4.88	8.61E-178	2.5	6.29E-09
AP13CTG24721_at	AP13CTG24721	PLN02448/UDP-glycos	UDP-GLUCOSYLTRANS	AT3G50740/Phenylpr	LOC_Os04g46990.1|13104.m04753|protein/6E-148/Zeatin biosynthesis/3E-147	11.77	0	4.78	0	2.46	0.000637203
AP13ITG62483_at	AP13ISTG62483	pfam00967/Barwin far	WOUND-INDUCED PR	/	LOC_Os11g37950.1|13111.m03723|protein/7E-48//	11.57	0	4.09	0	2.83	0.000561435
KanlowCTG08567_s_at	KanlCTG08567	pfam00230/Major intr	SUBFAMILY NOT NAM	/	LOC_Os02g44630.2|13102.m04992|protein/4E-135//	11.34	0	4.79	0	2.37	1.84E-38
AP13ITG41899_s_at	AP13ISTG41899	//	/	/	LOC_Os05g03750.1|13105.m00366|protein/1E-23//	11.32	1.09E-293	8.69	9.46E-164	1.3	0.268660342
AP13CTG34009_s_at	AP13CTG34009	PLN02849/glycosyl hy	GLYCOSYL HYDROLASI	/	LOC_Os05g30350.2|13105.m03099|protein/5E-179/Cyanoamino acid metabolis	11.24	1.66E-163	3.97	2.77E-15	2.83	2.94E-12
AP13CTG14744_s_at	AP13CTG14744	//	/	/	LOC_Os12g36110.1|13112.m03703|protein/1E-59//	10.15	6.01E-304	6.67	5.58E-118	1.52	0.253560931
KanlowCTG39787RC_s_	KanlCTG39787-RC	PHA03247/large tegun	FAMILY NOT NAMED/	/	LOC_Os03g19650.3|13103.m02355|protein/1E-150//	9.77	0	3.89	1.72E-35	2.51	0.000956704
AP13ITG67309_at	AP13ISTG67309	pfam00450/Serine car	SERINE CARBOXYPEPT	/	LOC_Os10g01134.1|13110.m00014|protein/0//	9.75	0	3.44	0	2.84	7.87E-16
AP13CTG27420_at	AP13CTG27420	smart00774/DNA bind	/	/	LOC_Os05g25770.1|13105.m02640|protein/4E-43//	9.51	0	3.65	1.10E-136	2.6	0.001345997
AP13ITG70591-RC_at						9.09	0	7.01	1.54E-220	1.3	0.324847511
AP13CTG30873_at	AP13CTG30873	pfam00635/MSP (Maj	OS04G0369000 PROT	/	LOC_Os11g17380.1|13111.m01762|protein/1E-39//	8.99	0	16.29	0	0.55	0.12814943
AP13CTG27562_s_at	AP13CTG27562	pfam00083/Sugar (and	SUBFAMILY NOT NAM	/	LOC_Os03g11900.1|13103.m01345|protein/0//	8.95	0	4.93	6.47E-291	1.81	0.003978238
AP13CTG48706_at	AP13CTG48706	cd00180/Catalytic dor	OS11G0212300 PROT	/	LOC_Os11g39450.1|13111.m03897|protein/6E-61//	8.89	0	15.29	0	0.58	0.175961798
AP13CTG14744_at	AP13CTG14744	//	/	/	LOC_Os12g36110.1|13112.m03703|protein/1E-59//	8.75	0	3.22	0	2.72	0.000209459
KanlowCTG47289_s_at	KanlCTG47289	PRK07538/hypothetica	MONOXYGENASE/3.6	/	LOC_Os03g05900.2|13103.m00642|protein/7E-75//	8.62	0	2.86	8.47E-61	3.01	4.56E-18
KanlowCTG12598_s_at	KanlCTG12598	smart00516/Domain ir	SEC14 CYTOSOLIC FAC	/	LOC_Os01g70210.1|13101.m07621|protein/2E-99//	8.61	0	7.86	0	1.09	0.786560798
AP13CTG22556_at	AP13CTG22556	pfam03080/Arabidops	/	/	LOC_Os07g38590.1|13107.m03991|protein/4E-102//	8.57	2.25E-287	10.76	0	0.8	0.569906754
AP13CTG09569_s_at	AP13CTG09569	PLN00141/Tic62-NAD(	SUBFAMILY NOT NAM	/	LOC_Os05g01970.5|13105.m00143|protein/5E-123//	8.49	0	3.43	1.71E-58	2.47	7.50E-10
AP13CTG02814_s_at	AP13CTG02814	PRK00277/ATP-depen	ATP-DEPENDENT CLP	/	LOC_Os03g19510.1|13103.m02334|protein/4E-119//	8.33	0	2.72	1.11E-203	3.06	1.03E-22
AP13CTG27808_at	AP13CTG27808	pfam00450/Serine car	SERINE CARBOXYPEPT	AT5G09640/Phenylpr	LOC_Os10g01110.1|13110.m00012|protein/2E-171//	8.2	1.03E-105	7.94	2.40E-98	1.03	0.86644618
AP13ITG66548_s_at	AP13ISTG66548	PRK05557/3-ketoacyl-	FAMILY NOT NAMED/	AT4G13180/Fatty acid	LOC_Os10g31780.1|13110.m02768|protein/6E-109/Fatty acid biosynthesis; Bios	8.17	9.85E-134	7.84	6.45E-122	1.04	0.911779426
KanlowCTG38097_at	KanlCTG38097	PHA02988/hypothetic	SUBFAMILY NOT NAM	/	LOC_Os11g17380.1|13111.m01762|protein/0.000003//	8.04	0	3.06	8.76E-46	2.63	4.74E-17
AP13CTG29478_s_at	AP13CTG29478	PLN02365/2-oxoglutar	OXIDOREDUCTASE, 2(	/	LOC_Os04g39980.1|13104.m03879|protein/9E-127//	8.02	0	16.24	0	0.49	6.39E-05
KanlowCTG26347_at	KanlCTG26347	pfam03492/SAM depe	/	/	LOC_Os06g13350.1|13106.m01470|protein/2E-110//	7.65	0	5.73	1.26E-270	1.34	0.45350243
AP13ITG70166_s_at	AP13ISTG70166	pfam00854/POT family	NITRATE TRANSPORTI	/	LOC_Os12g44110.2|13112.m04657|protein/6E-150//	7.61	0	4.48	1.19E-191	1.7	0.014922825
OTHSWCTG04721_s_at	OthsCTG04721	//	/	/	///	7.57	2.57E-112	4.47	1.01E-32	1.69	0.113344765
AP13ITG64138_at	AP13ISTG64138	PRK05648/DNA polym	/	/	LOC_Os07g38150.1|13107.m03939|protein/1E-38//	7.48	3.72E-144	3.8	2.00E-28	1.97	0.000833787
AP13ITG59134_at	AP13ISTG59134	pfam02201/SWIB/MDI	FAMILY NOT NAMED/	/	LOC_Os12g32280.1|13112.m03293|protein/4E-32//	7.26	0	2.53	8.52E-62	2.87	3.85E-18
AP13ITG73548_s_at	AP13ISTG73548	//	FAMILY NOT NAMED/	/	LOC_Os12g32280.1|13112.m03293|protein/8E-32//	7.25	0	2.94	0	2.47	1.55E-10
AP13CTG12196_s_at	AP13CTG12196	//	SUCROSE TRANSPORT	/	LOC_Os02g36700.1|13102.m04004|protein/0//	7.19	0	10.54	0	0.68	0.012271272
AP13CTG12078_s_at	AP13CTG12078	PRK00277/ATP-depen	ATP-DEPENDENT CLP	/	LOC_Os03g19510.1|13103.m02334|protein/9E-119//	7.16	0	2.73	1.00E-186	2.63	7.75E-14
AP13CTG09699_s_at	AP13CTG09699	PTZ00146/fibrillarin; P	UNCHARACTERIZED/5	/	LOC_Os04g39010.1|13104.m03765|protein/3E-20//	7.08	5.23E-188	3.09	8.47E-24	2.29	0.000329465
AP13CTG24971_at	AP13CTG24971	pfam05955/Equine he	NUCLEOREDOXIN/0.0	/	LOC_Os08g31250.1|13108.m03235|protein/2E-57//	6.97	0	4.07	9.78E-113	1.71	0.09008252
AP13CTG22084_s_at	AP13CTG22084	pfam03134/TB2/DP1,	HVA22-LIKE PROTEIN	/	LOC_Os11g30500.1|13111.m02953|protein/2E-51//	6.59	0	2.57	0	2.56	7.17E-134
AP13ITG56341_s_at	AP13ISTG56341	PRK11325/scaffold prc	IRON-SULFUR CLUSTE	/	LOC_Os01g47340.1|13101.m04879|protein/1E-63//	6.58	0	3.61	3.24E-89	1.83	8.58E-06
AP13ITG73622RC_at	AP13ISTG73622-RC	cd00051/EF-hand, calc	CALCIUM-BINDING EF	AT3G47480/Plant-pat	LOC_Os09g24580.1|13109.m02333|protein/0.00000008/Plant-pathogen interac	6.56	0	3.42	3.68E-82	1.92	0.036010417
AP13CTG19074_at	AP13CTG19074	smart00219/Tyrosine I	SUBFAMILY NOT NAM	/	LOC_Os05g40270.7|13105.m04254|protein/0//	6.48	2.37E-287	5.07	6.23E-159	1.28	0.339665239
OTHSWSLT36401_s_at	OthsSGLT36401	TIGR00957/multi drug	SUBFAMILY NOT NAM	/	LOC_Os04g49890.1|13104.m05101|protein/4E-112//	6.4	0	2.96	4.00E-103	2.16	1.52E-24
AP13ITG62754_s_at	AP13ISTG62754	COG0412/Dienelacton	ENDO-1,3-1,4-BETA-D	/	LOC_Os05g33110.1|13105.m03417|protein/1E-113//	6.39	0	3.72	6.50E-102	1.72	1.39E-12
AP13ITG67117_at	AP13ISTG67117	//	COPPER TRANSPORT I	/	LOC_Os06g35060.1|13106.m03620|protein/2E-37//	6.34	0	3.23	6.08E-294	1.96	0.036108972
AP13ITG55801_at	AP13ISTG55801	cd00693/Horseradish	/	AT4G11290/Phenylala	LOC_Os05g06970.1|13105.m00752|protein/6E-133//	6.25	2.12E-232	6.44	2.94E-249	0.97	0.95294716
KanlowCTG14963_s_at	KanlCTG14963	TIGR00887/phosphate	SUBFAMILY NOT NAM	/	LOC_Os03g05620.1|13103.m00607|protein/8E-123//	6.21	1.17E-194	4.62	6.53E-95	1.34	0.417807256
AP13ITG56240_at	AP13ISTG56240	//	ALCOHOL DEHYDROG	AT5G42250/Glycolysis	LOC_Os03g09020.1|13103.m30166|protein/7E-121/Glycolysis / Gluconeogenesi	6.09	0	3.53	2.04E-172	1.73	0.19466099
KanlowCTG05742_s_at	KanlCTG05742	//	/	AT5G57580/Phosphat	LOC_Os01g04280.1|13101.m00386|protein/5E-172//	6.06	1.50E-267	3.43	7.34E-63	1.77	0.046787066

AP13ITG60086_at	AP13ISTG60086	smart00774/DNA bind	/	/	LOC_Os07g48260.1|13107.m05157|protein/2E-43//	6.05	0	3.15	0	1.92	0.034281629
AP13CTG06246-2_s_at						6.04	7.81E-72	9.07	7.41E-181	0.67	0.073194301
KanlowCTG47117_s_at	KanlCTG47117	PLN02183/ferulate 5-h	SUBFAMILY NOT NAM	AT5G25140/Limonene	LOC_Os04g10160.1|13104.m00909|protein/1E-88//	6.01	0	4.08	1.56E-197	1.47	0.275708038
KanlowCTG47529_s_at	KanlCTG47529	PLN02183/ferulate 5-h	OS07G0291800 PROT	AT3G26300/Limonene	LOC_Os04g10160.1|13104.m00909|protein/5E-43//	5.94	8.85E-101	4.04	3.37E-39	1.47	0.309248821
KanlowCTG01327_s_at	KanlCTG01327	PLN02609/catalase/0	FAMILY NOT NAMED/	AT1G20630/Tryptopha	LOC_Os02g02400.1|13102.m00175|protein/0/Tryptophan metabolism; Metabol	5.91	2.75E-261	8.35	0	0.71	0.404073106
OTHSWCTG19595_at	OthsCTG19595	PLN00113/leucine-rich	OS10G0120300 PROT	/	LOC_Os01g41770.1|13101.m04285|protein/1E-60//	5.89	0	5.67	0	1.04	0.870435331
KanlowSLT53174_s_at	KanlSGLT53174	//	/	/	///	5.8	5.03E-149	4.26	7.23E-70	1.36	0.22376577
AP13CTG03745_at	AP13CTG03745	PLN02790/transketola	TRANSKETOLASE/0	AT2G45290/Pentose p	LOC_Os04g19740.1|13104.m01814|protein/0/Pentose phosphate pathway; Carl	5.78	0	5.64	0	1.02	0.933245522
KanlowCTG38102_s_at	KanlCTG38102	cd03250/Domain 1 of	SUBFAMILY NOT NAM	/	LOC_Os04g49890.1|13104.m05101|protein/4E-115//	5.71	7.50E-183	2.73	3.15E-26	2.09	5.27E-06
KanlowCTG14163_at	KanlCTG14163	PLN02448/UDP-glycos	UDP-GLUCOSYLTRANS	AT3G50740/Phenylpro	LOC_Os04g46990.1|13104.m04753|protein/2E-133/Zeatin biosynthesis/9E-133	5.7	0	3.34	1.91E-197	1.71	0.119004511
AP13ITG71141_at	AP13ISTG71141	cd00180/Catalytic dor	SUBFAMILY NOT NAM	/	LOC_Os12g26940.1|13112.m02739|protein/8E-58//	5.69	0	8.08	0	0.7	0.194517513
AP13ITG52579_x_at	AP13ISTG52579	pfam00182/Chitinase	GB DEF: CHITINASE (F	AT3G12500/Amino su	LOC_Os03g04060.1|13103.m00413|protein/2E-46/Amino sugar and nucleotide s	5.67	2.13E-86	9.78	1.73E-299	0.58	0.05340598
AP13CTG27594_at	AP13CTG27594	TIGR01377/sarcosine	PEROXISOMAL SARCO	/	LOC_Os09g32290.2|13109.m07548|protein/9E-173//	5.66	4.46E-112	7.04	5.96E-187	0.8	0.399642533
VS16ITG16394_at	VS16ISTG16394	//	/	/	///	5.65	0	3.15	2.00E-141	1.79	0.003861523
AP13ITG64260_s_at	AP13ISTG64260	//	/	/	LOC_Os01g24340.1|13101.m02574|protein/0.0000000000008//	5.6	2.14E-191	4.76	5.50E-129	1.17	0.544613796
KanlowCTG01918_at	KanlCTG01918	//	MALIC ENZYME/0	AT5G25880/Pyruvate	LOC_Os05g09440.1|13105.m01052|protein/0/Pyruvate metabolism; Carbon fixa	5.58	2.23E-258	2.93	2.66E-47	1.91	4.20E-05
AP13ITG46253_s_at						5.57	0	4.35	0	1.28	0.639445479
AP13ITG62309_at	AP13ISTG62309	cd06660/Aldo-keto re	ALDO/KETO REDUCTA	/	LOC_Os04g26910.1|13104.m02546|protein/9E-162//	5.55	1.84E-114	5.21	2.32E-98	1.06	0.849910929
AP13ITG62725_at	AP13ISTG62725	pfam00407/Pathogene	/	/	LOC_Os03g18850.1|13103.m02258|protein/2E-74//	5.49	0	4.31	0	1.27	0.534964941
KanlowCTG21384_x_at	KanlCTG21384	PLN02183/ferulate 5-h	SUBFAMILY NOT NAM	AT3G26160/Limonene	LOC_Os04g10160.1|13104.m00909|protein/1E-59//	5.48	2.87E-78	5.86	7.69E-92	0.93	0.843253222
AP13ITG44916-RC_s_at						5.47	1.72E-129	4.33	1.08E-72	1.26	0.526193977
AP13ITG44757_s_at	AP13ISTG44757	cd04666/Members of	FAMILY NOT NAMED/	/	LOC_Os11g32750.1|13111.m03193|protein/1E-97//	5.43	0	3.67	5.43E-182	1.48	0.078980232
AP13CTG10204_at	AP13CTG10204	//	/	/	LOC_Os12g37610.1|13112.m03870|protein/6E-61//	5.34	7.59E-76	3.52	1.13E-26	1.52	0.022680236
AP13CTG24955_x_at	AP13CTG24955	//	/	/	///	5.29	1.04E-123	3.54	2.15E-44	1.5	0.087931132
AP13ITG65600-RC_at						5.26	0	5.41	0	0.97	0.841632496
OTHSWCTG12073_s_at	OthsCTG12073	pfam00407/Pathogene	/	/	LOC_Os12g36830.1|13112.m03777|protein/9E-26//	5.18	5.47E-24	6.6	1.15E-41	0.79	0.324586126
OTHSWCTG13770_s_at	OthsCTG13770	//	/	/	///	5.17	0	2.55	2.84E-79	2.02	5.52E-06
AP13ITG44735_s_at	AP13ISTG44735	smart00219/Tyrosine I	SUBFAMILY NOT NAM	/	LOC_Os03g24930.1|13103.m02938|protein/0//	5.16	1.85E-112	5.46	1.21E-128	0.95	0.846019739
AP13ITG54363_at	AP13ISTG54363	pfam04788/Protein of	/	/	LOC_Os03g07120.1|13103.m00787|protein/2E-129//	5.08	6.42E-84	2.6	3.01E-14	1.96	1.31E-09
AP13ITG76492_at	AP13ISTG76492	//	ALPHA-AMYLASE/5.6E	AT4G25000/Starch an	LOC_Os06g49970.2|13106.m05364|protein/0/Starch and sucrose metabolism; N	5.07	0	6.48	1.00E-105	0.78	3.68E-102
OTHSWSLT35464_at	OthsSGLT35464	PRK00013/chaperonin	RUBISCO SUBUNIT BIN	/	LOC_Os06g02380.2|13106.m00160|protein/3E-27/RNA degradation/3E-27	5.05	4.72E-108	5.85	7.40E-154	0.86	0.370507918
OTHSWCTG09951_s_at	OthsCTG09951	PLN02592/ent-copalyl	/	AT4G02780/Diterpeno	LOC_Os04g09900.1|13104.m00881|protein/4E-57/Diterpenoid biosynthesis; Bio	5.04	2.89E-228	6.86	0	0.73	0.251475711
AP13ITG66210_at	AP13ISTG66210	pfam02365/No apical	/	/	LOC_Os06g51070.1|13106.m05496|protein/7E-97//	4.95	1.53E-46	3.83	1.07E-24	1.29	0.283129979
KanlowCTG40626_s_at	KanlCTG40626	pfam01657/Domain of	SUBFAMILY NOT NAM	/	LOC_Os10g04730.1|13110.m00390|protein/5E-57//	4.93	0	2.67	2.38E-113	1.84	2.50E-06
AP13ITG50188_s_at	AP13ISTG50188	cd00180/Catalytic dor	SUBFAMILY NOT NAM	/	LOC_Os10g04730.1|13110.m00390|protein/1E-91//	4.91	7.20E-242	2.57	1.64E-40	1.91	2.49E-06
OTHSWCTG17675_s_at	OthsCTG17675	pfam00112/Papain far	CYSTEINE PROTEASE/	/	LOC_Os09g27030.2|13109.m02621|protein/7E-67//	4.86	0	2.64	7.67E-125	1.84	0.000728674
KanlowCTG23219_s_at	KanlCTG23219	//	SUBFAMILY NOT NAM	/	LOC_Os04g49890.1|13104.m05101|protein/2E-144//	4.85	0	2.69	8.12E-122	1.8	2.42E-07
OTHSWCTG14105_s_at	OthsCTG14105	//	/	/	///	4.84	0	2.6	1.02E-72	1.86	5.27E-05
AP13CTG07636_at	AP13CTG07636	pfam01490/Transmem	FAMILY NOT NAMED/	/	LOC_Os08g03350.1|13108.m00291|protein/0//	4.84	1.70E-41	4.01	4.19E-26	1.21	0.389634556
AP13ITG72235_s_at						4.84	1.22E-200	10.52	0	0.46	0.000232423
OTHSWCTG11457_at	OthsCTG11457	cd00684/Plant Terpen	/	/	LOC_Os08g07080.1|13108.m00725|protein/9E-106//	4.65	2.28E-84	5.27	9.28E-115	0.88	0.622590513
AP13ITG66423_s_at	AP13ISTG66423	//	/	/	LOC_Os07g06450.1|13107.m00614|protein/2E-19//	4.57	0	3.03	3.45E-26	1.51	1.02E-14
KanlowCTG17154_s_at	KanlCTG17154	pfam00069/Protein kir	SUBFAMILY NOT NAM	/	LOC_Os01g02840.1|13101.m00223|protein/2E-87//	4.54	5.63E-141	6.82	0	0.67	0.142911571
AP13CTG00637_at	AP13CTG00637	pfam00083/Sugar (and	SUBFAMILY NOT NAM	/	LOC_Os10g41190.1|13110.m03832|protein/0//	4.48	0	5.39	0	0.83	0.198236745
KanlowSLT55446_s_at	KanlSGLT55446	PRK12270/alpha-ketog	/	/	LOC_Os06g46740.1|13106.m04941|protein/0.0007//	4.48	0	2.57	5.81E-115	1.74	1.40E-06
AP13ITG63384_s_at	AP13ISTG63384	pfam02956/TT viral or	/	/	LOC_Os03g17680.1|13103.m02113|protein/0.0001//	4.43	6.85E-55	4.53	8.02E-58	0.98	0.892665852
AP13ITG63673-RC_at						4.43	4.30E-155	4.18	1.54E-133	1.06	0.86501752
KanlowCTG35516_at	KanlCTG35516	//	SUBFAMILY NOT NAM	/	LOC_Os01g15580.1|13101.m01725|protein/0.000000000001//	4.33	0	3.8	0	1.14	0.714009859
AP13ITG41562_s_at	AP13ISTG41562	pfam00635/MSP (Maj	VESICLE-ASSOCIATED	/	LOC_Os11g17380.1|13111.m01762|protein/8E-56//	4.31	0	3.29	0	1.31	0.171063098
KanlowCTG11533_s_at	KanlCTG11533	pfam02956/TT viral or	/	/	LOC_Os03g17680.1|13103.m02113|protein/0.00008//	4.25	2.89E-59	4.45	1.76E-66	0.96	0.768834389
AP13CTG30443_at	AP13CTG30443	//	SUBFAMILY NOT NAM	/	LOC_Os11g45600.1|13111.m04557|protein/2E-23//	4.25	0	4.02	0	1.06	0.81106624
AP13ITG44875_at	AP13ISTG44875	PRK12323/DNA polym	/	/	///	4.25	4.97E-69	2.79	3.27E-22	1.52	0.002532083
AP13CTG00450_at	AP13CTG00450	PLN03059/beta-galact	SUBFAMILY NOT NAM	AT3G52840/Galactose	LOC_Os12g24170.1|13112.m02458|protein/0//	4.22	0	3.77	0	1.12	0.519624854
KanlowCTG13191_s_at	KanlCTG13191	PHA03247/large tegun	/	/	LOC_Os09g26999.1|13109.m02618|protein/0.006//	4.11	4.60E-129	4.07	1.29E-125	1.01	0.968770642
AlamoCTG04547_s_at	AlamCTG04547	pfam02713/Domain of	/	/	LOC_Os09g15835.1|13109.m07521|protein/1E-113//	4.08	2.13E-198	2.77	1.75E-66	1.48	0.056240134
AP13CTG17193_at	AP13CTG17193	//	/	/	LOC_Os03g30092.1|13103.m03448|protein/1E-43//	4.01	0	3.09	3.86E-89	1.3	2.26E-10
AP13CTG04635_at	AP13CTG04635	pfam00069/Protein kir	SUBFAMILY NOT NAM	/	LOC_Os06g38340.1|13106.m03968|protein/3E-132//	3.99	1.34E-80	3.03	5.44E-38	1.32	0.202219363
OTHSWCTG27954_at	OthsCTG27954	COG0515/Serine/thre	SUBFAMILY NOT NAM	/	LOC_Os02g40180.1|13102.m04472|protein/4E-51//	3.98	5.60E-162	5.14	0	0.77	0.347275716
AP13CTG65129_s_at	AP13CTG65129	smart00219/Tyrosine I	PROTEIN KINASE ATM	/	LOC_Os12g06670.1|13112.m00686|protein/1E-155//	3.97	9.12E-67	5.32	0	0.75	0.000808527
AP13ITG38393_at	AP13ISTG38393	TIGR03389/laccase, pl	LACCASE/0	/	LOC_Os03g16610.1|13103.m01973|protein/0//	3.97	9.73E-44	7.61	9.81E-210	0.52	7.40E-17
AlamoCTG06651_s_at	AlamCTG06651	//	/	/	///	3.92	3.86E-135	2.76	1.48E-50	1.42	0.000117451
AP13ITG62258-RC_at						3.87	3.62E-64	3.73	1.74E-58	1.04	0.899606165
AP13ITG39900_s_at	AP13ISTG39900	pfam00069/Protein kir	OS12G0249900 PROT	/	LOC_Os12g14610.1|13112.m01551|protein/2E-64//	3.85	2.22E-28	5.75	1.29E-75	0.67	0.004148966
AP13ITG49381_at	AP13ISTG49381	//	/	/	///	3.84	0	4.06	1.20E-88	0.95	0.482493166
OTHSWCTG14285_at	OthsCTG14285	//	/	/	///	3.83	0	2.97	1.73E-191	1.29	0.243914326
OTHSWCTG08671_s_at	OthsCTG08671	TIGR00957/multi drug	SUBFAMILY NOT NAM	/	LOC_Os04g13220.1|13104.m01213|protein/0//	3.81	1.39E-82	4.33	2.56E-115	0.88	0.615770674
AP13ITG54952_at	AP13ISTG54952	//	UNKNOWN PROTEIN/	/	LOC_Os02g36940.1|13102.m04038|protein/7E-59//	3.77	5.79E-71	4.05	7.77E-86	0.93	0.771345593
AP13ITG67384-RC_at						3.76	1.33E-51	4.13	5.53E-66	0.91	0.828890036
OTHSWCTG13588_s_at	OthsCTG13588	PLN02526/acyl-CoA ox	SUBFAMILY NOT NAM	AT3G51840/Fatty acid	LOC_Os05g07090.3|13105.m00774|protein/5E-56/Fatty acid metabolism; alpha	3.74	1.24E-69	3.47	1.12E-56	1.08	0.734875676
AP13ITG41643_s_at	AP13ISTG41643	cd03244/Domain 2 of	SUBFAMILY NOT NAM	/	LOC_Os04g13220.1|13104.m01213|protein/4E-126//	3.72	6.30E-93	4.04	2.64E-115	0.92	0.766502702
KanlowCTG23219_at	KanlCTG23219	//	SUBFAMILY NOT NAM	/	LOC_Os04g49890.1|13104.m05101|protein/2E-144//	3.69	7.06E-105	2.99	4.55E-58	1.24	0.086396878

<table>
<tr><th></th><th></th><th></th><th></th><th></th><th></th><th></th><th></th><th></th><th></th><th></th><th></th></tr>
<tr><td>AP13CTG19859_at</td><td>AP13CTG19859</td><td>cd00180/Catalytic don</td><td>SUBFAMILY NOT NAM</td><td>/</td><td>LOC_Os04g43730.1|13104.m04348|protein/3E-138//</td><td>3.68</td><td>2.82E-225</td><td>3.08</td><td>1.83E-136</td><td>1.19</td><td>0.597594836</td></tr>
<tr><td>AP13ITG65301_at</td><td>AP13ISTG65301</td><td>PHA03249/DNA packa</td><td>/</td><td>/</td><td>LOC_Os01g02130.1|13101.m00151|protein/9E-20//</td><td>3.64</td><td>2.21E-68</td><td>3.9</td><td>4.99E-82</td><td>0.93</td><td>0.758536014</td></tr>
<tr><td>AlamoCTG04793_at</td><td>AlamCTG04793</td><td>cd01459/VWA Copine:</td><td>COPINE/0.000000000</td><td>/</td><td>LOC_Os06g40650.2|13106.m04232|protein/3E-68//</td><td>3.61</td><td>0</td><td>2.51</td><td>6.83E-117</td><td>1.44</td><td>0.014171803</td></tr>
<tr><td>AP13ITG59978_at</td><td>AP13ISTG59978</td><td>cd03185/GST_C family</td><td>GLUTATHIONE S-TRAN</td><td>/</td><td>LOC_Os10g38470.1|13110.m03503|protein/9E-80//</td><td>3.61</td><td>1.70E-52</td><td>5.04</td><td>3.29E-123</td><td>0.72</td><td>0.180214218</td></tr>
<tr><td>AP13CTG24727_at</td><td>AP13CTG24727</td><td>cd05476/Chroloplast N</td><td>CHLOROPLAST NUCLE</td><td>/</td><td>LOC_Os07g34940.1|13107.m03570|protein/8E-137//</td><td>3.59</td><td>3.60E-151</td><td>3.85</td><td>6.48E-182</td><td>0.93</td><td>0.824433241</td></tr>
<tr><td>AP13ITG42171RC_at</td><td>AP13ISTG42171-RC</td><td>pfam02469/Fasciclin d</td><td>PERIOSTIN-RELATED/</td><td>/</td><td>LOC_Os01g06580.1|13101.m00650|protein/5E-67//</td><td>3.59</td><td>4.43E-65</td><td>3.08</td><td>8.72E-43</td><td>1.16</td><td>0.444585704</td></tr>
<tr><td>AP13ITG63170_at</td><td>AP13ISTG63170</td><td>pfam03018/Dirigent-li</td><td>FAMILY NOT NAMED/</td><td>/</td><td>LOC_Os10g18760.1|13110.m01548|protein/0.00000000000002//</td><td>3.57</td><td>1.14E-24</td><td>3.54</td><td>0</td><td>1.01</td><td>0.801121659</td></tr>
<tr><td>AP13CTG14747_s_at</td><td>AP13CTG14747</td><td>PLN02290/cytokinin tr</td><td>SUBFAMILY NOT NAM</td><td>/</td><td>LOC_Os03g25480.1|13103.m03006|protein/0//</td><td>3.53</td><td>2.13E-80</td><td>3.34</td><td>9.19E-69</td><td>1.06</td><td>0.797210625</td></tr>
<tr><td>KanlowCTG42496_s_at</td><td>KanlCTG42496</td><td>PLN02640/glucose-6-p</td><td>FAMILY NOT NAMED/</td><td>AT5G35790/Pentose p</td><td>LOC_Os03g29950.1|13103.m03430|protein/3E-114/Pentose phosphate pathway</td><td>3.52</td><td>4.47E-117</td><td>3.75</td><td>2.47E-139</td><td>0.94</td><td>0.716288558</td></tr>
<tr><td>AlamoCTG02492_x_at</td><td>AlamCTG02492</td><td>smart00813/Alpha-L-a</td><td>/</td><td>/</td><td>LOC_Os11g03730.3|13111.m00315|protein/1E-133//</td><td>3.48</td><td>2.40E-178</td><td>2.58</td><td>4.17E-74</td><td>1.35</td><td>0.116298925</td></tr>
<tr><td>AP13CTG21882_s_at</td><td>AP13CTG21882</td><td>PLN02420/hydrolase, I</td><td>FAMILY NOT NAMED/</td><td>/</td><td>LOC_Os02g50490.1|13102.m05730|protein/0//</td><td>3.47</td><td>8.23E-162</td><td>2.53</td><td>3.40E-63</td><td>1.37</td><td>0.033327474</td></tr>
<tr><td>AP13ITG76663RC_at</td><td>AP13ISTG76663-RC</td><td>PHA03247/large tegun</td><td>/</td><td>/</td><td>LOC_Os05g51780.1|13105.m05599|protein/7E-33//</td><td>3.43</td><td>9.92E-248</td><td>2.89</td><td>6.28E-151</td><td>1.19</td><td>0.374597487</td></tr>
<tr><td>OTHSWCTG10420_at</td><td>OthsCTG10420</td><td>//</td><td>/</td><td>/</td><td>LOC_Os12g25170.1|13112.m02552|protein/8E-40//</td><td>3.4</td><td>4.10E-80</td><td>3.07</td><td>5.86E-60</td><td>1.11</td><td>0.597229179</td></tr>
<tr><td>AP13ITG64790RC_at</td><td>AP13ISTG64790-RC</td><td>PHA03247/large tegun</td><td>/</td><td>/</td><td>LOC_Os01g60930.1|13101.m06486|protein/7E-101//</td><td>3.37</td><td>1.60E-33</td><td>3.06</td><td>8.49E-26</td><td>1.1</td><td>0.667687412</td></tr>
<tr><td>KanlowCTG22618_s_at</td><td>KanlCTG22618</td><td>PHA03245/large tegun</td><td>/</td><td>/</td><td>///</td><td>3.37</td><td>9.69E-08</td><td>3.33</td><td>1.54E-07</td><td>1.01</td><td>0.978736632</td></tr>
<tr><td>OTHSWCTG07191_at</td><td>OthsCTG07191</td><td>//</td><td>/</td><td>/</td><td>///</td><td>3.36</td><td>3.81E-13</td><td>3.32</td><td>8.38E-13</td><td>1.01</td><td>0.967344262</td></tr>
<tr><td>OTHSWSLT35408_at</td><td>OthsSGLT35408</td><td>//</td><td>/</td><td>/</td><td>LOC_Os01g24340.1|13101.m02574|protein/0.0000000000000002//</td><td>3.24</td><td>1.50E-85</td><td>3.62</td><td>7.67E-117</td><td>0.89</td><td>0.381885648</td></tr>
<tr><td>OTHSWCTG35380_s_at</td><td>OthsCTG35380</td><td>//</td><td>SUBFAMILY NOT NAM</td><td>/</td><td>LOC_Os01g02290.1|13101.m00169|protein/1E-20//</td><td>3.23</td><td>8.66E-107</td><td>6.35</td><td>0</td><td>0.51</td><td>0.026978284</td></tr>
<tr><td>AP13CTG04921_s_at</td><td>AP13CTG04921</td><td>pfam01535/PPR repea</td><td>FAMILY NOT NAMED/</td><td>/</td><td>LOC_Os10g28600.1|13110.m02438|protein/0//</td><td>3.2</td><td>0</td><td>3.05</td><td>1.25E-150</td><td>1.05</td><td>0.32875217</td></tr>
<tr><td>AP13CTG23391_at</td><td>AP13CTG23391</td><td>//</td><td>SUBFAMILY NOT NAM</td><td>/</td><td>LOC_Os03g62430.1|13103.m06892|protein/3E-50//</td><td>3.16</td><td>3.59E-38</td><td>3.66</td><td>2.38E-57</td><td>0.86</td><td>0.312236418</td></tr>
<tr><td>AP13ITG45589_s_at</td><td>AP13ISTG45589</td><td>//</td><td>/</td><td>/</td><td>///</td><td>3.16</td><td>0</td><td>3.23</td><td>1.51E-66</td><td>0.98</td><td>0.781788464</td></tr>
<tr><td>KanlowCTG42196_s_at</td><td>KanlCTG42196</td><td>cd00180/Catalytic don</td><td>OS04G0109100 PROT</td><td>/</td><td>LOC_Os04g01874.1|13104.m00100|protein/3E-84//</td><td>3.15</td><td>7.70E-43</td><td>2.74</td><td>1.06E-28</td><td>1.15</td><td>0.552279355</td></tr>
<tr><td>AP13CTG29146_at</td><td>AP13CTG29146</td><td>pfam03018/Dirigent-li</td><td>OS10G0335000 PROT</td><td>/</td><td>LOC_Os10g18760.1|13110.m01548|protein/4E-21//</td><td>3.14</td><td>3.42E-62</td><td>3.07</td><td>0</td><td>1.02</td><td>0.45687962</td></tr>
<tr><td>AP13ITG45888_s_at</td><td>AP13ISTG45888</td><td>//</td><td>SUBFAMILY NOT NAM</td><td>/</td><td>LOC_Os07g43810.1|13107.m04605|protein/6E-58//</td><td>3.07</td><td>9.09E-137</td><td>3.11</td><td>4.27E-142</td><td>0.99</td><td>0.940878264</td></tr>
<tr><td>AP13ITG55233_at</td><td>AP13ISTG55233</td><td>PLN02640/glucose-6-p</td><td>FAMILY NOT NAMED/</td><td>AT5G35790/Pentose p</td><td>LOC_Os03g29950.1|13103.m03430|protein/0/Pentose phosphate pathway; Glu</td><td>3.07</td><td>2.86E-94</td><td>3.09</td><td>7.14E-96</td><td>0.99</td><td>0.964090129</td></tr>
<tr><td>KanlowCTG22694_at</td><td>KanlCTG22694</td><td>//</td><td>UNKNOWN PROTEIN/</td><td>/</td><td>LOC_Os02g36940.1|13102.m04038|protein/6E-59//</td><td>3.07</td><td>5.45E-48</td><td>3.16</td><td>3.40E-52</td><td>0.97</td><td>0.893850744</td></tr>
<tr><td>KanlowCTG42715_at</td><td>KanlCTG42715</td><td>PLN00113/leucine-rich</td><td>SUBFAMILY NOT NAM</td><td>/</td><td>LOC_Os02g40240.2|13102.m04480|protein/2E-81//</td><td>3.05</td><td>8.79E-53</td><td>4.09</td><td>2.62E-117</td><td>0.75</td><td>0.120951283</td></tr>
<tr><td>AP13CTG00450_s_at</td><td>AP13CTG00450</td><td>PLN03059/beta-galact</td><td>SUBFAMILY NOT NAM</td><td>AT3G52840/Galactose</td><td>LOC_Os12g24170.1|13112.m02458|protein/0//</td><td>3.04</td><td>3.01E-202</td><td>2.79</td><td>3.91E-156</td><td>1.09</td><td>0.578334949</td></tr>
<tr><td>AP13CTG11976_s_at</td><td>AP13CTG11976</td><td>PLN02687/flavonoid 3'</td><td>SUBFAMILY NOT NAM</td><td>/</td><td>LOC_Os08g01450.1|13108.m00065|protein/8E-172//</td><td>3.03</td><td>2.59E-104</td><td>2.96</td><td>1.37E-97</td><td>1.02</td><td>0.897735784</td></tr>
<tr><td>KanlowCTG23500_s_at</td><td>KanlCTG23500</td><td>//</td><td>UDP-GLUCOSYLTRANS</td><td>/</td><td>LOC_Os07g32010.1|13107.m03237|protein/2E-88//</td><td>2.99</td><td>7.12E-66</td><td>2.65</td><td>4.48E-46</td><td>1.13</td><td>0.601092244</td></tr>
<tr><td>AP13ITG71998-RC_at</td><td></td><td></td><td></td><td></td><td></td><td>2.98</td><td>5.19E-29</td><td>2.78</td><td>7.41E-24</td><td>1.07</td><td>0.648224917</td></tr>
<tr><td>AP13CTG09743_at</td><td>AP13CTG09743</td><td>pfam02536/mTERF. Th</td><td>SUBFAMILY NOT NAM</td><td>/</td><td>LOC_Os07g39430.1|13107.m04084|protein/0//</td><td>2.95</td><td>4.89E-40</td><td>3.31</td><td>3.77E-55</td><td>0.89</td><td>0.460711786</td></tr>
<tr><td>AP13CTG00528_at</td><td>AP13CTG00528</td><td>pfam04884/Protein of</td><td>SUBFAMILY NOT NAM</td><td>/</td><td>LOC_Os03g11500.1|13103.m01297|protein/9E-156//</td><td>2.94</td><td>3.39E-81</td><td>2.63</td><td>6.14E-58</td><td>1.12</td><td>0.420762581</td></tr>
<tr><td>AlamoCTG03711_s_at</td><td>AlamCTG03711</td><td>//</td><td>/</td><td>/</td><td>LOC_Os09g36290.1|13109.m03573|protein/1E-77//</td><td>2.88</td><td>1.18E-61</td><td>2.69</td><td>3.14E-50</td><td>1.07</td><td>0.72842844</td></tr>
<tr><td>AP13ITG65392_at</td><td>AP13ISTG65392</td><td>PLN02152/UDP-glucos</td><td>PUTATIVE GLUCOSYLT</td><td>AT4G14090/Anthocya</td><td>LOC_Os05g08750.1|13105.m00970|protein/5E-150//</td><td>2.87</td><td>2.62E-22</td><td>3.73</td><td>1.77E-45</td><td>0.77</td><td>0.042591239</td></tr>
<tr><td>AP13CTG05682_s_at</td><td>AP13CTG05682</td><td>cd03407/A subgroup c</td><td>BAND 7 FAMILY PROT</td><td>/</td><td>LOC_Os05g51420.2|13105.m05539|protein/2E-149//</td><td>2.86</td><td>6.31E-41</td><td>3.87</td><td>7.21E-95</td><td>0.74</td><td>0.093134852</td></tr>
<tr><td>AP13ITG50374_s_at</td><td>AP13ISTG50374</td><td>pfam03069/Acetamida</td><td>/</td><td>AT4G37560/Glyoxylat</td><td>LOC_Os01g55950.1|13101.m05909|protein/5E-122/Glyoxylate and dicarboxylat</td><td>2.85</td><td>1.55E-12</td><td>3.04</td><td>5.91E-15</td><td>0.94</td><td>0.766762</td></tr>
<tr><td>OTHSWCTG14772_at</td><td>OthsCTG14772</td><td>pfam03018/Dirigent-li</td><td>OS10G0335000 PROT</td><td>/</td><td>LOC_Os10g18870.1|13110.m01559|protein/0.000000000006//</td><td>2.82</td><td>1.97E-43</td><td>3.03</td><td>0</td><td>0.93</td><td>0.344843681</td></tr>
<tr><td>AP13CTG24597_at</td><td>AP13CTG24597</td><td>PHA03247/large tegun</td><td>OS03G0335300 PROT</td><td>/</td><td>LOC_Os04g52190.1|13104.m05345|protein/0//</td><td>2.82</td><td>2.42E-51</td><td>2.59</td><td>1.62E-39</td><td>1.09</td><td>0.69609287</td></tr>
<tr><td>AP13CTG22276_s_at</td><td>AP13CTG22276</td><td>cd03419/Glutaredoxin</td><td>GLUTAREDOXIN/1.6E-</td><td>/</td><td>LOC_Os02g40500.1|13102.m04514|protein/5E-47//</td><td>2.8</td><td>8.10E-37</td><td>2.89</td><td>9.78E-41</td><td>0.97</td><td>0.883091726</td></tr>
<tr><td>AP13CTG14697_s_at</td><td>AP13CTG14697</td><td>pfam00069/Protein kir</td><td>SUBFAMILY NOT NAM</td><td>/</td><td>LOC_Os04g03830.1|13104.m00305|protein/4E-75//</td><td>2.79</td><td>8.87E-14</td><td>3.34</td><td>1.58E-22</td><td>0.83</td><td>0.153062513</td></tr>
<tr><td>AP13CTG15597_at</td><td>AP13CTG15597</td><td>pfam12049/Protein of</td><td>/</td><td>/</td><td>LOC_Os02g58120.1|13102.m06728|protein/3E-120//</td><td>2.78</td><td>0</td><td>2.6</td><td>1.02E-58</td><td>1.07</td><td>0.221478802</td></tr>
<tr><td>KanlowSLT52258_at</td><td>KanlSGLT52258</td><td>//</td><td>/</td><td>/</td><td>LOC_Os05g39130.1|13105.m04119|protein/0.0000000004//</td><td>2.78</td><td>1.39E-78</td><td>2.72</td><td>3.90E-73</td><td>1.02</td><td>0.906445296</td></tr>
<tr><td>AP13ITG47602_s_at</td><td>AP13ISTG47602</td><td>cd03407/A subgroup c</td><td>BAND 7 FAMILY PROT</td><td>/</td><td>LOC_Os05g51420.7|13105.m05540|protein/3E-85//</td><td>2.77</td><td>1.38E-30</td><td>4.29</td><td>1.09E-101</td><td>0.64</td><td>0.001517568</td></tr>
<tr><td>AP13ITG41137_at</td><td>AP13ISTG41137</td><td>cd01989/The N-termir</td><td>/</td><td>/</td><td>LOC_Os09g39650.3|13109.m03981|protein/4E-86//</td><td>2.73</td><td>3.85E-101</td><td>4.1</td><td>0</td><td>0.67</td><td>0.000440838</td></tr>
<tr><td>KanlowCTG41582_s_at</td><td>KanlCTG41582</td><td>TIGR03389/laccase, pl</td><td>LACCASE/3E-228</td><td>/</td><td>LOC_Os03g16610.1|13103.m01973|protein/0//</td><td>2.73</td><td>1.76E-27</td><td>4.52</td><td>1.79E-108</td><td>0.6</td><td>1.52E-05</td></tr>
<tr><td>AP13CTG21472_s_at</td><td>AP13CTG21472</td><td>pfam05140/ResB-like f</td><td>/</td><td>/</td><td>LOC_Os03g55970.1|13103.m06098|protein/0//</td><td>2.67</td><td>0</td><td>2.57</td><td>1.83E-28</td><td>1.04</td><td>0.549155944</td></tr>
<tr><td>OTHSWCTG17938_at</td><td>OthsCTG17938</td><td>PLN00113/leucine-rich</td><td>SUBFAMILY NOT NAM</td><td>/</td><td>LOC_Os02g40180.1|13102.m04472|protein/4E-62//</td><td>2.67</td><td>5.38E-15</td><td>3.09</td><td>1.53E-22</td><td>0.86</td><td>0.483485158</td></tr>
<tr><td>OTHSWSLT26755_at</td><td>OthsSGLT26755</td><td>//</td><td>/</td><td>/</td><td>///</td><td>2.66</td><td>1.12E-71</td><td>3.96</td><td>5.08E-224</td><td>0.67</td><td>1.82E-07</td></tr>
<tr><td>AP13CTG06087_s_at</td><td>AP13CTG06087</td><td>COG2802/Uncharacter</td><td>FAMILY NOT NAMED/</td><td>/</td><td>LOC_Os03g29540.3|13103.m12878|protein/1E-68//</td><td>2.65</td><td>2.71E-22</td><td>3.19</td><td>3.19E-38</td><td>0.83</td><td>0.133185301</td></tr>
<tr><td>AP13ITG76471_s_at</td><td>AP13ISTG76471</td><td>//</td><td>SUBFAMILY NOT NAM</td><td>/</td><td>LOC_Os03g10100.1|13103.m01126|protein/0//</td><td>2.65</td><td>7.86E-22</td><td>2.85</td><td>6.78E-27</td><td>0.93</td><td>0.519980024</td></tr>
<tr><td>AP13CTG06369_at</td><td>AP13CTG06369</td><td>cd01876/The YihA (Eng</td><td>FAMILY NOT NAMED/</td><td>/</td><td>LOC_Os01g53150.2|13101.m05568|protein/1E-132//</td><td>2.65</td><td>2.11E-152</td><td>2.76</td><td>3.27E-172</td><td>0.96</td><td>0.781095061</td></tr>
<tr><td>AP13CTG32494_s_at</td><td>AP13CTG32494</td><td>//</td><td>OS04G0192800 PROT</td><td>/</td><td>LOC_Os11g14340.1|13111.m01470|protein/3E-54//</td><td>2.63</td><td>0</td><td>4.49</td><td>3.63E-280</td><td>0.59</td><td>8.30E-37</td></tr>
<tr><td>KanlowCTG43401_s_at</td><td>KanlCTG43401</td><td>pfam04884/Protein of</td><td>SUBFAMILY NOT NAM</td><td>/</td><td>LOC_Os03g11500.1|13103.m01297|protein/5E-37//</td><td>2.63</td><td>3.50E-104</td><td>2.51</td><td>1.06E-90</td><td>1.04</td><td>0.743877262</td></tr>
<tr><td>AP13CTG11164_s_at</td><td>AP13CTG11164</td><td>PLN02189/cellulose sy</td><td>SUBFAMILY NOT NAM</td><td>/</td><td>LOC_Os09g25490.1|13109.m02439|protein/7E-139//</td><td>2.61</td><td>2.80E-07</td><td>6.42</td><td>1.15E-66</td><td>0.41</td><td>1.14E-10</td></tr>
<tr><td>AP13CTG13909_s_at</td><td>AP13CTG13909</td><td>//</td><td>/</td><td>/</td><td>///</td><td>2.6</td><td>5.80E-65</td><td>2.94</td><td>5.99E-95</td><td>0.88</td><td>0.248814027</td></tr>
<tr><td>AP13ITG42951-RC_at</td><td></td><td></td><td></td><td></td><td></td><td>2.57</td><td>6.08E-23</td><td>7.53</td><td>0</td><td>0.34</td><td>0</td></tr>
<tr><td>OTHSWCTG26966_s_at</td><td>OthsCTG26966</td><td>//</td><td>OS12G0477100 PROT</td><td>/</td><td>LOC_Os06g16790.1|13106.m01851|protein/0.000000005//</td><td>2.56</td><td>2.51E-42</td><td>2.52</td><td>1.59E-40</td><td>1.01</td><td>0.940058441</td></tr>
<tr><td>AP13CTG00613_at</td><td>AP13CTG00613</td><td>cd00180/Catalytic don</td><td>FAMILY NOT NAMED/</td><td>/</td><td>LOC_Os12g14610.1|13112.m01551|protein/5E-60//</td><td>2.56</td><td>1.61E-57</td><td>2.7</td><td>1.37E-67</td><td>0.95</td><td>0.563172712</td></tr>
<tr><td>AP13CTG20027_at</td><td>AP13CTG20027</td><td>//</td><td>/</td><td>/</td><td>///</td><td>2.55</td><td>1.45E-10</td><td>2.54</td><td>1.29E-09</td><td>1</td><td>0.966882108</td></tr>
<tr><td>KanlowCTG36954_at</td><td>KanlCTG36954</td><td>PRK05653/3-ketoacyl-</td><td>SUBFAMILY NOT NAM</td><td>AT2G29150/Tropane,</td><td>LOC_Os11g43200.1|13111.m04296|protein/2E-90//</td><td>2.55</td><td>4.10E-32</td><td>2.99</td><td>5.14E-52</td><td>0.85</td><td>0.145765565</td></tr>
<tr><td>KanlowCTG15086_s_at</td><td>KanlCTG15086</td><td>//</td><td>/</td><td>/</td><td>LOC_Os01g03110.1|13101.m00262|protein/2E-87//</td><td>2.52</td><td>3.78E-45</td><td>2.82</td><td>2.12E-64</td><td>0.89</td><td>0.484160365</td></tr>
<tr><td>AP13CTG52756_s_at</td><td>AP13CTG52756</td><td>cd06899/legume lectir</td><td>SUBFAMILY NOT NAM</td><td>/</td><td>LOC_Os07g03900.1|13107.m00328|protein/5E-116//</td><td>2.51</td><td>2.38E-13</td><td>2.86</td><td>1.75E-19</td><td>0.88</td><td>0.547546191</td></tr>
</table>

Table S4. List of commonly down-regulated genes with more than 2.5 fold change between Se/60 and Mo/60 comparisons

Genes not corrolated with lesion development (Se/Mo>1) were ruled out and highlighted in blue. P, Bonferroni-corrected P value.

probeset_id	PviUT sequence ID	CDD annotation	Panther annotation	Arabidopsis hit	Rice hit	Ratio(Se/60)	P	Ratio(Mo/60)	P	Ratio(Se/Mo)	P
KanlowCTG15097_at	KanlCTG15097	pfam02902/Ulp1 protea	/	/	LOC_Os08g27759.1|13108.m02864|protein/7E-23//	0.06	0	0.08	0	0.68	5.06E-18
AP13ITG56127_x_at	AP13ISTG56127	pfam00235/Profilin./1E-	FAMILY NOT NAMED/1.1E-	/	LOC_Os10g17680.1|13110.m01449|protein/2E-72//	0.1	0	0.12	0	0.79	0.227125134
AP13CTG25577_at	AP13CTG25577	//	/	/	///	0.11	2.12E-96	0.06	0	1.7	5.08E-06
AP13CTG40946_s_at	AP13CTG40946	pfam09713/Plant protei	/	/	LOC_Os05g38680.1|13105.m04062|protein/8E-33//	0.13	1.12E-32	0.36	8.18E-12	0.38	0.002086835
AP13ITG64729_at	AP13ISTG64729	TIGR02189/Glutaredoxir	GLUTAREDOXIN/6.4E-43	/	LOC_Os12g35330.1|13112.m03614|protein/3E-45//	0.13	3.84E-156	0.19	6.84E-15	0.7	0.085064035
AP13ITG70427_at	AP13ISTG70427	pfam03242/Late embryc	/	/	LOC_Os01g21250.1|13101.m02261|protein/0.0000002//	0.13	7.50E-106	0.22	7.63E-09	0.59	0.021712227
AP13ITG37591_at	AP13ISTG37591	//	/	/	///	0.14	4.90E-37	0.19	9.90E-44	0.77	0.45993667
AlamoSLT15807_at	AlamSGLT15807	//	/	/	LOC_Os04g51090.1|13104.m05196|protein/0.00001//	0.15	5.69E-150	0.19	1.14E-31	0.82	0.292689791
AP13ITG63308-RC_at						0.15	2.15E-125	0.18	3.04E-148	0.83	0.341115395
AlamoCTG06663_at	AlamCTG06663	//	/	/	///	0.16	0	0.12	0	1.31	0.001114078
AP13CTG37783_s_at	AP13CTG37783	pfam02956/TT viral orf 1	/	/	LOC_Os06g43810.1|13106.m04592|protein/0.00000000006//	0.16	0	0.17	7.74E-168	0.98	0.873664064
AP13ITG51564_s_at	AP13ISTG51564	pfam01073/3-beta hydr	3 BETA-HYDROXYSTEROID I	/	LOC_Os03g29150.1|13103.m03345|protein/5E-51//	0.16	9.40E-12	0.31	2.47E-25	0.53	0.028458665
AP13ITG60650_at	AP13ISTG60650	pfam06376/Protein of u	/	/	LOC_Os05g12580.1|13105.m01366|protein/0.0000000000002//	0.16	1.09E-93	0.11	0	1.4	0.025136064
AP13CTG02927_at	AP13CTG02927	pfam01073/3-beta hydr	3 BETA-HYDROXYSTEROID I	AT4G33360/Flavono	LOC_Os03g29170.2|13103.m03348|protein/2E-119/Amino sugar ar	0.17	2.40E-10	0.32	3.97E-23	0.51	0.021452177
KanlowCTG25668_at	KanlCTG25668	TIGR02882/cytochrome	/	/	LOC_Os04g51090.1|13104.m05196|protein/5E-21//	0.17	0	0.29	4.92E-24	0.59	0.011423108
KanlowCTG26782_s_a	KanlCTG26782	cd00693/Horseradish pe	/	AT5G05340/Phenyla	LOC_Os03g32050.1|13103.m03658|protein/6E-78/Phenylalanine m	0.17	8.80E-96	0.29	4.39E-24	0.58	0.002209208
VS16ITG03134_s_at	VS16ISTG03134	cd01837/SGNH_plant_li	LATERAL SIGNALING TARGE	/	LOC_Os06g06250.2|13106.m10511|protein/3E-179//	0.17	0	0.11	0	1.48	0.007109794
KanlowCTG42704_at	KanlCTG42704	pfam09507/DNA polyme	/	/	LOC_Os04g51090.1|13104.m05196|protein/1E-53//	0.18	3.40E-127	0.21	2.04E-58	0.85	0.331619905
VS16ITG11260_at	VS16ISTG11260	//	/	/	///	0.18	4.42E-97	0.26	8.84E-11	0.69	0.038220887
AP13CTG14980_at	AP13CTG14980	//	/	AT4G13710/Pentose	LOC_Os04g05050.1|13104.m00426|protein/0/Pentose and glucuro	0.19	0	0.21	3.57E-18	0.91	0.714292151
AP13CTG16698_s_at	AP13CTG16698	cd00693/Horseradish pe	/	AT5G14130/Phenyla	LOC_Os08g42030.1|13108.m04518|protein/3E-125/Phenylalanine	0.19	8.33E-61	0.15	2.11E-132	1.26	0.243719571
AP13ITG74644_x_at	AP13ISTG74644	PLN03023/expansin-rela	/	/	LOC_Os10g40700.1|13110.m03773|protein/1E-104//	0.19	1.85E-121	0.24	6.48E-21	0.79	0.140259782
AP13CTG09520_at	AP13CTG09520	COG2198/FOG: HPt dom	/	/	LOC_Os05g09410.1|13105.m01049|protein/1E-70//	0.2	9.92E-28	0.37	3.21E-12	0.54	0.020605124
OTHSWSLT24122_s_at	OthsSGLT24122	PHA03247/large tegume	/	/	///	0.2	0	0.08	0	2.49	4.74E-15
AP13ITG72073_at	AP13ISTG72073	//	UNCHARACTERIZED/4.8E-2	/	LOC_Os04g39360.1|13104.m03811|protein/0.00000000000002//	0.21	1.51E-27	0.23	0	0.9	0.086649303
KanlowCTG00319_at	KanlCTG00319	PLN00166/aquaporins o	TONOPLAST INTRINSIC PRC	/	LOC_Os06g22960.1|13106.m02470|protein/4E-136//	0.21	1.14E-105	0.28	6.64E-56	0.73	0.041362562
KanlowCTG06768_at	KanlCTG06768	pfam01679/Uncharacter	FAMILY NOT NAMED/1.9E-	/	LOC_Os07g44180.1|13107.m04647|protein/0.000000000000000C	0.21	9.03E-209	0.12	9.69E-118	1.67	0.001143448
AP13CTG28955_at	AP13CTG28955	cd00693/Horseradish pe	/	AT1G05260/Phenyla	LOC_Os10g39170.1|13110.m03584|protein/1E-140//	0.22	0	0.19	8.29E-62	1.19	7.66E-08
AP13ITG54183_at	AP13ISTG54183	//	/	/	///	0.22	0	0.28	0	0.77	0.326201408
VS16ITG11260_s_at	VS16ISTG11260	//	/	/	///	0.22	1.36E-35	0.35	1.20E-11	0.63	0.04213291
AP13CTG30547_s_at	AP13CTG30547	//	/	/	///	0.23	0	0.27	2.02E-81	0.85	0.013168887
AP13ITG76439_at	AP13ISTG76439	//	PUTATIVE UNCHARACTERIZ	/	LOC_Os01g20830.1|13101.m02207|protein/0.0000000000003//	0.23	3.39E-37	0.37	4.20E-20	0.63	0.02425193
AlamoCTG14387_s_at	AlamCTG14387	//	FAMILY NOT NAMED/0.000	AT2G06510/DNA rep	LOC_Os03g08400.1|13103.m00965|protein/2E-57//	0.24	0	0.34	0	0.7	0.161096627
AP13CTG23694_at	AP13CTG23694	pfam04770/ZF-HD prote	/	/	LOC_Os11g03420.1|13111.m00281|protein/2E-28//	0.24	8.33E-30	0.25	5.94E-84	0.96	0.804271346
AP13CTG26572_s_at	AP13CTG26572	pfam06728/GPI transam	FAMILY NOT NAMED/8.8E-	AT1G63110/Glycosyl	LOC_Os02g46350.1|13102.m05202|protein/3E-37/Glycosylphospha	0.24	8.46E-18	0.33	1.76E-16	0.72	0.254891481
KanlowCTG25136_s_a	KanlCTG25136	PLN02336/phosphoetha	SUBFAMILY NOT NAMED/5	AT3G18000/Glycero	LOC_Os05g47540.1|13105.m05037|protein/9E-83//	0.24	3.29E-229	0.36	6.52E-08	0.66	2.55E-07
KanlowCTG37283_s_a	KanlCTG37283	cd01837/SGNH_plant_li	LATERAL SIGNALING TARGE	/	LOC_Os06g05630.1|13106.m00551|protein/2E-136//	0.24	0	0.23	0	1.02	0.833197369
OTHSWCTG25697_at	OthsCTG25697	//	/	/	///	0.25	1.11E-69	0.28	9.17E-10	0.9	0.506168018
AP13ITG41402_s_at	AP13ISTG41402	PHA03247/large tegume	/	/	LOC_Os01g07240.1|13101.m00724|protein/8E-20//	0.25	0	0.24	0	1.05	0.706627797
AP13ITG63962-RC_at						0.25	3.01E-39	0.28	1.28E-78	0.89	0.422833378
KanlowCTG47101_at	KanlCTG47101	PLN02270/phospholipas	OS09G0421300 PROTEIN/8	AT4G11840/Glycero	LOC_Os09g25390.1|13109.m02428|protein/2E-39/Glycerophospho	0.25	1.96E-71	0.34	3.84E-118	0.74	0.001791649
OTHSWCTG07240_at	OthsCTG07240	PLN00191/enolase/2E-1	FAMILY NOT NAMED/0.000	AT2G36530/Glycolys	LOC_Os10g08550.5|13110.m00739|protein/0.000000000001/Glyco	0.25	0	0.24	0	1.04	0.883510469
OTHSWCTG07571_s_a	OthsCTG07571	cd02947/TRX family; cor	SUBFAMILY NOT NAMED/5	/	LOC_Os09g38670.2|13109.m03857|protein/0.0000000000000002	0.25	3.28E-23	0.11	0	2.38	4.54E-10
AP13CTG11853_at	AP13CTG11853	smart00102/Actin depol	FAMILY NOT NAMED/5.8E-	/	LOC_Os05g02250.2|13105.m00186|protein/5E-58//	0.26	0	0.36	0	0.72	0.241783372
AP13ITG41762-RC_at						0.26	7.86E-13	0.27	3.29E-227	0.96	0.598875941
KanlowCTG43989_s_a	KanlCTG43989	PLN00113/leucine-rich r	FAMILY NOT NAMED/2.5E-	/	LOC_Os07g31840.1|13107.m03222|protein/2E-73//	0.26	0	0.26	0	1.02	0.903812123
AP13CTG22648_at	AP13CTG22648	//	SUBFAMILY NOT NAMED/9	/	LOC_Os05g49240.1|13105.m05266|protein/4E-36//	0.27	2.50E-31	0.39	5.76E-08	0.71	0.069229322
AP13CTG28423_s_at	AP13CTG28423	PHA03307/transcription	SUBFAMILY NOT NAMED/7	/	LOC_Os11g01330.1|13111.m00041|protein/0//	0.27	5.78E-17	0.4	1.20E-21	0.69	0.048578624
AP13ITG40815_s_at	AP13ISTG40815	cd01837/SGNH_plant_li	LATERAL SIGNALING TARGE	/	LOC_Os01g42730.1|13101.m04398|protein/3E-81//	0.28	0	0.2	0	1.42	0.255161812
KanlowCTG20903_s_a	KanlCTG20903	cd00403/Ribosomal prot	PBK1-RELATED/4.9E-20	/	LOC_Os05g48422.1|13105.m05151|protein/4E-54//	0.28	1.57E-36	0.24	2.88E-28	1.16	0.497490022
KanlowCTG37283_at	KanlCTG37283	cd01837/SGNH_plant_li	LATERAL SIGNALING TARGE	/	LOC_Os06g05630.1|13106.m00551|protein/2E-136//	0.28	1.23E-66	0.28	1.82E-215	0.98	0.819121686
AP13CTG14141_s_at	AP13CTG14141	TIGR00393/KpsF/GutQ f	CAPSULE EXPRESSION PRO	/	LOC_Os02g06360.1|13102.m00699|protein/4E-101//	0.29	0	0.35	0	0.84	0.602108082
AP13CTG16272_s_at	AP13CTG16272	pfam04483/Protein of u	/	/	LOC_Os09g11520.1|13109.m01034|protein/3E-59//	0.29	1.91E-14	0.35	1.64E-38	0.84	0.252762848
AP13CTG23886_s_at	AP13CTG23886	COG2072/Predicted flav	MONOOXYGENASE/2.5E-2	AT5G43890/Tryptop	LOC_Os04g03980.1|13104.m00320|protein/0/Tryptophan metabol	0.29	4.94E-12	0.33	0	0.9	0.00027368
AP13CTG27072_s_at	AP13CTG27072	PLN00113/leucine-rich r	OS05G0180300 PROTEIN/3	/	LOC_Os05g08770.2|13105.m10346|protein/1E-159//	0.29	1.70E-45	0.23	9.38E-36	1.27	0.203384759
AP13ITG58736_s_at	AP13ISTG58736	pfam02519/Auxin respo	/	/	LOC_Os09g37394.1|13109.m03709|protein/3E-49//	0.29	1.33E-20	0.27	0	1.07	0.284197316
AP13ITG66747_at	AP13ISTG66747	PRK12678/transcription	/	/	LOC_Os04g31350.1|13104.m02996|protein/1E-46//	0.29	1.64E-260	0.2	0	1.43	4.18E-10
KanlowCTG26938_s_a	KanlCTG26938	pfam08161/NUC173 dor	UNCHARACTERIZED NODUI	/	LOC_Os01g72780.1|13101.m07932|protein/2E-21//	0.29	4.98E-50	0.28	0	1.05	0.44736041
KanlowCTG42674_s_a	KanlCTG42674	//	/	/	///	0.29	2.95E-17	0.17	6.24E-141	1.69	0.000253248
AP13CTG31634_at	AP13CTG31634	//	/	/	///	0.3	0	0.33	0	0.9	0.608941651
AP13ITG37441_at	AP13ISTG37441	PLN02236/choline kinas	SUBFAMILY NOT NAMED/7	/	LOC_Os05g45880.1|13105.m04881|protein/1E-159/Glycerophosph	0.3	0	0.25	0	1.19	0.684178316
AP13ITG38712_s_at	AP13ISTG38712	cd02176/Xyloglucan end	/	/	LOC_Os10g39840.1|13110.m03671|protein/3E-119//	0.3	7.50E-79	0.34	1.07E-13	0.89	0.310751326
AP13ITG72601_s_at	AP13ISTG72601	PRK06664/flagellar hook	EUKARYOTIC INITIATION FA	AT5G57870/RNA tra	LOC_Os10g11240.1|13110.m01019|protein/2E-32//	0.3	1.26E-199	0.34	1.72E-67	0.91	0.18031122
KanlowCTG30657_s_a	KanlCTG30657	cd00083/Helix-loop-heli	UPSTREAM TRANSCRIPTIOI	/	LOC_Os03g07540.1|13103.m00847|protein/3E-24//	0.3	5.32E-84	0.28	0	1.07	0.262353252

<table>
<tr><th></th><th></th><th></th><th></th><th></th><th></th><th></th><th></th><th></th><th></th><th></th><th></th></tr>
<tr><td>OTHSWCTG17826_s_a</td><td>OthsCTG17826</td><td>//</td><td>LYSOSOMAL ALPHA-MANN</td><td>AT5G66150/Other gl</td><td>LOC_Os10g05069.1|13110.m00422|protein/8E-68/Other glycan de</td><td>0.3</td><td>0</td><td>0.38</td><td>0</td><td>0.8</td><td>0.552822806</td></tr>
<tr><td>KanlowCTG08571_s_a</td><td>KanlCTG08571</td><td>pfam00827/Ribosomal L</td><td>FAMILY NOT NAMED/6.3E-</td><td>AT4G16720/Ribosom</td><td>LOC_Os05g19370.1|13105.m02044|protein/1E-100/Ribosome/4E-</td><td>0.31</td><td>1.30E-25</td><td>0.27</td><td>1.04E-21</td><td>1.13</td><td>0.585412442</td></tr>
<tr><td>AP13ITG61328_at</td><td>AP13ISTG61328</td><td>pfam05498/Rapid ALkali</td><td>/</td><td>/</td><td>LOC_Os02g44940.1|13102.m05036|protein/0.009//</td><td>0.31</td><td>2.62E-62</td><td>0.4</td><td>2.18E-19</td><td>0.78</td><td>0.034968274</td></tr>
<tr><td>AP13ITG64329_at</td><td>AP13ISTG64329</td><td>//</td><td>/</td><td>/</td><td>///</td><td>0.31</td><td>8.46E-216</td><td>0.25</td><td>1.03E-62</td><td>1.26</td><td>0.004083766</td></tr>
<tr><td>AP13ITG74038_at</td><td>AP13ISTG74038</td><td>PLN02294/cytochrome c</td><td>CYTOCHROME C OXIDASE S</td><td>AT3G15640/Oxidativ</td><td>LOC_Os01g42650.1|13101.m04385|protein/1E-30/Oxidative phosp</td><td>0.31</td><td>3.17E-27</td><td>0.26</td><td>1.66E-40</td><td>1.18</td><td>0.393664079</td></tr>
<tr><td>KanlowCTG30345_s_a</td><td>KanlCTG30345</td><td>cd00693/Horseradish pe</td><td>/</td><td>AT2G18980/Phenyla</td><td>LOC_Os06g48030.1|13106.m05104|protein/3E-49/Phenylalanine m</td><td>0.31</td><td>1.05E-51</td><td>0.34</td><td>1.13E-32</td><td>0.92</td><td>0.535169912</td></tr>
<tr><td>KanlowCTG39226_s_a</td><td>KanlCTG39226</td><td>pfam01074/Glycosyl hyc</td><td>LYSOSOMAL ALPHA-MANN</td><td>AT5G66150/Other gl</td><td>LOC_Os10g05069.1|13110.m00422|protein/1E-101/Other glycan d</td><td>0.31</td><td>0</td><td>0.4</td><td>0</td><td>0.79</td><td>0.507938665</td></tr>
<tr><td>AP13CTG12778_at</td><td>AP13CTG12778</td><td>smart00579/domain in F</td><td>/</td><td>/</td><td>LOC_Os07g36530.1|13107.m03751|protein/0.00000000000001//</td><td>0.32</td><td>1.81E-13</td><td>0.33</td><td>2.39E-25</td><td>0.95</td><td>0.811964344</td></tr>
<tr><td>AP13ITG40821_s_at</td><td>AP13ISTG40821</td><td>cd01837/SGNH_plant_lip</td><td>LATERAL SIGNALING TARGE</td><td>/</td><td>LOC_Os01g42730.1|13101.m04398|protein/2E-77//</td><td>0.32</td><td>0</td><td>0.23</td><td>0</td><td>1.41</td><td>0.07868336</td></tr>
<tr><td>AP13ITG54974_at</td><td>AP13ISTG54974</td><td>pfam04770/ZF-HD prote</td><td>/</td><td>/</td><td>LOC_Os11g03420.1|13111.m00281|protein/9E-30//</td><td>0.32</td><td>1.42E-41</td><td>0.33</td><td>6.67E-144</td><td>0.97</td><td>0.74224083</td></tr>
<tr><td>AP13ITG72069-RC_at</td><td></td><td></td><td></td><td></td><td></td><td>0.32</td><td>3.80E-69</td><td>0.18</td><td>4.98E-88</td><td>1.71</td><td>0.00075455</td></tr>
<tr><td>KanlowCTG00595-3_s_at</td><td></td><td></td><td></td><td></td><td></td><td>0.32</td><td>0</td><td>0.34</td><td>0</td><td>0.95</td><td>0.829507648</td></tr>
<tr><td>KanlowCTG17757_at</td><td>KanlCTG17757</td><td>//</td><td>/</td><td>/</td><td>///</td><td>0.32</td><td>3.75E-20</td><td>0.24</td><td>3.59E-34</td><td>1.34</td><td>0.194075687</td></tr>
<tr><td>OTHSWCTG15634_s_a</td><td>OthsCTG15634</td><td>smart00547/Zinc finger c</td><td>FAMILY NOT NAMED/0.000</td><td>/</td><td>LOC_Os01g37460.1|13101.m03808|protein/0.0000000000000004/</td><td>0.32</td><td>4.18E-10</td><td>0.28</td><td>6.09E-13</td><td>1.15</td><td>0.686234986</td></tr>
<tr><td>AP13ITG54401_at</td><td>AP13ISTG54401</td><td>PTZ00009/heat shock 70</td><td>FAMILY NOT NAMED/0</td><td>AT3G12580/Spliceos</td><td>LOC_Os03g16920.1|13103.m02017|protein/0/Spliceosome; Protein</td><td>0.33</td><td>1.42E-08</td><td>0.21</td><td>1.84E-11</td><td>1.59</td><td>0.375514401</td></tr>
<tr><td>AP13ITG63880RC_at</td><td>AP13ISTG63880-RC</td><td>smart00380/DNA-bindin</td><td>PREDICTED PROTEIN/0.000</td><td>/</td><td>LOC_Os02g42585.1|13102.m12308|protein/2E-103//</td><td>0.33</td><td>7.76E-21</td><td>0.36</td><td>9.47E-14</td><td>0.93</td><td>0.708730872</td></tr>
<tr><td>AP13ITG64473_at</td><td>AP13ISTG64473</td><td>pfam02956/TT viral orf 1</td><td>OS10G0116300 PROTEIN/9</td><td>/</td><td>LOC_Os09g30490.1|13109.m03059|protein/1E-27//</td><td>0.33</td><td>0</td><td>0.39</td><td>0</td><td>0.83</td><td>0.584081397</td></tr>
<tr><td>OTHSWCTG17935_s_a</td><td>OthsCTG17935</td><td>pfam10184/Uncharacter</td><td>/</td><td>/</td><td>LOC_Os07g10495.1|13107.m01067|protein/3E-60//</td><td>0.33</td><td>0</td><td>0.39</td><td>0</td><td>0.83</td><td>0.419418977</td></tr>
<tr><td>OTHSWCTG18646_at</td><td>OthsCTG18646</td><td>//</td><td>/</td><td>/</td><td>///</td><td>0.33</td><td>1.08E-200</td><td>0.34</td><td>8.82E-22</td><td>0.95</td><td>0.487895557</td></tr>
<tr><td>AP13CTG11801_at</td><td>AP13CTG11801</td><td>smart00220/Serine/Thre</td><td>SUBFAMILY NOT NAMED/6</td><td>/</td><td>LOC_Os01g10890.1|13101.m01175|protein/2E-178//</td><td>0.34</td><td>2.77E-09</td><td>0.33</td><td>4.53E-17</td><td>1.03</td><td>0.888903341</td></tr>
<tr><td>AP13ITG43189_s_at</td><td>AP13ISTG43189</td><td>pfam02469/Fasciclin dor</td><td>FLA (FASCICLIN-LIKE ARABII</td><td>/</td><td>LOC_Os09g30486.1|13109.m03058|protein/4E-70//</td><td>0.34</td><td>4.42E-14</td><td>0.29</td><td>3.37E-45</td><td>1.19</td><td>0.283385213</td></tr>
<tr><td>KanlowCTG19494_s_a</td><td>KanlCTG19494</td><td>//</td><td>/</td><td>/</td><td>///</td><td>0.34</td><td>1.31E-16</td><td>0.3</td><td>9.90E-21</td><td>1.16</td><td>0.537310286</td></tr>
<tr><td>KanlowCTG28988_at</td><td>KanlCTG28988</td><td>pfam00856/SET domain.</td><td>SET DOMAIN PROTEIN/0.0(</td><td>/</td><td>LOC_Os02g03030.1|13102.m00269|protein/3E-63//</td><td>0.34</td><td>1.67E-220</td><td>0.36</td><td>0</td><td>0.94</td><td>0.003909875</td></tr>
<tr><td>KanlowCTG44452_s_a</td><td>KanlCTG44452</td><td>pfam05667/Protein of ur</td><td>/</td><td>/</td><td>LOC_Os02g14500.1|13102.m01613|protein/1E-21//</td><td>0.34</td><td>8.17E-12</td><td>0.33</td><td>1.24E-15</td><td>1.02</td><td>0.94051114</td></tr>
<tr><td>VS16ITG20386_s_at</td><td>VS16ISTG20386</td><td>//</td><td>/</td><td>/</td><td>LOC_Os07g47450.1|13107.m05050|protein/0.000000000000002//</td><td>0.34</td><td>9.29E-32</td><td>0.34</td><td>1.10E-66</td><td>0.99</td><td>0.915774362</td></tr>
<tr><td>AP13CTG10505_at</td><td>AP13CTG10505</td><td>TIGR00393/KpsF/GutQ fa</td><td>CAPSULE EXPRESSION PRO</td><td>/</td><td>LOC_Os02g06360.1|13102.m00699|protein/5E-171//</td><td>0.35</td><td>7.57E-37</td><td>0.33</td><td>6.88E-21</td><td>1.07</td><td>0.661488353</td></tr>
<tr><td>AP13ITG56710_s_at</td><td>AP13ISTG56710</td><td>PLN02580/trehalose-pho</td><td>TREHALOSE-6-PHOSPHATE</td><td>/</td><td>LOC_Os02g44230.3|13102.m04935|protein/1E-144//</td><td>0.35</td><td>1.62E-08</td><td>0.34</td><td>9.75E-181</td><td>1.03</td><td>0.673991551</td></tr>
<tr><td>AP13ITG60355_at</td><td>AP13ISTG60355</td><td>PLN02555/limonoid gluc</td><td>UDP-GLUCOSYLTRANSFERA</td><td>AT1G22400/Zeatin b</td><td>LOC_Os04g37820.1|13104.m03621|protein/0//</td><td>0.35</td><td>1.08E-15</td><td>0.26</td><td>0</td><td>1.31</td><td>0.195168916</td></tr>
<tr><td>AP13ITG67617_s_at</td><td>AP13ISTG67617</td><td>PRK04537/ATP-depende</td><td>/</td><td>/</td><td>LOC_Os11g27540.2|13111.m02640|protein/8E-40//</td><td>0.35</td><td>1.33E-14</td><td>0.4</td><td>1.13E-23</td><td>0.88</td><td>0.446771462</td></tr>
<tr><td>KanlowCTG17315_s_a</td><td>KanlCTG17315</td><td>pfam01843/DIL domain.</td><td>SUBFAMILY NOT NAMED/1</td><td>/</td><td>LOC_Os03g53660.1|13103.m05865|protein/1E-38//</td><td>0.35</td><td>5.30E-261</td><td>0.37</td><td>1.44E-27</td><td>0.95</td><td>0.320669323</td></tr>
<tr><td>KanlowCTG41891_s_a</td><td>KanlCTG41891</td><td>pfam01357/Pollen allerg</td><td>FAMILY NOT NAMED/0.000</td><td>/</td><td>LOC_Os10g40720.1|13110.m03775|protein/2E-39//</td><td>0.35</td><td>1.73E-16</td><td>0.39</td><td>9.99E-16</td><td>0.91</td><td>0.651081736</td></tr>
<tr><td>AlamoCTG12892_s_at</td><td>AlamCTG12892</td><td>smart00219/Tyrosine kir</td><td>SUBFAMILY NOT NAMED/6</td><td>/</td><td>LOC_Os01g21970.1|13101.m02335|protein/6E-75//</td><td>0.36</td><td>7.33E-09</td><td>0.39</td><td>2.63E-08</td><td>0.94</td><td>0.844390777</td></tr>
<tr><td>AP13CTG01671_at</td><td>AP13CTG01671</td><td>PLN00221/tubulin, alpha</td><td>FAMILY NOT NAMED/0</td><td>AT4G14960/Phagosc</td><td>LOC_Os07g38730.1|13107.m04008|protein/0/Phagosome/0</td><td>0.36</td><td>2.15E-32</td><td>0.39</td><td>3.21E-22</td><td>0.91</td><td>0.51677089</td></tr>
<tr><td>AP13ITG38936_s_at</td><td>AP13ISTG38936</td><td>PHA03247/large tegume</td><td>SUBFAMILY NOT NAMED/4</td><td>/</td><td>LOC_Os03g04710.1|13103.m00491|protein/7E-108//</td><td>0.36</td><td>1.27E-25</td><td>0.27</td><td>3.30E-36</td><td>1.36</td><td>0.099282184</td></tr>
<tr><td>AP13ITG65721_at</td><td>AP13ISTG65721</td><td>//</td><td>/</td><td>/</td><td>///</td><td>0.36</td><td>1.78E-11</td><td>0.33</td><td>6.49E-15</td><td>1.08</td><td>0.754919161</td></tr>
<tr><td>AP13ITG70020_at</td><td>AP13ISTG70020</td><td>COG0484/DnaJ-class mo</td><td>OS05G0427900 PROTEIN/3</td><td>/</td><td>LOC_Os01g65480.1|13101.m07075|protein/4E-106//</td><td>0.36</td><td>3.14E-58</td><td>0.36</td><td>3.58E-246</td><td>0.99</td><td>0.916709498</td></tr>
<tr><td>KanlowCTG09985_s_a</td><td>KanlCTG09985</td><td>PRK10811/ribonuclease</td><td>/</td><td>/</td><td>///</td><td>0.36</td><td>5.42E-13</td><td>0.39</td><td>2.16E-20</td><td>0.92</td><td>0.650705217</td></tr>
<tr><td>KanlowCTG16242_s_a</td><td>KanlCTG16242</td><td>TIGR01833/3-hydroxy-3-</td><td>SUBFAMILY NOT NAMED/2</td><td>AT4G11820/Synthes</td><td>LOC_Os03g02710.1|13103.m00242|protein/1E-112/Synthesis and (</td><td>0.36</td><td>5.73E-13</td><td>0.4</td><td>5.02E-21</td><td>0.9</td><td>0.533177522</td></tr>
<tr><td>KanlowCTG19450_s_a</td><td>KanlCTG19450</td><td>PLN00191/enolase/0</td><td>FAMILY NOT NAMED/1.6E-</td><td>AT2G36530/Glycoly</td><td>LOC_Os06g04510.1|13106.m00409|protein/0/Glycolysis / Glucone</td><td>0.36</td><td>0</td><td>0.4</td><td>0</td><td>0.89</td><td>0.341308102</td></tr>
<tr><td>KanlowCTG20720_s_a</td><td>KanlCTG20720</td><td>cd05032/Catalytic doma</td><td>ANKYRIN-KINASE/2.4E-34</td><td>/</td><td>LOC_Os01g66860.3|13101.m07243|protein/3E-50//</td><td>0.36</td><td>0</td><td>0.37</td><td>0</td><td>0.98</td><td>0.484279147</td></tr>
<tr><td>OTHSWCTG11406_s_a</td><td>OthsCTG11406</td><td>//</td><td>/</td><td>/</td><td>///</td><td>0.36</td><td>0</td><td>0.3</td><td>0</td><td>1.2</td><td>0.160579056</td></tr>
<tr><td>AP13CTG11412_at</td><td>AP13CTG11412</td><td>pfam06027/Eukaryotic p</td><td>DUF914/ANTHOCYANIN MI</td><td>/</td><td>LOC_Os08g42720.1|13108.m04615|protein/1E-147//</td><td>0.37</td><td>0</td><td>0.29</td><td>0</td><td>1.26</td><td>0.153398673</td></tr>
<tr><td>AP13CTG15858_at</td><td>AP13CTG15858</td><td>PLN02766/coniferyl-alde</td><td>ALDEHYDE DEHYDROGENA:</td><td>AT3G24503/Phenylp</td><td>LOC_Os01g40860.1|13101.m04187|protein/0/Phenylpropanoid bic</td><td>0.37</td><td>0</td><td>0.37</td><td>0</td><td>1</td><td>0.996484564</td></tr>
<tr><td>AP13CTG24367_at</td><td>AP13CTG24367</td><td>pfam07522/DNA repair r</td><td>FAMILY NOT NAMED/9.7E-</td><td>/</td><td>LOC_Os01g57440.1|13101.m06094|protein/1E-164//</td><td>0.37</td><td>0</td><td>0.36</td><td>0</td><td>1.05</td><td>0.815039518</td></tr>
<tr><td>AP13CTG24945_at</td><td>AP13CTG24945</td><td>pfam01466/Skp1 family,</td><td>SKP1/5.9E-60</td><td>AT4G34210/Ubiquiti</td><td>LOC_Os07g43180.1|13107.m04538|protein/9E-26/Ubiquitin media</td><td>0.37</td><td>0</td><td>0.39</td><td>1.30E-42</td><td>0.95</td><td>0.036886728</td></tr>
<tr><td>AP13ITG65828_s_at</td><td>AP13ISTG65828</td><td>pfam06376/Protein of ur</td><td>/</td><td>/</td><td>LOC_Os02g16500.1|13102.m01845|protein/0.00000000000009//</td><td>0.37</td><td>0</td><td>0.28</td><td>0</td><td>1.33</td><td>0.319070164</td></tr>
<tr><td>KanlowCTG21445_s_a</td><td>KanlCTG21445</td><td>pfam00249/Myb-like DN</td><td>MYB DNA BINDING / TRANS</td><td>/</td><td>LOC_Os05g04820.1|13105.m00495|protein/6E-135//</td><td>0.37</td><td>5.44E-08</td><td>0.22</td><td>8.42E-83</td><td>1.66</td><td>0.000223093</td></tr>
<tr><td>KanlowCTG44049_at</td><td>KanlCTG44049</td><td>//</td><td>/</td><td>/</td><td>LOC_Os04g51090.1|13104.m05196|protein/7E-99//</td><td>0.37</td><td>2.20E-26</td><td>0.34</td><td>2.07E-67</td><td>1.08</td><td>0.474559549</td></tr>
<tr><td>AlamoCTG07438_at</td><td>AlamCTG07438</td><td>PHA03245/large tegume</td><td>/</td><td>/</td><td>LOC_Os01g62970.2|13101.m06755|protein/1E-29//</td><td>0.38</td><td>4.44E-16</td><td>0.4</td><td>2.44E-15</td><td>0.96</td><td>0.865124985</td></tr>
<tr><td>AlamoCTG12062_s_at</td><td>AlamCTG12062</td><td>//</td><td>/</td><td>/</td><td>LOC_Os01g62970.2|13101.m06755|protein/0.003//</td><td>0.38</td><td>0</td><td>0.37</td><td>0</td><td>1.02</td><td>0.884124667</td></tr>
<tr><td>AP13CTG11451_s_at</td><td>AP13CTG11451</td><td>PLN02870/polygalacturo</td><td>SUBFAMILY NOT NAMED/7</td><td>AT5G15470/Starch a</td><td>LOC_Os03g11330.1|13103.m01277|protein/0/Starch and sucrose n</td><td>0.38</td><td>0</td><td>0.31</td><td>3.50E-31</td><td>1.24</td><td>0</td></tr>
<tr><td>AP13CTG22184RC_at</td><td>AP13CTG22184-RC</td><td>pfam01843/DIL domain.</td><td>MYOSIN XI/1.8E-88</td><td>/</td><td>LOC_Os03g53660.1|13103.m05865|protein/3E-112//</td><td>0.38</td><td>4.28E-74</td><td>0.35</td><td>4.83E-97</td><td>1.08</td><td>0.380261259</td></tr>
<tr><td>AP13CTG02233_s_at</td><td>AP13CTG02233</td><td>//</td><td>F-BOX FAMILY PROTEIN/1.(</td><td>/</td><td>LOC_Os01g47050.1|13101.m04843|protein/0//</td><td>0.39</td><td>1.80E-77</td><td>0.29</td><td>0</td><td>1.33</td><td>2.12E-08</td></tr>
<tr><td>AP13CTG02287_s_at</td><td>AP13CTG02287</td><td>TIGR01833/3-hydroxy-3-</td><td>SUBFAMILY NOT NAMED/1</td><td>AT4G11820/Synthes</td><td>LOC_Os03g02710.1|13103.m00242|protein/0/Synthesis and degrac</td><td>0.39</td><td>9.82E-29</td><td>0.38</td><td>1.49E-54</td><td>1.02</td><td>0.817694261</td></tr>
<tr><td>AP13CTG30517_at</td><td>AP13CTG30517</td><td>//</td><td>/</td><td>/</td><td>///</td><td>0.39</td><td>1.60E-12</td><td>0.35</td><td>1.76E-67</td><td>1.1</td><td>0.328181502</td></tr>
<tr><td>AP13ITG41401_at</td><td>AP13ISTG41401</td><td>PHA03247/large tegume</td><td>/</td><td>/</td><td>LOC_Os01g07240.1|13101.m00724|protein/8E-20//</td><td>0.39</td><td>2.92E-36</td><td>0.35</td><td>7.26E-31</td><td>1.11</td><td>0.426551698</td></tr>
<tr><td>AP13ITG77659_at</td><td>AP13ISTG77659</td><td>pfam04043/Plant inverta</td><td>/</td><td>/</td><td>LOC_Os10g36500.2|13110.m07835|protein/6E-23//</td><td>0.39</td><td>0</td><td>0.16</td><td>0</td><td>2.47</td><td>0.0001787</td></tr>
<tr><td>KanlowCTG03398_at</td><td>KanlCTG03398</td><td>//</td><td>FAMILY NOT NAMED/1E-17</td><td>/</td><td>LOC_Os01g02900.2|13101.m00231|protein/1E-173//</td><td>0.39</td><td>9.42E-11</td><td>0.32</td><td>6.21E-16</td><td>1.24</td><td>0.374596673</td></tr>
<tr><td>AP13CTG13387_at</td><td>AP13CTG13387</td><td>PLN02203/aldehyde deh</td><td>ALDEHYDE DEHYDROGENA:</td><td>AT4G36250/Glycoly</td><td>LOC_Os02g43194.1|13102.m04795|protein/0/Glycolysis / Glucone</td><td>0.4</td><td>4.86E-09</td><td>0.36</td><td>5.45E-28</td><td>1.1</td><td>0.537334558</td></tr>
<tr><td>KanlowCTG13385_s_a</td><td>KanlCTG13385</td><td>cd01812/Ubiquitin-like c</td><td>OS09G0524800 PROTEIN/4</td><td>/</td><td>LOC_Os06g03640.1|13106.m00300|protein/8E-81//</td><td>0.4</td><td>0</td><td>0.35</td><td>0</td><td>1.13</td><td>0.437739097</td></tr>
<tr><td>KanlowCTG31345_at</td><td>KanlCTG31345</td><td>PRK12323/DNA polymer</td><td>FAMILY NOT NAMED/5.3E-</td><td>/</td><td>LOC_Os01g37460.1|13101.m03808|protein/2E-61//</td><td>0.4</td><td>6.55E-38</td><td>0.4</td><td>2.63E-225</td><td>1</td><td>0.96664998</td></tr>
<tr><td>KanlowCTG47138_s_a</td><td>KanlCTG47138</td><td>pfam01419/Jacalin-like l</td><td>JASMONATE INDUCIBLE PR</td><td>/</td><td>LOC_Os05g43240.3|13105.m04549|protein/8E-108//</td><td>0.4</td><td>1.03E-09</td><td>0.35</td><td>2.18E-11</td><td>1.12</td><td>0.650503419</td></tr>
</table>

The transcript ratios of targeted MTHFR and *COMT* genes were listed below.

<table>
<tr><th></th><th></th><th></th><th></th><th></th><th></th><th></th><th></th><th></th><th></th><th></th><th></th></tr>
<tr><td>AP13CTG11172_s_at</td><td>AP13CTG11172</td><td>//</td><td>METHYLENETETRAHYDROF</td><td>AT3G59970/One car</td><td>LOC_Os03g60090.1|13103.m06595|protein/7E-101/One carbon po</td><td>0.44</td><td>1.28116E-22</td><td>0.64</td><td>1.13934E-09</td><td>0.69</td><td>0.000604736</td></tr>
<tr><td>KanlowCTG20791_s_a</td><td>KanlCTG20791</td><td>pfam00891/O-methyltra</td><td>FAMILY NOT NAMED/8.9E-</td><td>AT5G54160/Flavone</td><td>LOC_Os08g06100.1|13108.m00605|protein/1E-75/Flavone and flav</td><td>0.18</td><td>1.4477E-290</td><td>0.76</td><td>0.029673022</td><td>0.23</td><td>3.42E-146</td></tr>
</table>

Table S5. List of significantly DEGs in different pathways in four comparisons

List of significantly DEGs involved in hormone metabolism and development pathway in Exp/Ctrl comparison

Hormone

BinCode	BinName	id	type	description	Exp/Ctrl (log2)
17.6.1.1	hormone metabolism.gibberelin.synthesis-degradation.copalyl diphosp	othswctg09951_s_at	Transcript	very weakly similar to (87.8) AT4G02780 | Symbols: GA1, ABC33, ATCPS1 | GA1 (GA REQUIRING 1); ent-copalyl diphosphate synthase/ magnesium ion binding | ch	2.283089
17.6.1.1	hormone metabolism.gibberelin.synthesis-degradation.copalyl diphosp	ap13itg72235_s_at	Transcript	weakly similar to (197) loc_os04g09900 12004.m35118 protein ent-kaurene synthase A, chloroplast precursor, putative, expressed no original description	2.5106273
17.7.1.5	hormone metabolism.jasmonate.synthesis-degradation.12-Oxo-PDA-re	kanlowctg23007_at	Transcript	weakly similar to (176) AT1G76690 | Symbols: OPR2, ATOPR2 | OPR2 | chr1:28778976-28780355 FORWARDmoderately similar to (241) loc_os06g11210 12006.m	4.7242365
17.7.1.5	hormone metabolism.jasmonate.synthesis-degradation.12-Oxo-PDA-re	ap13itg71073_s_at	Transcript	weakly similar to (110) AT1G76690 | Symbols: OPR2, ATOPR2 | OPR2 | chr1:28778976-28780355 FORWARDweakly similar to (167) loc_os06g11210 12006.m058	3.2742903
17.7.1.5	hormone metabolism.jasmonate.synthesis-degradation.12-Oxo-PDA-re	ap13itg67010_s_at	Transcript	weakly similar to (115) AT1G76680 | Symbols: OPR1, ATOPR1 | OPR1; 12-oxophytodienoate reductase | chr1:28776982-28778271 FORWARDweakly similar to (1	2.168549

Development

BinCode	BinName	id	type	description	Exp/Ctrl (log2)
33.99	development.unspecified	ap13itg63590_at	Transcript	weakly similar to (198) loc_os01g51870 12001.m11363 protein S-adenosylmethionine-dependent methyltransferase, putative, expressed no original description	2.8097055
33.99	development.unspecified	ap13itg57467-rc_s_at	Transcript	very weakly similar to (88.6) AT5G48850 | Symbols: ATSDI1 | ATSDI1 (SULPHUR DEFICIENCY-INDUCED 1); binding | chr5:19805576-19807699 REVERSEweakly simil	1.9713516
33.99	development.unspecified	ap13itg76686_at	Transcript	weakly similar to (151) AT5G48850 | Symbols: ATSDI1 | ATSDI1 (SULPHUR DEFICIENCY-INDUCED 1); binding | chr5:19805576-19807699 REVERSEmoderately simil	1.8074428

List of significantly DEGs involved in signalling, hormone, cell and transport pathway in Mo/60 comparison

Signalling

BinCode	BinName	id	type	description	Mo/60 (log2)
30.1	signalling.in sugar and nutrient physiology	ap13itg74992_at	Transcript	weakly similar to (117) loc_os02g54640 12002.m10493 protein glutamate receptor 2.9 precursor, putative, expressed no original description	1.119977
30.2.11	signalling.receptor kinases.leucine rich repeat XI	ap13itg61870_at	Transcript	weakly similar to (116) loc_os11g07240 12011.m04920 protein receptor protein kinase CLAVATA1 precursor, putative, expressed no original description	1.1316558
30.2.11	signalling.receptor kinases.leucine rich repeat XI	othswctg12119_at	Transcript	very weakly similar to (80.1) AT3G47580 | Symbols: | leucine-rich repeat transmembrane protein kinase, putative | chr3:17532687-17535810 FORWARDweakly sir	1.0568756
30.2.11	signalling.receptor kinases.leucine rich repeat XI	othswctg27954_s_at	Transcript	weakly similar to (132) loc_os02g40180 12002.m09104 protein receptor-like protein kinase precursor, putative, expressed no original description	2.0184202
30.2.11	signalling.receptor kinases.leucine rich repeat XI	ap13ctg04179_at	Transcript	weakly similar to (105) loc_os01g65650 12001.m12674 protein receptor-like protein kinase 5 precursor, putative, expressed no original description	-1.9957428
30.2.11	signalling.receptor kinases.leucine rich repeat XI	kanlowctg42715_at	Transcript	weakly similar to (107) AT3G47570 | Symbols: | leucine-rich repeat transmembrane protein kinase, putative | chr3:17527611-17530748 FORWARDmoderately sin	2.03235
30.2.11	signalling.receptor kinases.leucine rich repeat XI	othswctg19246_at	Transcript	very weakly similar to (84.0) AT3G47110 | Symbols: | leucine-rich repeat transmembrane protein kinase, putative | chr3:17347103-17350296 REVERSEweakly simi	1.51942
30.2.11	signalling.receptor kinases.leucine rich repeat XI	ap13ctg16678_s_at	Transcript	very weakly similar to (81.3) AT5G48380 | Symbols: | leucine-rich repeat family protein / protein kinase family protein | chr5:19604584-19606532 REVERSEweakly	1.0412167
30.2.11	signalling.receptor kinases.leucine rich repeat XI	othswctg17938_at	Transcript	weakly similar to (115) AT2G15080 | Symbols: AtRLP19 | AtRLP19 (Receptor Like Protein 19); kinase/ protein binding | chr2:6533764-6536715 FORWARDweakly s	1.6272969
30.2.11	signalling.receptor kinases.leucine rich repeat XI	othswctg17630_at	Transcript	weakly similar to (174) loc_os02g40240 12002.m33414 protein leucine-rich repeat receptor protein kinase EXS precursor, putative, expressed no original descripti	1.6988038
30.2.11	signalling.receptor kinases.leucine rich repeat XI	ap13ctg50688_s_at	Transcript	weakly similar to (105) loc_os04g15630 12004.m06749 protein leucine-rich repeat receptor protein kinase EXS precursor, putative no original description	1.5420277
30.2.11	signalling.receptor kinases.leucine rich repeat XI	kanlowctg19800_s_at	Transcript	very weakly similar to (90.9) loc_os02g40180 12002.m09104 protein receptor-like protein kinase precursor, putative, expressed no original description	1.3183337
30.2.11	signalling.receptor kinases.leucine rich repeat XI	othswslt25490_at	Transcript	weakly similar to (114) AT3G47570 | Symbols: | leucine-rich repeat transmembrane protein kinase, putative | chr3:17527611-17530748 FORWARDmoderately sin	1.2138089
30.2.11	signalling.receptor kinases.leucine rich repeat XI	ap13ctg03762_at	Transcript	weakly similar to (181) loc_os02g40240 12002.m33414 protein leucine-rich repeat receptor protein kinase EXS precursor, putative, expressed no original descripti	1.5610776
30.2.11	signalling.receptor kinases.leucine rich repeat XI	othswctg18100_at	Transcript	weakly similar to (131) loc_os11g36190 12011.m080025 protein receptor-like protein kinase 5 precursor, putative, expressed no original description	1.3126502
30.2.16	signalling.receptor kinases.Catharanthus roseus-like RLK1	ap13ctg04617_at	Transcript	weakly similar to (143) loc_os06g22810 12006.m06934 protein protein kinase, putative, expressed no original description	-1.1393493
30.2.17	signalling.receptor kinases.DUF 26	ap13itg41137_at	Transcript	weakly similar to (142) loc_os09g39650 12009.m060077 protein ATP binding protein, putative, expressed no original description	2.0351868
30.2.17	signalling.receptor kinases.DUF 26	othswctg18108_at	Transcript	very weakly similar to (97.8) AT1G11330 | Symbols: | S-locus lectin protein kinase family protein | chr1:3810372-3813416 FORWARDweakly similar to (148) loc_o	1.9088267
30.2.17	signalling.receptor kinases.DUF 26	ap13itg69009_at	Transcript	very weakly similar to (90.1) AT4G23160 | Symbols: | protein kinase family protein | chr4:12129485-12134086 FORWARDweakly similar to (166) loc_os05g39130	1.1350458
30.2.17	signalling.receptor kinases.DUF 26	ap13itg77404_s_at	Transcript	very weakly similar to (94.7) AT4G03230 | Symbols: | ATP binding / kinase/ protein kinase/ protein serine/threonine kinase/ protein tyrosine kinase/ sugar binding	1.5196985
30.2.17	signalling.receptor kinases.DUF 26	ap13itg39900_s_at	Transcript	very weakly similar to (91.3) loc_os12g14480 12012.m05416 protein ATP binding protein, putative no original description	2.5227633
30.2.17	signalling.receptor kinases.DUF 26	ap13itg76323_at	Transcript	weakly similar to (116) loc_os01g02390 12001.m42838 protein TAK14, putative, expressed no original description	2.8866818
30.2.17	signalling.receptor kinases.DUF 26	ap13itg47888_s_at	Transcript	very weakly similar to (86.7) loc_os11g11780 12011.m05370 protein serine/threonine protein kinase, putative, expressed no original description	-1.1535704
30.2.17	signalling.receptor kinases.DUF 26	ap13ctg07687_at	Transcript	weakly similar to (159) loc_os01g02800 12001.m06920 protein receptor-like kinase ARK1AS, putative, expressed no original description	1.2190235
30.2.17	signalling.receptor kinases.DUF 26	ap13ctg48706_at	Transcript	very weakly similar to (98.6) AT4G23130 | Symbols: CRK5, RLK6 | CRK5 (CYSTEINE-RICH RLK5); kinase | chr4:12117688-12120134 REVERSEvery weakly similar to (8	3.9346542
30.2.17	signalling.receptor kinases.DUF 26	ap13ctg11071_s_at	Transcript	weakly similar to (102) loc_os07g35290 12007.m07785 protein receptor-like serine-threonine protein kinase, putative, expressed no original description	1.346304
30.2.17	signalling.receptor kinases.DUF 26	ap13ctg22926_at	Transcript	very weakly similar to (90.5) AT4G23180 | Symbols: CRK10, RLK4 | CRK10 (CYSTEINE-RICH RLK10); ATP binding / kinase/ protein kinase/ protein serine/threonine k	-1.065511
30.2.25	signalling.receptor kinases.wall associated kinase	ap13ctg19859_at	Transcript	weakly similar to (142) loc_os04g43730 12004.m09332 protein OsWAK51 - OsWAK receptor-like protein kinase, expressed no original description	1.6219307
30.2.25	signalling.receptor kinases.wall associated kinase	ap13ctg14697_s_at	Transcript	weakly similar to (162) loc_os04g03830 12004.m05691 protein OsWAK29 - OsWAK receptor-like protein kinase, expressed no original description	1.7394327
30.2.25	signalling.receptor kinases.wall associated kinase	othswctg14972_at	Transcript	weakly similar to (130) loc_os02g42150 12002.m09252 protein OsWAK14 - OsWAK receptor-like protein kinase, expressed no original description	3.268809
30.2.25	signalling.receptor kinases.wall associated kinase	alamoctg14172_at	Transcript	very weakly similar to (88.6) loc_os02g42150 12002.m09252 protein OsWAK14 - OsWAK receptor-like protein kinase, expressed no original description	1.109805
30.2.25	signalling.receptor kinases.wall associated kinase	ap13ctg23391_at	Transcript	weakly similar to (116) loc_os03g62430 12003.m11119 protein OsWAK28 - OsWAK receptor-like protein kinase, expressed no original description	1.8729477
30.2.25	signalling.receptor kinases.wall associated kinase	ap13itg48098_at	Transcript	very weakly similar to (94.4) AT1G21230 | Symbols: WAK5 | WAK5 (WALL ASSOCIATED KINASE 5); kinase/ protein serine/threonine kinase | chr1:7429980-743234	-1.1851537
30.2.99	signalling.receptor kinases.misc	ap13itg77449-rc_at	Transcript	weakly similar to (138) loc_os05g44970 12005.m08622 protein senescence-induced receptor-like serine/threonine-protein kinase precursor, putative no original c	1.7617186
30.2.99	signalling.receptor kinases.misc	ap13itg41137_at	Transcript	weakly similar to (142) loc_os09g39650 12009.m060077 protein ATP binding protein, putative, expressed no original description	2.0351868
30.2.99	signalling.receptor kinases.misc	kanlowctg38494_at	Transcript	very weakly similar to (95.9) AT2G28970 | Symbols: | leucine-rich repeat protein kinase, putative | chr2:12443919-12448163 FORWARDweakly similar to (165) loc	1.0940689
30.2.99	signalling.receptor kinases.misc	ap13itg58665_at	Transcript	weakly similar to (132) AT2G28970 | Symbols: | leucine-rich repeat protein kinase, putative | chr2:12443919-12448163 FORWARDweakly similar to (105) NORK_I	1.2050234
30.2.99	signalling.receptor kinases.misc	ap13itg48098_at	Transcript	very weakly similar to (94.4) AT1G21230 | Symbols: WAK5 | WAK5 (WALL ASSOCIATED KINASE 5); kinase/ protein serine/threonine kinase | chr1:7429980-743234	-1.1851537
30.3	signalling.calcium	ap13itg66751_s_at	Transcript	weakly similar to (117) AT2G18750 | Symbols: | calmodulin-binding protein | chr2:8125827-8128363 FORWARDmoderately similar to (220) loc_os12g36920 1201	1.5560796
30.3	signalling.calcium	kanlowctg04099_s_at	Transcript	weakly similar to (104) AT1G27770 | Symbols: ACA1, PEA1 | ACA1 (AUTO-INHIBITED CA2+-ATPASE 1); calcium channel/ calcium-transporting ATPase/ calmodulin b	1.0539427
30.3	signalling.calcium	ap13ctg14744_s_at	Transcript	weakly similar to (128) loc_os12g36110 12012.m07401 protein calmodulin binding protein, putative, expressed no original description	2.738117
30.3	signalling.calcium	ap13ctg08973_s_at	Transcript	very weakly similar to (81.6) AT4G37640 | Symbols: ACA2 | ACA2 (CALCIUM ATPASE 2); calcium ion transmembrane transporter/ calcium-transporting ATPase/ cal	1.1298444
30.5	signalling.G-proteins	kanlowctg44370_s_at	Transcript	very weakly similar to (91.3) loc_os06g02130 12006.m31954 protein guanyl nucleotide binding protein, putative, expressed no original description	1.6158048
30.1	signalling.phosphorelay	ap13ctg09520_at	Transcript	very weakly similar to (100) AT3G16360 | Symbols: AHP4 | AHP4 (HPT PHOSPHOTRANSMITTER 4); histidine phosphotransfer kinase/ transferase, transferring pho:	-1.4311115
30.11.1	signalling.light.COP9 signalosome	ap13ctg07211_at	Transcript	very weakly similar to (96.7) loc_os07g26440 12007.m079669 protein CIP7, putative, expressed no original description	-1.7148267

Hormone

BinCode	BinName	id	type	description	Mo/60 (log2)
17.2.3	hormone metabolism.auxin.induced-regulated-responsive-activated	ap13itg71573-rc_at	Transcript	no original description	1.2041686
17.2.3	hormone metabolism.auxin.induced-regulated-responsive-activated	ap13itg62309_at	Transcript	weakly similar to (172) AT1G60730 | Symbols: | aldo/keto reductase family protein | chr1:22358613-22360082 REVERSEweakly similar to (115) A115_TOBAC Aux	2.3817716
17.2.3	hormone metabolism.auxin.induced-regulated-responsive-activated	ap13itg58736_s_at	Transcript	very weakly similar to (85.5) loc_os09g37430 12009.m06690 protein OsSAUR48 - Auxin-responsive SAUR gene family member no original description	-1.8851447
17.2.3	hormone metabolism.auxin.induced-regulated-responsive-activated	ap13itg42880-rc_at	Transcript	weakly similar to (114) loc_os01g67010 12001.m12806 protein expressed protein no original description	-1.200312
17.2.3	hormone metabolism.auxin.induced-regulated-responsive-activated	ap13itg70591-rc_at	Transcript	weakly similar to (111) loc_os04g51890 12004.m10084 protein OsSAUR20 - Auxin-responsive SAUR gene family member, expressed no original description	2.8087711
17.5.1	hormone metabolism.ethylene.synthesis-degradation	ap13ctg10284_at	Transcript	very weakly similar to (85.1) loc_os08g32170 12008.m07211 protein oxidoreductase, putative, expressed no original description	-1.4918615
17.6.1.1	hormone metabolism.gibberelin.synthesis-degradation.copalyl diphos	othswctg09951_s_at	Transcript	very weakly similar to (87.8) AT4G02780 | Symbols: GA1, ABC33, ATCPS1 | GA1 (GA REQUIRING 1); ent-copalyl diphosphate synthase/ magnesium ion binding | ch	2.7778554
17.6.1.1	hormone metabolism.gibberelin.synthesis-degradation.copalyl diphos	ap13itg72235_s_at	Transcript	weakly similar to (197) loc_os04g09900 12004.m35118 protein ent-kaurene synthase A, chloroplast precursor, putative, expressed no original description	3.394579
17.7.1.2	hormone metabolism.jasmonate.synthesis-degradation.lipoxygenase	kanlowctg41234_at	Transcript	very weakly similar to (100) LOXC2_ORYSA Probable lipoxygenase 8, chloroplast precursor (EC 1.13.11.12) - Oryza sativa (Rice)very weakly similar to (100) loc_os0	2.234458
17.8.1	hormone metabolism.salicylic acid.synthesis-degradation	kanlowctg26347_at	Transcript	very weakly similar to (89.7) AT3G11480 | Symbols: BSMT1, ATBSMT1 | BSMT1; S-adenosylmethionine-dependent methyltransferase | chr3:3614544-3617137 FO	2.5175083

Cell

BinCode	BinName	id	type	description	Mo/60 (log2)
31.1	cell.organisation	kanlowctg17315_s_at	Transcript	very weakly similar to (91.7) AT1G17580 | Symbols: MYA1, ATMYA1, XI-1 | MYA1 (MYOSIN 1); motor/ protein binding | chr1:6039453-6049309 FORWARDweakly s	-1.4481871
31.1	cell.organisation	ap13itg43667_s_at	Transcript	moderately similar to (204) loc_os09g27990 12009.m05950 protein fiber annexin, putative, expressed no original description	-1.2644771
31.1	cell.organisation	alamoctg04112_s_at	Transcript	very weakly similar to (87.0) loc_os02g56840 12002.m10706 protein phloem-specific lectin, putative, expressed no original description	-1.3655809
31.1	cell.organisation	ap13ctg07009-3_s_at	Transcript	weakly similar to (129) AT5G59370 | Symbols: ACT4 | ACT4 (ACTIN 4); structural constituent of cytoskeleton | chr5:23950109-23951586 FORWARDweakly similar	-1.1169817
31.1	cell.organisation	ap13itg58453_s_at	Transcript	very weakly similar to (97.4) AT1G42550 | Symbols: PMI1 | PMI1 (PLASTID MOVEMENT IMPAIRED1) | chr1:15977131-15979734 FORWARDweakly similar to (173)	1.1272439
31.1	cell.organisation	othswctg18861_at	Transcript	very weakly similar to (83.2) AT1G01750 | Symbols: ADF11 | ADF11 (ACTIN DEPOLYMERIZING FACTOR 11); actin binding | chr1:275528-276126 FORWARDvery wea	-1.0301489
31.1	cell.organisation	ap13ctg09579_s_at	Transcript	very weakly similar to (83.2) loc_os02g56840 12002.m10706 protein phloem-specific lectin, putative, expressed no original description	-1.3673071
31.1	cell.organisation	kanlowctg39465_s_at	Transcript	weakly similar to (116) AT5G20490 | Symbols: XIK, ATXIK, XI-17 | XIK; motor/ protein binding | chr5:6927064-6936079 REVERSEweakly similar to (173) loc_os03g	-1.4964712
31.3	cell.cycle	ap13ctg04056_s_at	Transcript	very weakly similar to (92.4) loc_os05g33040 12005.m083714 protein PREG-like protein, putative, expressed no original description	1.1040965
31.3	cell.cycle	ap13ctg01864_s_at	Transcript	very weakly similar to (93.6) loc_os05g33040 12005.m083714 protein PREG-like protein, putative, expressed no original description	1.1424375
31.3.1	cell.cycle.peptidylprolyl isomerase	ap13ctg59022_at	Transcript	weakly similar to (153) AT4G38740 | Symbols: ROC1 | ROC1 (ROTAMASE CYP 1); peptidyl-prolyl cis-trans isomerase | chr4:18083620-18084138 REVERSEweakly si	-1.8485379
31.4	cell.vesicle transport	ap13ctg01136_s_at	Transcript	very weakly similar to (100) AT5G50380 | Symbols: ATEXO70F1 | ATEXO70F1 (exocyst subunit EXO70 family protein F1); protein binding | chr5:20516382-205184	1.2529333

Transport

BinCode	BinName	id	type	description	Mo/60 (log2)
34.2	transport.sugars	ap13ctg00637_at	Transcript	very weakly similar to (83.2) HEX6_RICCO Hexose carrier protein HEX6 - Ricinus communis (Castor bean)weakly similar to (112) loc_os10g41190 12010.m21970 pr	2.4313943
34.2	transport.sugars	ap13itg70211_at	Transcript	weakly similar to (158) AT3G20660 | Symbols: AtOCT4 | AtOCT4 (Arabidopsis thaliana ORGANIC CATION/CARNITINE TRANSPORTER4); carbohydrate transmembra	1.0388335
34.2	transport.sugars	ap13itg70443_s_at	Transcript	weakly similar to (143) AT4G02050 | Symbols: | sugar transporter, putative | chr4:898387-900095 REVERSEweakly similar to (148) STA_RICCO Sugar carrier prote	-1.158267
34.2	transport.sugars	ap13ctg11906_s_at	Transcript	very weakly similar to (87.8) loc_os03g10510 12003.m35080 protein outer mitochondrial membrane protein porin, putative, expressed no original description	1.4663844
34.2	transport.sugars	ap13ctg20106_s_at	Transcript	weakly similar to (149) AT4G00370 | Symbols: ANTR2, PHT4;4 | ANTR2; inorganic phosphate transmembrane transporter/ organic anion transmembrane transpor	-1.4338477
34.3	transport.amino acids	kanlowctg08378_s_at	Transcript	weakly similar to (154) AT1G77380 | Symbols: AAP3, ATAAP3 | AAP3; amino acid transmembrane transporter | chr1:29075201-29077252 REVERSEweakly similar	-1.2104665
34.3	transport.amino acids	ap13ctg18715_at	Transcript	weakly similar to (113) AT1G77380 | Symbols: AAP3, ATAAP3 | AAP3; amino acid transmembrane transporter | chr1:29075201-29077252 REVERSEweakly similar	-1.1444991
34.3	transport.amino acids	ap13ctg10274_at	Transcript	weakly similar to (103) AT1G48640 | Symbols: | lysine and histidine specific transporter, putative | chr1:17986358-17988991 FORWARDweakly similar to (118) lo	1.6404837
34.6	transport.sulphate	ap13itg48925_at	Transcript	very weakly similar to (80.5) AT1G78000 | Symbols: SULTR1;2, SEL1 | SULTR1;2 (SULFATE TRANSPORTER 1;2); sulfate transmembrane transporter | chr1:29329889	-1.2702786
34.6	transport.sulphate	kanlowctg39328_s_at	Transcript	weakly similar to (123) loc_os03g09940 12003.m06448 protein low affinity sulphate transporter 3, putative, expressed no original description	-1.0337564
34.12	transport.metal	ap13ctg59129_at	Transcript	weakly similar to (105) loc_os01g61070 12001.m12234 protein heavy metal-associated domain containing protein, expressed no original description	-1.109565
34.12	transport.metal	ap13ctg02699rc_at	Transcript	very weakly similar to (98.2) loc_os05g10940 12005.m05564 protein zinc transporter 4, chloroplast precursor, putative, expressed no original description	-1.1010977
34.13	transport.peptides and oligopeptides	ap13ctg10045_s_at	Transcript	very weakly similar to (100) AT5G64410 | Symbols: ATOPT4, OPT4 | OPT4 (OLIGOPEPTIDE TRANSPORTER 4); oligopeptide transporter | chr5:25750921-25754974	1.6726531
34.13	transport.peptides and oligopeptides	kanlowctg20886_s_at	Transcript	weakly similar to (134) AT1G65730 | Symbols: YSL7 | YSL7 (YELLOW STRIPE LIKE 7); oligopeptide transporter | chr1:24442639-24446122 FORWARDweakly similar	1.3935986
34.14	transport.unspecified cations	ap13itg65313_s_at	Transcript	very weakly similar to (99.0) AT3G19640 | Symbols: | magnesium transporter CorA-like family protein (MRS2-3) | chr3:6820969-6823104 FORWARDweakly similar	1.0777143
34.15	transport.potassium	othswslt24805_at	Transcript	weakly similar to (122) AT4G13420 | Symbols: HAK5, ATHAK5 | HAK5 (HIGH AFFINITY K+ TRANSPORTER 5); potassium ion transmembrane transporter/ potassium	1.1228445
34.16	transport.ABC transporters and multidrug resistance systems	othswslt36401_s_at	Transcript	very weakly similar to (95.5) AT3G60160 | Symbols: ATMRP9 | ATMRP9; ATPase, coupled to transmembrane movement of substances | chr3:22223829-22229195	1.5637729
34.16	transport.ABC transporters and multidrug resistance systems	othswctg14418_at	Transcript	weakly similar to (113) AT1G15520 | Symbols: PDR12, ATPDR12 | PDR12 (PLEIOTROPIC DRUG RESISTANCE 12); ATPase, coupled to transmembrane movement of s	1.2239101
34.16	transport.ABC transporters and multidrug resistance systems	kanlowctg23219_s_at	Transcript	weakly similar to (129) AT3G60970 | Symbols: ATMRP15 | ATMRP15; ATPase, coupled to transmembrane movement of substances | chr3:22557535-22561575 FC	1.429423
34.16	transport.ABC transporters and multidrug resistance systems	kanlowctg38102_s_at	Transcript	weakly similar to (124) AT3G60970 | Symbols: ATMRP15 | ATMRP15; ATPase, coupled to transmembrane movement of substances | chr3:22557535-22561575 FC	1.4499054
34.16	transport.ABC transporters and multidrug resistance systems	kanlowctg47473rc_s_a	Transcript	weakly similar to (120) AT3G60160 | Symbols: ATMRP9 | ATMRP9; ATPase, coupled to transmembrane movement of substances | chr3:22223829-22229195 REVE	1.1958963
34.16	transport.ABC transporters and multidrug resistance systems	othswctg17884rc_s_at	Transcript	weakly similar to (132) AT1G02520 | Symbols: PGP11 | PGP11 (P-GLYCOPROTEIN 11); ATPase, coupled to transmembrane movement of substances | chr1:524134	1.0449052
34.16	transport.ABC transporters and multidrug resistance systems	othswctg12305_s_at	Transcript	very weakly similar to (95.9) AT3G43790 | Symbols: ZIFL2 | ZIFL2 (ZINC INDUCED FACILITATOR-like 2); carbohydrate transmembrane transporter/ sugar:hydrogen s	2.0609794
34.16	transport.ABC transporters and multidrug resistance systems	ap13ctg11369-1_s_at	Transcript	very weakly similar to (84.0) AT3G60970 | Symbols: ATMRP15 | ATMRP15; ATPase, coupled to transmembrane movement of substances | chr3:22557535-225615	1.2177197
34.16	transport.ABC transporters and multidrug resistance systems	ap13ctg15102_s_at	Transcript	weakly similar to (133) AT1G66950 | Symbols: PDR11, ATPDR11 | PDR11 (PLEIOTROPIC DRUG RESISTANCE 11); ATPase, coupled to transmembrane movement of s	3.3915749
34.99	transport.misc	othswctg19026_s_at	Transcript	weakly similar to (135) AT1G20925 | Symbols: | auxin efflux carrier family protein | chr1:7290612-7292507 FORWARDweakly similar to (165) loc_os09g31478 12(	1.2368882
34.99	transport.misc	kanlowctg12598_s_at	Transcript	weakly similar to (133) AT1G01630 | Symbols: | SEC14 cytosolic factor, putative / phosphoglyceride transfer protein, putative | chr1:229206-230675 FORWARDwe	2.9746048

List of significantly DEGs involved in hormone, transport, misc and major CHO pathway in Se/60 comparison

Hormone

BinCode	BinName	id	type	description	Se/60 (log2)
17.1.1	hormone metabolism.abscisic acid.synthesis-degradation	ap13itg64998_at	Transcript	very weakly similar to (95.1) loc_os04g46470 12004.m09596 protein dioxygenase RAMOSUS5, putative, expressed no original description	1.784
17.2.3	hormone metabolism.auxin.induced-regulated-responsive-activated	ap13itg71573-rc_at	Transcript	no original description	2.353
17.2.3	hormone metabolism.auxin.induced-regulated-responsive-activated	ap13itg62309_at	Transcript	weakly similar to (172) AT1G60730 | Symbols: | aldo/keto reductase family protein | chr1:22358613-22360082 REVERSEweakly similar to (115) A115_TOBAC Aux	2.472
17.2.3	hormone metabolism.auxin.induced-regulated-responsive-activated	ap13itg58736_s_at	Transcript	very weakly similar to (85.5) loc_os09g37430 12009.m06690 protein OsSAUR48 - Auxin-responsive SAUR gene family member no original description	-1.785
17.2.3	hormone metabolism.auxin.induced-regulated-responsive-activated	ap13itg44930_s_at	Transcript	very weakly similar to (93.6) GH312_ORYSA Probable indole-3-acetic acid-amido synthetase GH3.12 (EC 6.3.2.-) (Auxin-responsive GH3-like protein 12) (OsGH3-12)	-1.134
17.2.3	hormone metabolism.auxin.induced-regulated-responsive-activated	kanlowctg33522_at	Transcript	weakly similar to (138) AT1G60730 | Symbols: | aldo/keto reductase family protein | chr1:22358613-22360082 REVERSEweakly similar to (106) A115_TOBAC Aux	2.143
17.2.3	hormone metabolism.auxin.induced-regulated-responsive-activated	ap13itg70591-rc_at	Transcript	weakly similar to (111) loc_os04g51890 12004.m10084 protein OsSAUR20 - Auxin-responsive SAUR gene family member, expressed no original description	3.184
17.2.3	hormone metabolism.auxin.induced-regulated-responsive-activated	ap13ctg17086_at	Transcript	weakly similar to (103) IAA6_ORYSA Auxin-responsive protein IAA6 (Indoleacetic acid-induced protein 6) - Oryza sativa (Rice)weakly similar to (103) loc_os01g538	2.052

<table>
<tr><td>17.3.1.2.8</td><td>hormone metabolism.brassinosteroid.synthesis-degradation.sterols.D\</td><td>ap13ctg15125_s_at</td><td>Transcript</td><td>weakly similar to (195) AT3G19820 | Symbols: DWF1, DIM, EVE1, DIM1, CBB1 | DWF1 (DWARF 1); calmodulin binding / catalytic | chr3:6879835-6881616 REVERS</td><td>1.596</td></tr>
<tr><td>17.5.1</td><td>hormone metabolism.ethylene.synthesis-degradation</td><td>ap13itg74891-rc_at</td><td>Transcript</td><td>weakly similar to (196) AT1G06620 | Symbols: | 2-oxoglutarate-dependent dioxygenase, putative | chr1:2025618-2027094 FORWARDweakly similar to (187) DV4</td><td>1.023</td></tr>
<tr><td>17.6.1.1</td><td>hormone metabolism.gibberelin.synthesis-degradation.copalyl diphos</td><td>othswctg09951_s_at</td><td>Transcript</td><td>very weakly similar to (87.8) AT4G02780 | Symbols: GA1, ABC33, ATCPS1 | GA1 (GA REQUIRING 1); ent-copalyl diphosphate synthase/ magnesium ion binding | ch</td><td>2.333</td></tr>
<tr><td>17.6.1.1</td><td>hormone metabolism.gibberelin.synthesis-degradation.copalyl diphos</td><td>othswctg07572_at</td><td>Transcript</td><td>weakly similar to (105) loc_os02g17780 12002.m07022 protein ent-kaurene synthase A, chloroplast precursor, putative, expressed no original description</td><td>2.139</td></tr>
<tr><td>17.6.1.1</td><td>hormone metabolism.gibberelin.synthesis-degradation.copalyl diphos</td><td>ap13itg72235_s_at</td><td>Transcript</td><td>weakly similar to (197) loc_os04g09900 12004.m35118 protein ent-kaurene synthase A, chloroplast precursor, putative, expressed no original description</td><td>2.274</td></tr>
<tr><td>17.6.1.3</td><td>hormone metabolism.gibberelin.synthesis-degradation.ent-kaurene ox</td><td>kanlowctg40278_s_at</td><td>Transcript</td><td>weakly similar to (136) AT5G25900 | Symbols: GA3, CYP701A3, ATKO1 | GA3 (GA REQUIRING 3); ent-kaurene oxidase/ oxygen binding | chr5:9036073-9038278 F</td><td>2.266</td></tr>
<tr><td>17.7.1.2</td><td>hormone metabolism.jasmonate.synthesis-degradation.lipoxygenase</td><td>ap13itg70317_at</td><td>Transcript</td><td>weakly similar to (143) AT3G45140 | Symbols: LOX2, ATLOX2 | LOX2 (LIPOXYGENASE 2); lipoxygenase | chr3:16525437-16529233 FORWARDweakly similar to (1</td><td>2.746</td></tr>
<tr><td>17.7.1.5</td><td>hormone metabolism.jasmonate.synthesis-degradation.12-Oxo-PDA-re</td><td>kanlowctg23007_at</td><td>Transcript</td><td>weakly similar to (176) AT1G76690 | Symbols: OPR2, ATOPR2 | OPR2 | chr1:28778976-28780355 FORWARDmoderately similar to (241) loc_os06g11210 12006.m</td><td>5.946</td></tr>
<tr><td>17.7.1.5</td><td>hormone metabolism.jasmonate.synthesis-degradation.12-Oxo-PDA-re</td><td>ap13itg71073_s_at</td><td>Transcript</td><td>weakly similar to (110) AT1G76690 | Symbols: OPR2, ATOPR2 | OPR2 | chr1:28778976-28780355 FORWARDweakly similar to (167) loc_os06g11210 12006.m058</td><td>4.576</td></tr>
<tr><td>17.7.1.5</td><td>hormone metabolism.jasmonate.synthesis-degradation.12-Oxo-PDA-re</td><td>ap13itg67010_s_at</td><td>Transcript</td><td>weakly similar to (115) AT1G76680 | Symbols: OPR1, ATOPR1 | OPR1; 12-oxophytodienoate reductase | chr1:28776982-28778271 FORWARDweakly similar to (1</td><td>3.127</td></tr>
<tr><td>17.7.1.5</td><td>hormone metabolism.jasmonate.synthesis-degradation.12-Oxo-PDA-re</td><td>ap13itg69033_at</td><td>Transcript</td><td>weakly similar to (162) AT1G76690 | Symbols: OPR2, ATOPR2 | OPR2 | chr1:28778976-28780355 FORWARDmoderately similar to (220) loc_os06g11290 12006.m</td><td>2.318</td></tr>
<tr><td>17.8.1</td><td>hormone metabolism.salicylic acid.synthesis-degradation</td><td>kanlowctg26347_at</td><td>Transcript</td><td>very weakly similar to (89.7) AT3G11480 | Symbols: BSMT1, ATBSMT1 | BSMT1; S-adenosylmethionine-dependent methyltransferase | chr3:3614544-3617137 FO</td><td>2.935</td></tr>
<tr><td>17.8.1</td><td>hormone metabolism.salicylic acid.synthesis-degradation</td><td>ap13ctg19731_s_at</td><td>Transcript</td><td>weakly similar to (119) AT3G11480 | Symbols: BSMT1, ATBSMT1 | BSMT1; S-adenosylmethionine-dependent methyltransferase | chr3:3614544-3617137 FORWA</td><td>1.674</td></tr>
</table>

Transport

<table>
<tr><th>BinCode</th><th>BinName</th><th>id</th><th>type</th><th>description</th><th>Se/60 (log2)</th></tr>
<tr><td>34.2</td><td>transport.sugars</td><td>ap13itg70443_s_at</td><td>Transcript</td><td>weakly similar to (143) AT4G02050 | Symbols: | sugar transporter, putative | chr4:898387-900095 REVERSEweakly similar to (148) STA_RICCO Sugar carrier prote</td><td>-1.4809461</td></tr>
<tr><td>34.3</td><td>transport.amino acids</td><td>ap13ctg25184_s_at</td><td>Transcript</td><td>weakly similar to (134) loc_os04g38680 12004.m08857 protein amino acid/polyamine transporter II, putative, expressed no original description</td><td>2.2175243</td></tr>
<tr><td>34.6</td><td>transport.sulphate</td><td>ap13ctg19867_at</td><td>Transcript</td><td>weakly similar to (107) AT1G22150 | Symbols: SULTR1;3 | SULTR1;3; sulfate transmembrane transporter | chr1:7818361-7821344 FORWARDweakly similar to (11</td><td>1.7543813</td></tr>
<tr><td>34.6</td><td>transport.sulphate</td><td>kanlowctg46743_s_at</td><td>Transcript</td><td>very weakly similar to (96.3) loc_os01g52130 12001.m11387 protein sulfate transporter 3.5, putative, expressed no original description</td><td>2.3649237</td></tr>
<tr><td>34.12</td><td>transport.metal</td><td>ap13ctg25265_s_at</td><td>Transcript</td><td>very weakly similar to (95.5) AT1G63440 | Symbols: HMA5 | HMA5 (HEAVY METAL ATPASE 5); ATPase, coupled to transmembrane movement of ions, phosphoryla</td><td>-1.46212</td></tr>
<tr><td>34.12</td><td>transport.metal</td><td>ap13itg53636_s_at</td><td>Transcript</td><td>very weakly similar to (100) AT1G80830 | Symbols: NRAMP1, PMIT1, ATNRAMP1 | NRAMP1 (NATURAL RESISTANCE-ASSOCIATED MACROPHAGE PROTEIN 1); inor</td><td>-1.0665691</td></tr>
<tr><td>34.13</td><td>transport.peptides and oligopeptides</td><td>ap13ctg13702_s_at</td><td>Transcript</td><td>very weakly similar to (82.4) AT4G10770 | Symbols: ATOPT7, OPT7 | OPT7 (OLIGOPEPTIDE TRANSPORTER 7); oligopeptide transporter | chr4:6628646-6632517 RE</td><td>2.3295274</td></tr>
<tr><td>34.15</td><td>transport.potassium</td><td>kanlowctg10065_at</td><td>Transcript</td><td>very weakly similar to (98.2) loc_os01g70490 12001.m13096 protein potassium transporter 5, putative, expressed no original description</td><td>3.421157</td></tr>
<tr><td>34.16</td><td>transport.ABC transporters and multidrug resistance systems</td><td>ap13ctg24230_at</td><td>Transcript</td><td>very weakly similar to (93.6) AT3G62150 | Symbols: PGP21 | PGP21 (P-GLYCOPROTEIN 21); ATPase, coupled to transmembrane movement of substances | chr3:23</td><td>3.3023396</td></tr>
<tr><td>34.16</td><td>transport.ABC transporters and multidrug resistance systems</td><td>othswslt36401_s_at</td><td>Transcript</td><td>very weakly similar to (95.5) AT3G60160 | Symbols: ATMRP9 | ATMRP9; ATPase, coupled to transmembrane movement of substances | chr3:22223829-22229195</td><td>2.6775858</td></tr>
<tr><td>34.16</td><td>transport.ABC transporters and multidrug resistance systems</td><td>ap13ctg27505_s_at</td><td>Transcript</td><td>very weakly similar to (81.3) AT5G52050 | Symbols: | MATE efflux protein-related | chr5:21138933-21140450 FORWARDweakly similar to (119) loc_os04g48290 1</td><td>-1.0269383</td></tr>
<tr><td>34.16</td><td>transport.ABC transporters and multidrug resistance systems</td><td>othswctg05668_s_at</td><td>Transcript</td><td>very weakly similar to (81.3) loc_os12g22284 12012.m06072 protein ATP-binding cassette sub-family G member 2, putative, expressed no original description</td><td>-1.1764</td></tr>
<tr><td>34.16</td><td>transport.ABC transporters and multidrug resistance systems</td><td>ap13itg73678_at</td><td>Transcript</td><td>weakly similar to (125) loc_os08g43120 12008.m08288 protein ABC transporter, putative, expressed no original description</td><td>-1.4856094</td></tr>
<tr><td>34.16</td><td>transport.ABC transporters and multidrug resistance systems</td><td>kanlowctg45231_s_at</td><td>Transcript</td><td>weakly similar to (138) AT5G61700 | Symbols: ATATH16, ATH16 | ATATH16; ATPase, coupled to transmembrane movement of substances / transporter | chr5:24</td><td>2.4373958</td></tr>
<tr><td>34.16</td><td>transport.ABC transporters and multidrug resistance systems</td><td>kanlowctg20944_at</td><td>Transcript</td><td>moderately similar to (271) AT4G18050 | Symbols: PGP9 | PGP9 (P-GLYCOPROTEIN 9); ATPase, coupled to transmembrane movement of substances | chr4:10022</td><td>3.7330322</td></tr>
<tr><td>34.16</td><td>transport.ABC transporters and multidrug resistance systems</td><td>ap13ctg07825_at</td><td>Transcript</td><td>weakly similar to (146) AT2G28070 | Symbols: | ABC transporter family protein | chr2:11956432-11959782 FORWARDweakly similar to (188) loc_os03g64200 12</td><td>1.0885957</td></tr>
<tr><td>34.16</td><td>transport.ABC transporters and multidrug resistance systems</td><td>ap13ctg30924_x_at</td><td>Transcript</td><td>weakly similar to (106) PDR2_ORYSA Probable pleiotropic drug resistance protein 2 - Oryza sativa (Rice)weakly similar to (106) loc_os01g42370 12001.m10503 pr</td><td>1.8209134</td></tr>
<tr><td>34.16</td><td>transport.ABC transporters and multidrug resistance systems</td><td>ap13ctg24230_s_at</td><td>Transcript</td><td>moderately similar to (213) AT4G18050 | Symbols: PGP9 | PGP9 (P-GLYCOPROTEIN 9); ATPase, coupled to transmembrane movement of substances | chr4:10022</td><td>3.2684987</td></tr>
<tr><td>34.16</td><td>transport.ABC transporters and multidrug resistance systems</td><td>kanlowctg23219_s_at</td><td>Transcript</td><td>weakly similar to (129) AT3G60970 | Symbols: ATMRP15 | ATMRP15; ATPase, coupled to transmembrane movement of substances | chr3:22557535-22561575 F</td><td>2.278649</td></tr>
<tr><td>34.16</td><td>transport.ABC transporters and multidrug resistance systems</td><td>kanlowctg38102_s_at</td><td>Transcript</td><td>weakly similar to (124) AT3G60970 | Symbols: ATMRP15 | ATMRP15; ATPase, coupled to transmembrane movement of substances | chr3:22557535-22561575 F</td><td>2.5144386</td></tr>
<tr><td>34.16</td><td>transport.ABC transporters and multidrug resistance systems</td><td>ap13itg73678rc_s_at</td><td>Transcript</td><td>very weakly similar to (95.5) loc_os08g43120 12008.m08288 protein ABC transporter, putative, expressed no original description</td><td>-1.2439208</td></tr>
<tr><td>34.16</td><td>transport.ABC transporters and multidrug resistance systems</td><td>kanlowctg47473rc_s_a</td><td>Transcript</td><td>weakly similar to (120) AT3G60160 | Symbols: ATMRP9 | ATMRP9; ATPase, coupled to transmembrane movement of substances | chr3:22223829-22229195 REV</td><td>1.8580594</td></tr>
<tr><td>34.16</td><td>transport.ABC transporters and multidrug resistance systems</td><td>ap13ctg11369-1_s_at</td><td>Transcript</td><td>very weakly similar to (84.0) AT3G60970 | Symbols: ATMRP15 | ATMRP15; ATPase, coupled to transmembrane movement of substances | chr3:22557535-225615</td><td>1.9506253</td></tr>
<tr><td>34.16</td><td>transport.ABC transporters and multidrug resistance systems</td><td>ap13ctg12939-2_at</td><td>Transcript</td><td>very weakly similar to (87.8) loc_os01g52550 12001.m11428 protein multidrug resistance protein 22, putative, expressed no original description</td><td>1.6844735</td></tr>
<tr><td>34.16</td><td>transport.ABC transporters and multidrug resistance systems</td><td>kanlowctg38775_s_at</td><td>Transcript</td><td>weakly similar to (155) AT3G47760 | Symbols: ATATH4, ATH4 | ATATH4; ATPase, coupled to transmembrane movement of substances / transporter | chr3:17611</td><td>1.8288375</td></tr>
<tr><td>34.16</td><td>transport.ABC transporters and multidrug resistance systems</td><td>ap13itg61011_at</td><td>Transcript</td><td>weakly similar to (156) AT4G01830 | Symbols: PGP5 | PGP5 (P-GLYCOPROTEIN 5); ATPase, coupled to transmembrane movement of substances | chr4:785683-79</td><td>1.834321</td></tr>
<tr><td>34.16</td><td>transport.ABC transporters and multidrug resistance systems</td><td>kanlowslt56395_at</td><td>Transcript</td><td>weakly similar to (109) AT4G18050 | Symbols: PGP9 | PGP9 (P-GLYCOPROTEIN 9); ATPase, coupled to transmembrane movement of substances | chr4:10022205-</td><td>3.5532231</td></tr>
<tr><td>34.99</td><td>transport.misc</td><td>kanlowctg46528_s_at</td><td>Transcript</td><td>weakly similar to (114) AT5G52450 | Symbols: | MATE efflux protein-related | chr5:21289042-21291749 REVERSEweakly similar to (156) loc_os10g11354 12010.</td><td>2.378194</td></tr>
<tr><td>34.99</td><td>transport.misc</td><td>ap13ctg27505_s_at</td><td>Transcript</td><td>very weakly similar to (81.3) AT5G52050 | Symbols: | MATE efflux protein-related | chr5:21138933-21140450 FORWARDweakly similar to (119) loc_os04g48290 1</td><td>-1.0269383</td></tr>
<tr><td>34.99</td><td>transport.misc</td><td>othswctg19026_s_at</td><td>Transcript</td><td>weakly similar to (135) AT1G20925 | Symbols: | auxin efflux carrier family protein | chr1:7290612-7292507 FORWARDweakly similar to (165) loc_os09g31478 12</td><td>2.0320575</td></tr>
<tr><td>34.99</td><td>transport.misc</td><td>ap13itg52747_s_at</td><td>Transcript</td><td>very weakly similar to (96.3) AT5G65980 | Symbols: | auxin efflux carrier family protein | chr5:26392444-26394232 FORWARDweakly similar to (120) loc_os09g38</td><td>1.7859634</td></tr>
<tr><td>34.99</td><td>transport.misc</td><td>kanlowctg12598_s_at</td><td>Transcript</td><td>weakly similar to (133) AT1G01630 | Symbols: | SEC14 cytosolic factor, putative / phosphoglyceride transfer protein, putative | chr1:229206-230675 FORWARDw</td><td>3.1054966</td></tr>
</table>

Misc

<table>
<tr><th>BinCode</th><th>BinName</th><th>id</th><th>type</th><th>description</th><th>Se/60 (log2)</th></tr>
<tr><td>26.1</td><td>misc.misc2</td><td>ap13ctg15239_at</td><td>Transcript</td><td>weakly similar to (116) loc_os10g35500 12010.m06374 protein epoxide hydrolase, putative, expressed no original description</td><td>1.9422607</td></tr>
<tr><td>26.1</td><td>misc.misc2</td><td>ap13itg58554_at</td><td>Transcript</td><td>very weakly similar to (85.9) loc_os05g33110 12005.m083715 protein endo-1,3;1,4-beta-D-glucanase precursor, putative, expressed no original description</td><td>3.5439093</td></tr>
<tr><td>26.1</td><td>misc.misc2</td><td>ap13ctg11976_s_at</td><td>Transcript</td><td>very weakly similar to (87.8) C71C2_MAIZE Cytochrome P450 71C2 (EC 1.14.-.-) (Benzoxazineless 3) - Zea mays (Maize)weakly similar to (104) loc_os06g22020 120</td><td>1.6006612</td></tr>
<tr><td>26.1</td><td>misc.misc2</td><td>ap13itg73544_at</td><td>Transcript</td><td>weakly similar to (140) loc_os10g35500 12010.m06374 protein epoxide hydrolase, putative, expressed no original description</td><td>2.6115177</td></tr>
<tr><td>26.1</td><td>misc.misc2</td><td>ap13itg62754_s_at</td><td>Transcript</td><td>very weakly similar to (81.3) AT3G23600 | Symbols: | dienelactone hydrolase family protein | chr3:8473833-8475655 FORWARDweakly similar to (105) loc_os05g</td><td>2.6760173</td></tr>
<tr><td>26.1</td><td>misc.misc2</td><td>ap13ctg56716_at</td><td>Transcript</td><td>weakly similar to (144) loc_os10g35500 12010.m06374 protein epoxide hydrolase, putative, expressed no original description</td><td>1.0979897</td></tr>
<tr><td>26.1</td><td>misc.misc2</td><td>ap13ctg12203_at</td><td>Transcript</td><td>weakly similar to (112) AT4G19185 | Symbols: | integral membrane family protein | chr4:10489201-10491488 REVERSEweakly similar to (157) loc_os06g49500 1</td><td>2.7201333</td></tr>
<tr><td>26.2</td><td>misc.UDP glucosyl and glucoronyl transferases</td><td>ap13itg60355_at</td><td>Transcript</td><td>weakly similar to (151) AT1G22370 | Symbols: AtUGT85A5 | AtUGT85A5 (UDP-glucosyl transferase 85A5); glucuronosyltransferase/ transferase, transferring glyco</td><td>-1.5335133</td></tr>
<tr><td>26.2</td><td>misc.UDP glucosyl and glucoronyl transferases</td><td>ap13itg65392_at</td><td>Transcript</td><td>very weakly similar to (100) loc_os05g08750 12005.m05389 protein cold-induced glucosyl transferase, putative, expressed no original description</td><td>1.521799</td></tr>
<tr><td>26.2</td><td>misc.UDP glucosyl and glucoronyl transferases</td><td>ap13itg67351_at</td><td>Transcript</td><td>very weakly similar to (93.2) CZOG2_MAIZE Cis-zeatin O-glucosyltransferase 2 (EC 2.4.1.215) (cisZOG2) - Zea mays (Maize)very weakly similar to (87.8) loc_os04g46</td><td>1.0609822</td></tr>
<tr><td>26.2</td><td>misc.UDP glucosyl and glucoronyl transferases</td><td>kanlowctg46878_s_at</td><td>Transcript</td><td>weakly similar to (145) AT4G02500 | Symbols: ATXT2, XXT2, XT2 | XT2 (UDP-XYLOSYLTRANSFERASE 2); UDP-xylosyltransferase/ transferase/ transferase, transferri</td><td>-1.075472</td></tr>
<tr><td>26.2</td><td>misc.UDP glucosyl and glucoronyl transferases</td><td>kanlowctg42344_s_at</td><td>Transcript</td><td>very weakly similar to (92.8) AT2G38650 | Symbols: GAUT7, LGT7 | GAUT7 (GALACTURONOSYLTRANSFERASE 7); polygalacturonate 4-alpha-galacturonosyltransfer</td><td>-1.0060872</td></tr>
<tr><td>26.2</td><td>misc.UDP glucosyl and glucoronyl transferases</td><td>ap13itg67285_at</td><td>Transcript</td><td>weakly similar to (146) loc_os10g07970 12010.m04140 protein anthocyanidin 5,3-O-glucosyltransferase, putative, expressed no original description</td><td>1.2526202</td></tr>
<tr><td>26.2</td><td>misc.UDP glucosyl and glucoronyl transferases</td><td>ap13itg62195_at</td><td>Transcript</td><td>weakly similar to (117) AT3G02100 | Symbols: | UDP-glucoronosyl/UDP-glucosyl transferase family protein | chr3:368840-370484 REVERSEvery weakly similar to (</td><td>1.2497023</td></tr>
<tr><td>26.2</td><td>misc.UDP glucosyl and glucoronyl transferases</td><td>ap13itg68735_at</td><td>Transcript</td><td>weakly similar to (107) AT4G01070 | Symbols: GT72B1, UGT72B1 | GT72B1; UDP-glucosyltransferase/ UDP-glycosyltransferase/ transferase, transferring glycosyl</td><td>1.0957323</td></tr>
<tr><td>26.2</td><td>misc.UDP glucosyl and glucoronyl transferases</td><td>kanlowctg41220_s_at</td><td>Transcript</td><td>very weakly similar to (95.5) loc_os06g13760 12006.m06091 protein transferase, transferring glycosyl groups, putative, expressed no original description</td><td>-1.2659137</td></tr>
<tr><td>26.2</td><td>misc.UDP glucosyl and glucoronyl transferases</td><td>kanlowctg23500_s_at</td><td>Transcript</td><td>weakly similar to (125) AT1G07240 | Symbols: UGT71C5 | UGT71C5 (UDP-GLUCOSYL TRANSFERASE 71C5); UDP-glycosyltransferase/ quercetin 3-O-glucosyltransfe</td><td>1.5789853</td></tr>
<tr><td>26.2</td><td>misc.UDP glucosyl and glucoronyl transferases</td><td>ap13itg75785rc_at</td><td>Transcript</td><td>moderately similar to (202) AT1G05680 | Symbols: | UDP-glucoronosyl/UDP-glucosyl transferase family protein | chr1:1703196-1704639 REVERSEweakly similar t</td><td>2.163164</td></tr>
<tr><td>26.2</td><td>misc.UDP glucosyl and glucoronyl transferases</td><td>ap13itg74837_at</td><td>Transcript</td><td>very weakly similar to (95.1) AT1G01390 | Symbols: | UDP-glucoronosyl/UDP-glucosyl transferase family protein | chr1:148319-149761 REVERSEweakly similar to (</td><td>3.7462993</td></tr>
<tr><td>26.2</td><td>misc.UDP glucosyl and glucoronyl transferases</td><td>kanlowctg14906_s_at</td><td>Transcript</td><td>weakly similar to (119) loc_os01g45110 12001.m10717 protein cytokinin-O-glucosyltransferase 1, putative, expressed no original description</td><td>2.3178332</td></tr>
</table>

26.2	misc.UDP glucosyl and glucoronyl transferases	ap13itg47952_s_at	Transcript	weakly similar to (117) loc_os03g55050 12003.m10421 protein cytokinin-O-glucosyltransferase 2, putative, expressed no original description	2.4126816
26.2	misc.UDP glucosyl and glucoronyl transferases	ap13ctg09529_at	Transcript	weakly similar to (162) AT1G05680 | Symbols: | UDP-glucoronosyl/UDP-glucosyl transferase family protein | chr1:1703196-1704639 REVERSEweakly similar to (1	3.837729
26.3	misc.gluco-, galacto- and mannosidases	kanlowctg22294_at	Transcript	weakly similar to (137) loc_os01g16310 12001.m08227 protein glycosyl hydrolases family 31 protein, expressed no original description	3.1825423
26.3	misc.gluco-, galacto- and mannosidases	alamoctg05610_at	Transcript	very weakly similar to (89.7) loc_os10g05069 12010.m22012 protein lysosomal alpha-mannosidase precursor, putative, expressed no original description	-2.55163
26.3	misc.gluco-, galacto- and mannosidases	othswctg11061_at	Transcript	weakly similar to (150) AT1G61820 | Symbols: BGLU46 | BGLU46 (BETA GLUCOSIDASE 46); catalytic/ cation binding / hydrolase, hydrolyzing O-glycosyl compound	-1.4892396
26.3	misc.gluco-, galacto- and mannosidases	ap13ctg34009_s_at	Transcript	weakly similar to (111) AT4G27820 | Symbols: BGLU9 | BGLU9 (BETA GLUCOSIDASE 9); catalytic/ cation binding / hydrolase, hydrolyzing O-glycosyl compounds | c	3.4901724
26.3	misc.gluco-, galacto- and mannosidases	kanlowctg08905_s_at	Transcript	weakly similar to (108) loc_os05g30350 12005.m27797 protein non-cyanogenic beta-glucosidase precursor, putative, expressed no original description	3.3038359
26.3	misc.gluco-, galacto- and mannosidases	kanlowctg19994_s_at	Transcript	very weakly similar to (100) AT4G21760 | Symbols: BGLU47 | BGLU47 (Beta-glucosidase 47); catalytic/ cation binding / hydrolase, hydrolyzing O-glycosyl compour	2.0472379
26.3	misc.gluco-, galacto- and mannosidases	ap13itg52053_s_at	Transcript	very weakly similar to (82.0) AT1G61810 | Symbols: BGLU45 | BGLU45 (BETA-GLUCOSIDASE 45); catalytic/ cation binding / hydrolase, hydrolyzing O-glycosyl comp	3.2846618
26.3.2	misc.gluco-, galacto- and mannosidases.beta-galactosidase	ap13ctg00450_s_at	Transcript	weakly similar to (106) AT2G32810 | Symbols: BGAL9 | BGAL9 (Beta galactosidase 9); beta-galactosidase/ catalytic/ cation binding / sugar binding | chr2:1391941	1.6042432
26.3.4	misc.gluco-, galacto- and mannosidases.endoglucanase	kanlowctg19895_x_at	Transcript	weakly similar to (152) AT5G49720 | Symbols: ATGH9A1, DEC, KOR, RSW2, IRX2, KOR1 | ATGH9A1 (ARABIDOPSIS THALIANA GLYCOSYL HYDROLASE 9A1); cellulase	1.6259547
26.4	misc.beta 1,3 glucan hydrolases	ap13itg58279_s_at	Transcript	weakly similar to (122) AT3G07320 | Symbols: | glycosyl hydrolase family 17 protein | chr3:2332324-2333925 REVERSEweakly similar to (177) loc_os09g32550 12	1.6846392
26.4.1	misc.beta 1,3 glucan hydrolases.glucan endo-1,3-beta-glucosidase	ap13itg70254_at	Transcript	weakly similar to (113) AT4G16260 | Symbols: | catalytic/ cation binding / hydrolase, hydrolyzing O-glycosyl compounds | chr4:9200180-9201441 REVERSEweakly	2.7916436
26.4.1	misc.beta 1,3 glucan hydrolases.glucan endo-1,3-beta-glucosidase	ap13itg65652_at	Transcript	weakly similar to (122) AT3G57260 | Symbols: BGL2, PR2, BG2, PR-2 | BGL2 (BETA-1,3-GLUCANASE 2); cellulase/ glucan 1,3-beta-glucosidase/ hydrolase, hydrolyzi	1.9357486
26.7	misc.oxidases - copper, flavone etc	kanlowctg47289_s_at	Transcript	weakly similar to (107) loc_os03g05900 12003.m101192 protein monooxygenase, putative, expressed no original description	3.107287
26.7	misc.oxidases - copper, flavone etc	kanlowctg44665_s_at	Transcript	weakly similar to (108) loc_os03g05900 12003.m101192 protein monooxygenase, putative, expressed no original description	2.4343748
26.7	misc.oxidases - copper, flavone etc	ap13ctg04320_at	Transcript	weakly similar to (155) loc_os04g14690 12004.m06706 protein monooxygenase/ oxidoreductase, putative, expressed no original description	1.1771873
26.7	misc.oxidases - copper, flavone etc	ap13ctg02095_at	Transcript	weakly similar to (103) loc_os09g37620 12009.m06709 protein monooxygenase/ oxidoreductase, putative, expressed no original description	2.7133992
26.8	misc.nitrilases, *nitrile lyases, berberine bridge enzymes, reticuline oxi	kanlowctg36954_at	Transcript	very weakly similar to (91.3) AT2G29330 | Symbols: TRI | TRI (tropinone reductase); binding / catalytic/ oxidoreductase | chr2:12594604-12596196 FORWARDwea	1.3482957
26.9	misc.glutathione S transferases	ap13ctg18609_at	Transcript	very weakly similar to (91.3) AT3G55040 | Symbols: GSTL2 | GSTL2 | chr3:20398718-20400305 REVERSEvery weakly similar to (97.4) IN21_MAIZE IN2-1 protein - Z	3.4394202
26.9	misc.glutathione S transferases	ap13itg59978_at	Transcript	very weakly similar to (82.0) GSTU6_ORYSA Probable glutathione S-transferase GSTU6 (EC 2.5.1.18) (28 kDa cold-induced protein) - Oryza sativa (Rice)weakly simil	1.8530868
26.9	misc.glutathione S transferases	ap13itg52395_s_at	Transcript	very weakly similar to (89.7) AT1G10370 | Symbols: GST30, ATGSTU17, GST30B, ERD9 | ERD9 (EARLY-RESPONSIVE TO DEHYDRATION 9); glutathione transferase | c	2.6520345
26.9	misc.glutathione S transferases	ap13ctg23895_at	Transcript	weakly similar to (116) GSTU6_ORYSA Probable glutathione S-transferase GSTU6 (EC 2.5.1.18) (28 kDa cold-induced protein) - Oryza sativa (Rice)weakly similar to	2.3023114
26.9	misc.glutathione S transferases	ap13itg64056_at	Transcript	weakly similar to (107) loc_os10g38340 12010.m06607 protein glutathione S-transferase GSTU6, putative, expressed no original description	2.9526143
26.9	misc.glutathione S transferases	ap13ctg24964_at	Transcript	very weakly similar to (93.6) AT3G62760 | Symbols: ATGSTF13 | ATGSTF13; glutathione transferase | chr3:23217425-23218246 REVERSEweakly similar to (135) G	1.6238625
26.9	misc.glutathione S transferases	ap13ctg16606_s_at	Transcript	very weakly similar to (90.5) AT3G55040 | Symbols: GSTL2 | GSTL2 | chr3:20398718-20400305 REVERSEweakly similar to (149) IN21_MAIZE IN2-1 protein - Zea m	1.6981143
26.9	misc.glutathione S transferases	ap13itg54282_at	Transcript	very weakly similar to (92.4) AT3G62760 | Symbols: ATGSTF13 | ATGSTF13; glutathione transferase | chr3:23217425-23218246 REVERSEweakly similar to (164) G	1.7701411
26.1	misc.cytochrome P450	ap13itg42917_at	Transcript	weakly similar to (142) AT2G45560 | Symbols: CYP76C1 | CYP76C1; electron carrier/ heme binding / iron ion binding / monooxygenase | chr2:18777240-1877835	1.3938608
26.1	misc.cytochrome P450	ap13itg17678_at	Transcript	weakly similar to (149) AT2G46950 | Symbols: CYP709B2 | CYP709B2; electron carrier/ heme binding / iron ion binding / monooxygenase/ oxygen binding | chr2:1	-1.2707534
26.1	misc.cytochrome P450	ap13itg72766_at	Transcript	weakly similar to (125) AT5G36130 | Symbols: | cytochrome P450 family | chr5:14209293-14209811 REVERSEvery weakly similar to (87.8) C90D2_ORYSA Cytochr	1.8341962
26.1	misc.cytochrome P450	ap13ctg15593_s_at	Transcript	very weakly similar to (89.7) AT3G14620 | Symbols: CYP72A8 | CYP72A8; electron carrier/ heme binding / iron ion binding / monooxygenase/ oxygen binding | chr	4.025958
26.1	misc.cytochrome P450	kanlowctg40278_s_at	Transcript	weakly similar to (136) AT5G25900 | Symbols: GA3, CYP701A3, ATKO1 | GA3 (GA REQUIRING 3); ent-kaurene oxidase/ oxygen binding | chr5:9036073-9038278 F	2.2658863
26.1	misc.cytochrome P450	kanlowctg47117_s_at	Transcript	weakly similar to (130) AT3G26300 | Symbols: CYP71B34 | CYP71B34; electron carrier/ heme binding / iron ion binding / monooxygenase/ oxygen binding | chr3:	2.586461
26.1	misc.cytochrome P450	kanlowctg47529_s_at	Transcript	very weakly similar to (85.5) loc_os04g10160 12004.m06268 protein cytochrome P450 CYP99A1, putative, expressed no original description	2.5708113
26.1	misc.cytochrome P450	ap13ctg11976_s_at	Transcript	very weakly similar to (87.8) C71C2_MAIZE Cytochrome P450 71C2 (EC 1.14.-.-) (Benzoxazineless 3) - Zea mays (Maize)weakly similar to (104) loc_os06g22020 120	1.6006612
26.1	misc.cytochrome P450	ap13itg70557_s_at	Transcript	weakly similar to (124) AT1G01190 | Symbols: CYP78A8 | CYP78A8; electron carrier/ heme binding / iron ion binding / monooxygenase/ oxygen binding | chr1:83	-1.4512961
26.1	misc.cytochrome P450	kanlowctg21384_x_at	Transcript	weakly similar to (123) AT3G26160 | Symbols: CYP71B17 | CYP71B17; electron carrier/ heme binding / iron ion binding / monooxygenase/ oxygen binding | chr3:	2.4542496
26.1	misc.cytochrome P450	ap13itg67659_at	Transcript	weakly similar to (122) AT1G11610 | Symbols: CYP71A18 | CYP71A18; electron carrier/ heme binding / iron ion binding / monooxygenase/ oxygen binding | chr1:	1.6206986
26.1	misc.cytochrome P450	ap13ctg18744_at	Transcript	weakly similar to (157) AT2G46950 | Symbols: CYP709B2 | CYP709B2; electron carrier/ heme binding / iron ion binding / monooxygenase/ oxygen binding | chr2:1	-1.2985504
26.11	misc.alcohol dehydrogenases	ap13itg56240_at	Transcript	weakly similar to (156) AT4G22110 | Symbols: | alcohol dehydrogenase, putative | chr4:11711422-11713946 REVERSEweakly similar to (101) ADHX_ORYSA Alcoh	2.606027
26.12	misc.peroxidases	ap13ctg09437_at	Transcript	very weakly similar to (89.0) PER2_ORYSA Peroxidase 2 precursor (EC 1.11.1.7) - Oryza sativa (Rice)very weakly similar to (91.3) loc_os07g48040 12007.m09019 pr	4.4435215
26.12	misc.peroxidases	ap13ctg01107_at	Transcript	weakly similar to (139) AT1G68850 | Symbols: | peroxidase, putative | chr1:25883806-25885177 REVERSEvery weakly similar to (80.9) PER2_ORYSA Peroxidase 2	1.0435702
26.12	misc.peroxidases	kanlowctg26782_s_at	Transcript	weakly similar to (155) AT5G05340 | Symbols: | peroxidase, putative | chr5:1579142-1580819 REVERSEweakly similar to (164) PER2_ORYSA Peroxidase 2 precurs	-2.5861895
26.13	misc.acid and other phosphatases	kanlowctg21816_s_at	Transcript	weakly similar to (113) AT4G29260 | Symbols: | acid phosphatase class B family protein | chr4:14422310-14423409 REVERSEweakly similar to (122) VSPA_SOYBN	2.1954758
26.13	misc.acid and other phosphatases	ap13itg70120_at	Transcript	weakly similar to (128) AT4G29260 | Symbols: | acid phosphatase class B family protein | chr4:14422310-14423409 REVERSEweakly similar to (144) VSPA_SOYBN	1.8381319
26.13	misc.acid and other phosphatases	ap13ctg24864_s_at	Transcript	weakly similar to (132) AT3G18220 | Symbols: | phosphatidic acid phosphatase family protein / PAP2 family protein | chr3:6248355-6249799 FORWARDweakly si	1.2430536
26.16	misc.myrosinases-lectin-jacalin	ap13ctg22520_at	Transcript	weakly similar to (105) AT2G37710 | Symbols: RLK | RLK (receptor lectin kinase); kinase | chr2:15814934-15816961 REVERSEweakly similar to (191) loc_os09g095	2.909293
26.16	misc.myrosinases-lectin-jacalin	ap13itg61865_s_at	Transcript	weakly similar to (133) AT1G19715 | Symbols: | jacalin lectin family protein | chr1:6816934-6819244 REVERSEmoderately similar to (226) loc_os05g43240 12005	-1.1359279
26.16	misc.myrosinases-lectin-jacalin	kanlowctg44153_s_at	Transcript	weakly similar to (152) AT1G19715 | Symbols: | jacalin lectin family protein | chr1:6816934-6819244 REVERSEmoderately similar to (208) loc_os05g43240 12005	-1.0720758
26.16	misc.myrosinases-lectin-jacalin	ap13itg71141_at	Transcript	weakly similar to (105) AT4G02420 | Symbols: | lectin protein kinase, putative | chr4:1064363-1066372 REVERSEweakly similar to (150) loc_os12g26940 12012.m	2.509205
26.16	misc.myrosinases-lectin-jacalin	ap13ctg52756_s_at	Transcript	weakly similar to (144) AT3G53810 | Symbols: | lectin protein kinase, putative | chr3:19933153-19935186 REVERSEmoderately similar to (221) loc_os07g03900 1	1.3268741
26.16	misc.myrosinases-lectin-jacalin	othswctg17780_at	Transcript	weakly similar to (113) AT1G19715 | Symbols: | jacalin lectin family protein | chr1:6816934-6819244 REVERSEweakly similar to (162) loc_os05g43240 12005.m08	-1.0945718
26.16	misc.myrosinases-lectin-jacalin	ap13ctg08614_s_at	Transcript	weakly similar to (135) loc_os07g03900 12007.m04860 protein lectin-like receptor kinase 7, putative no original description	1.2371218
26.16	misc.myrosinases-lectin-jacalin	ap13itg69554_at	Transcript	weakly similar to (123) AT4G19840 | Symbols: ATPP2-A1, ATPP2-A1, PP2-A1 | ATPP2-A1; carbohydrate binding | chr4:10774336-10775701 FORWARDweakly simil	-1.1787097
26.17	misc.dynamin	alamoctg05018_s_at	Transcript	weakly similar to (162) AT3G60190 | Symbols: ADL4, ADLP2, EDR3, DRP1E, ADL1E | ADL1E (ARABIDOPSIS DYNAMIN-LIKE 1E); GTP binding / GTPase | chr3:222443	1.7328136
26.19	misc.plastocyanin-like	ap13itg68926_at	Transcript	very weakly similar to (94.0) AT2G32300 | Symbols: UCC1 | UCC1 (UCLACYANIN 1); copper ion binding / electron carrier | chr2:13722510-13723464 FORWARDwea	2.5588279
26.21	misc.protease inhibitor/seed storage/lipid transfer protein (LTP) family	kanlowctg30642_s_at	Transcript	very weakly similar to (84.7) AT3G22142 | Symbols: | structural constituent of cell wall | chr3:7803604-7808046 REVERSEweakly similar to (120) loc_os04g52260	-1.1937379
26.22	misc.short chain dehydrogenase/reductase (SDR)	ap13ctg04703_at	Transcript	weakly similar to (142) AT5G04900 | Symbols: | short-chain dehydrogenase/reductase (SDR) family protein | chr5:1434826-1437194 FORWARDweakly similar to (	1.2028159
26.22	misc.short chain dehydrogenase/reductase (SDR)	ap13ctg39602_s_at	Transcript	weakly similar to (121) loc_os04g45000 12004.m35391 protein short-chain dehydrogenase/reductase SDR, putative, expressed no original description	1.6323588
26.22	misc.short chain dehydrogenase/reductase (SDR)	ap13itg69277_at	Transcript	weakly similar to (112) loc_os04g44924 12004.m101862 protein short-chain dehydrogenase/reductase SDR, putative, expressed no original description	3.1048453
26.24	misc.GCN5-related N-acetyltransferase	ap13itg61578_at	Transcript	weakly similar to (128) AT2G32030 | Symbols: | GCN5-related N-acetyltransferase (GNAT) family protein | chr2:13632675-13633241 REVERSEweakly similar to (1	4.6877933
26.28	misc.GDSL-motif lipase	ap13ctg04366_s_at	Transcript	weakly similar to (172) AT1G54790 | Symbols: | GDSL-motif lipase/hydrolase family protein | chr1:20441124-20443997 REVERSEvery weakly similar to (94.4) EST_	-1.082419
26.28	misc.GDSL-motif lipase	kanlowctg08094_s_at	Transcript	very weakly similar to (97.8) AT5G45910 | Symbols: | GDSL-motif lipase/hydrolase family protein | chr5:18620420-18622264 REVERSEweakly similar to (172) loc_	-1.2245063
26.28	misc.GDSL-motif lipase	ap13itg43227_s_at	Transcript	weakly similar to (102) AT5G22810 | Symbols: | GDSL-motif lipase, putative | chr5:7621568-7623367 FORWARDweakly similar to (150) loc_os06g05630 12006.m	-1.0792964
26.28	misc.GDSL-motif lipase	ap13itg64098-rc_at	Transcript	weakly similar to (134) loc_os06g06230 12006.m05349 protein carboxylic ester hydrolase/ hydrolase, acting on ester bonds, putative no original description	2.2048423
26.28	misc.GDSL-motif lipase	kanlowctg37283_s_at	Transcript	weakly similar to (128) AT3G16370 | Symbols: | GDSL-motif lipase/hydrolase family protein | chr3:5556928-5558351 FORWARDweakly similar to (188) loc_os06g	-2.0885303

Major CHO

BinCode	BinName	id	type	description	Se/60 (log2)
2.1.2.1	major CHO metabolism.synthesis.starch.AGPase	ap13ctg08222_s_at	Transcript	weakly similar to (115) AT5G19220 | Symbols: ADG2, APL1 | APL1 (ADP GLUCOSE PYROPHOSPHORYLASE LARGE SUBUNIT 1); glucose-1-phosphate adenylyltransfe	2.8284266
2.1.2.2	major CHO metabolism.synthesis.starch.starch synthase	kanlowctg41507_at	Transcript	very weakly similar to (80.5) SSY3_SOLTU Soluble starch synthase 3, chloroplast precursor (EC 2.4.1.21) (SS III) (Soluble starch synthase III) - Solanum tuberosum (P	-1.247854

2.1.2.3	major CHO metabolism.synthesis.starch.starch branching	othswslt23495_s_at	Transcript	weakly similar to (115) GLGB_ORYSA 1,4-alpha-glucan branching enzyme, chloroplast precursor (EC 2.4.1.18) (Starch branching enzyme) (Q-enzyme) - Oryza sativa	2.107312
2.1.2.3	major CHO metabolism.synthesis.starch.starch branching	ap13.12703.m00018_s_	Transcript	weakly similar to (117) AT5G03650 | Symbols: SBE2.2 | SBE2.2 (starch branching enzyme 2.2); 1,4-alpha-glucan branching enzyme | chr5:931924-937470 FORWAI	2.4501665
2.1.2.5	major CHO metabolism.synthesis.starch.transporter	ap13ctg04140_at	Transcript	weakly similar to (114) AT3G08580 | Symbols: AAC1 | AAC1 (ADP/ATP CARRIER 1); ATP:ADP antiporter/ binding | chr3:2605706-2607030 REVERSEweakly similar t	1.9033846
2.2.2.2	major CHO metabolism.degradation.starch.starch phosphorylase	ap13ctg03321_s_at	Transcript	weakly similar to (119) AT3G29320 | Symbols: | glucan phosphorylase, putative | chr3:11252871-11257587 FORWARDweakly similar to (120) PHSL_IPOBA Alpha-	2.940816
2.2.2.2	major CHO metabolism.degradation.starch.starch phosphorylase	ap13ctg01611-1_s_at	Transcript	moderately similar to (304) AT3G29320 | Symbols: | glucan phosphorylase, putative | chr3:11252871-11257587 FORWARDmoderately similar to (306) PHSL1_SO	2.7809234
2.2.2.2	major CHO metabolism.degradation.starch.starch phosphorylase	othswctg11635_s_at	Transcript	weakly similar to (151) AT3G46970 | Symbols: ATPHS2, PHS2 | PHS2 (ALPHA-GLUCAN PHOSPHORYLASE 2); phosphorylase/ transferase, transferring glycosyl group	2.6235723

List of significantly DEGs involved in hormone, misc and major CHO pathway in Se/Mo comparison

Hormone

BinCode	BinName	id	type	description	Se/Mo (log2)
17.1.1	hormone metabolism.abscisic acid.synthesis-degradation	ap13ctg31837_s_at	Transcript	weakly similar to (155) AT5G20960 | Symbols: AAO1, AO1, ATAO, ATAO1 | AAO1 (ARABIDOPSIS ALDEHYDE OXIDASE 1); aldehyde oxidase/ indole-3-acetaldehyde	2.250
17.1.1	hormone metabolism.abscisic acid.synthesis-degradation	kanlowctg13272_s_at	Transcript	weakly similar to (152) AT5G20960 | Symbols: AAO1, AO1, ATAO, ATAO1 | AAO1 (ARABIDOPSIS ALDEHYDE OXIDASE 1); aldehyde oxidase/ indole-3-acetaldehyde	-1.074
17.2.3	hormone metabolism.auxin.induced-regulated-responsive-activated	kanlowctg33522_at	Transcript	weakly similar to (138) AT1G60730 | Symbols: | aldo/keto reductase family protein | chr1:22358613-22360082 REVERSEweakly similar to (106) A115_TOBAC Aux	1.928
17.2.3	hormone metabolism.auxin.induced-regulated-responsive-activated	ap13ctg17086_at	Transcript	weakly similar to (103) IAA6_ORYSA Auxin-responsive protein IAA6 (Indoleacetic acid-induced protein 6) - Oryza sativa (Rice)weakly similar to (103) loc_os01g538	1.630
17.5.1.2	hormone metabolism.ethylene.synthesis-degradation.1-aminocyclopro	ap13itg55664_at	Transcript	very weakly similar to (86.3) AT1G62380 | Symbols: ACO2, ATACO2 | ACO2 (ACC OXIDASE 2); 1-aminocyclopropane-1-carboxylate oxidase | chr1:23082340-23084	2.119
17.6.1.1	hormone metabolism.gibberelin.synthesis-degradation.copalyl diphos	othswctg07572_at	Transcript	weakly similar to (105) loc_os02g17780 12002.m07022 protein ent-kaurene synthase A, chloroplast precursor, putative, expressed no original description	1.957
17.7.1.5	hormone metabolism.jasmonate.synthesis-degradation.12-Oxo-PDA-re	kanlowctg23007_at	Transcript	weakly similar to (176) AT1G76690 | Symbols: OPR2, ATOPR2 | OPR2 | chr1:28778976-28780355 FORWARDmoderately similar to (241) loc_os06g11210 12006.m	5.748
17.7.1.5	hormone metabolism.jasmonate.synthesis-degradation.12-Oxo-PDA-re	ap13itg71073_s_at	Transcript	weakly similar to (110) AT1G76690 | Symbols: OPR2, ATOPR2 | OPR2 | chr1:28778976-28780355 FORWARDweakly similar to (167) loc_os06g11210 12006.m058	4.396
17.7.1.5	hormone metabolism.jasmonate.synthesis-degradation.12-Oxo-PDA-re	ap13itg67010_s_at	Transcript	weakly similar to (115) AT1G76680 | Symbols: OPR1, ATOPR1 | OPR1; 12-oxophytodienoate reductase | chr1:28776982-28778271 FORWARDweakly similar to (1	3.112
17.7.1.5	hormone metabolism.jasmonate.synthesis-degradation.12-Oxo-PDA-re	ap13itg69033_at	Transcript	weakly similar to (162) AT1G76690 | Symbols: OPR2, ATOPR2 | OPR2 | chr1:28778976-28780355 FORWARDmoderately similar to (220) loc_os06g11290 12006.m	2.429

Misc

BinCode	BinName	id	type	description	Se/Mo (log2)
26.1	misc.misc2	ap13itg58554_at	Transcript	very weakly similar to (85.9) loc_os05g33110 12005.m083715 protein endo-1,3;1,4-beta-D-glucanase precursor, putative, expressed no original description	3.1168656
26.1	misc.misc2	ap13itg73544_at	Transcript	weakly similar to (140) loc_os10g35500 12010.m06374 protein epoxide hydrolase, putative, expressed no original description	2.049936
26.1	misc.misc2	ap13ctg12203_at	Transcript	weakly similar to (112) AT4G19185 | Symbols: | integral membrane family protein | chr4:10489201-10491488 REVERSEweakly similar to (157) loc_os06g49500 1	1.7140203
26.2	misc.UDP glucosyl and glucoronyl transferases	ap13itg75785rc_at	Transcript	moderately similar to (202) AT1G05680 | Symbols: | UDP-glucoronosyl/UDP-glucosyl transferase family protein | chr1:1703196-1704639 REVERSEweakly similar t	2.1618404
26.2	misc.UDP glucosyl and glucoronyl transferases	ap13itg74837_at	Transcript	very weakly similar to (95.1) AT1G01390 | Symbols: | UDP-glucoronosyl/UDP-glucosyl transferase family protein | chr1:148319-149761 REVERSEweakly similar to (	2.4085305
26.2	misc.UDP glucosyl and glucoronyl transferases	kanlowctg14906_s_at	Transcript	weakly similar to (119) loc_os01g45110 12001.m10717 protein cytokinin-O-glucosyltransferase 1, putative, expressed no original description	2.1462197
26.2	misc.UDP glucosyl and glucoronyl transferases	ap13itg47952_s_at	Transcript	weakly similar to (117) loc_os03g55050 12003.m10421 protein cytokinin-O-glucosyltransferase 2, putative, expressed no original description	2.346382
26.2	misc.UDP glucosyl and glucoronyl transferases	ap13ctg09529_at	Transcript	weakly similar to (162) AT1G05680 | Symbols: | UDP-glucoronosyl/UDP-glucosyl transferase family protein | chr1:1703196-1704639 REVERSEweakly similar to (1	3.4648933
26.3	misc.gluco-, galacto- and mannosidases	kanlowctg22294_at	Transcript	weakly similar to (137) loc_os01g16310 12001.m08227 protein glycosyl hydrolases family 31 protein, expressed no original description	3.0952632
26.3	misc.gluco-, galacto- and mannosidases	kanlowctg08905_s_at	Transcript	weakly similar to (108) loc_os05g30350 12005.m27797 protein non-cyanogenic beta-glucosidase precursor, putative, expressed no original description	2.0905576
26.3	misc.gluco-, galacto- and mannosidases	kanlowctg19994_s_at	Transcript	very weakly similar to (100) AT4G21760 | Symbols: BGLU47 | BGLU47 (Beta-glucosidase 47); catalytic/ cation binding / hydrolase, hydrolyzing O-glycosyl compour	3.0748446
26.3	misc.gluco-, galacto- and mannosidases	ap13itg52053_s_at	Transcript	very weakly similar to (82.0) AT1G61810 | Symbols: BGLU45 | BGLU45 (BETA-GLUCOSIDASE 45); catalytic/ cation binding / hydrolase, hydrolyzing O-glycosyl comp	2.22551
26.3.4	misc.gluco-, galacto- and mannosidases.endoglucanase	kanlowctg19895_x_at	Transcript	weakly similar to (152) AT5G49720 | Symbols: ATGH9A1, DEC, KOR, RSW2, IRX2, KOR1 | ATGH9A1 (ARABIDOPSIS THALIANA GLYCOSYL HYDROLASE 9A1); cellulase	2.2666078
26.4	misc.beta 1,3 glucan hydrolases	ap13itg58279_s_at	Transcript	weakly similar to (122) AT3G07320 | Symbols: | glycosyl hydrolase family 17 protein | chr3:2332324-2333925 REVERSEweakly similar to (177) loc_os09g32550 12	1.7381021
26.4.1	misc.beta 1,3 glucan hydrolases.glucan endo-1,3-beta-glucosidase	kanlowctg10503rc_s_a	Transcript	weakly similar to (102) GUB2_HORVU Lichenase-2 precursor (EC 3.2.1.73) (Lichenase II) ((1->3,1->4)-beta-glucanase isoenzyme EII	-1.907923
26.4.1	misc.beta 1,3 glucan hydrolases.glucan endo-1,3-beta-glucosidase	ap13itg70254_at	Transcript	weakly similar to (113) AT4G16260 | Symbols: | catalytic/ cation binding / hydrolase, hydrolyzing O-glycosyl compounds | chr4:9200180-9201441 REVERSEweakly	1.7229776
26.6	misc.O-methyl transferases	ap13ctg23081_s_at	Transcript	very weakly similar to (98.2) AT3G12270 | Symbols: ATPRMT3, PRMT3 | PRMT3 (PROTEIN ARGININE METHYLTRANSFERASE 3); methyltransferase/ zinc ion binding	1.9541873
26.7	misc.oxidases - copper, flavone etc	kanlowctg47289_s_at	Transcript	weakly similar to (107) loc_os03g05900 12003.m101192 protein monooxygenase, putative, expressed no original description	1.5915792
26.7	misc.oxidases - copper, flavone etc	ap13ctg02095_at	Transcript	weakly similar to (103) loc_os09g37620 12009.m06709 protein monooxygenase/ oxidoreductase, putative, expressed no original description	1.8820726
26.9	misc.glutathione S transferases	ap13itg52395_s_at	Transcript	very weakly similar to (89.7) AT1G10370 | Symbols: GST30, ATGSTU17, GST30B, ERD9 | ERD9 (EARLY-RESPONSIVE TO DEHYDRATION 9); glutathione transferase |	2.0166132
26.9	misc.glutathione S transferases	ap13ctg23895_at	Transcript	weakly similar to (116) GSTU6_ORYSA Probable glutathione S-transferase GSTU6 (EC 2.5.1.18) (28 kDa cold-induced protein) - Oryza sativa (Rice)weakly similar to	1.9411697
26.1	misc.cytochrome P450	ap13ctg15166_s_at	Transcript	moderately similar to (233) AT2G32440 | Symbols: KAO2, CYP88A4 | KAO2 (ENT-KAURENOIC ACID HYDROXYLASE 2); ent-kaurenoate oxidase/ oxygen binding | ch	-1.3691137
26.1	misc.cytochrome P450	ap13ctg31838_s_at	Transcript	weakly similar to (102) loc_os02g30100 12002.m08153 protein cytochrome P450 81E1, putative, expressed no original description	2.0546823
26.1	misc.cytochrome P450	ap13ctg15593_s_at	Transcript	very weakly similar to (89.7) AT3G14620 | Symbols: CYP72A8 | CYP72A8; electron carrier/ heme binding / iron ion binding / monooxygenase/ oxygen binding | chr	3.6020677
26.1	misc.cytochrome P450	kanlowctg12693-1_s_a	Transcript	weakly similar to (108) AT1G12740 | Symbols: CYP87A2 | CYP87A2; electron carrier/ heme binding / iron ion binding / monooxygenase/ oxygen binding | chr1:43	1.1912225
26.12	misc.peroxidases	ap13ctg09437_at	Transcript	very weakly similar to (89.0) PER2_ORYSA Peroxidase 2 precursor (EC 1.11.1.7) - Oryza sativa (Rice)very weakly similar to (91.3) loc_os07g48040 12007.m09019 pro	2.2950435
26.13	misc.acid and other phosphatases	kanlowctg21816_s_at	Transcript	weakly similar to (113) AT4G29260 | Symbols: | acid phosphatase class B family protein | chr4:14422310-14423409 REVERSEweakly similar to (122) VSPA_SOYBN	1.9629114
26.13	misc.acid and other phosphatases	ap13itg70120_at	Transcript	weakly similar to (128) AT4G29260 | Symbols: | acid phosphatase class B family protein | chr4:14422310-14423409 REVERSEweakly similar to (144) VSPA_SOYBN	2.079594
26.16	misc.myrosinases-lectin-jacalin	ap13ctg22520_at	Transcript	weakly similar to (105) AT2G37710 | Symbols: RLK | RLK (receptor lectin kinase); kinase | chr2:15814934-15816961 REVERSEweakly similar to (191) loc_os09g09	1.9184065
26.19	misc.plastocyanin-like	ap13itg68926_at	Transcript	very weakly similar to (94.0) AT2G32300 | Symbols: UCC1 | UCC1 (UCLACYANIN 1); copper ion binding / electron carrier | chr2:13722510-13723464 FORWARDwea	1.6645914
26.22	misc.short chain dehydrogenase/reductase (SDR)	ap13itg69277_at	Transcript	weakly similar to (112) loc_os04g44924 12004.m101862 protein short-chain dehydrogenase/reductase SDR, putative, expressed no original description	2.5772862

Major CHO

BinCode	BinName	id	type	description	Se/Mo (log2)
2.1.2.1	major CHO metabolism.synthesis.starch.AGPase	ap13ctg08222_s_at	Transcript	weakly similar to (115) AT5G19220 | Symbols: ADG2, APL1 | APL1 (ADP GLUCOSE PYROPHOSPHORYLASE LARGE SUBUNIT 1); glucose-1-phosphate adenylyltransfe	1.7421138
2.1.2.3	major CHO metabolism.synthesis.starch.starch branching	othswslt23495_s_at	Transcript	weakly similar to (115) GLGB_ORYSA 1,4-alpha-glucan branching enzyme, chloroplast precursor (EC 2.4.1.18) (Starch branching enzyme) (Q-enzyme) - Oryza sativa	2.1660018
2.1.2.3	major CHO metabolism.synthesis.starch.starch branching	ap13.12703.m00018_s_	Transcript	weakly similar to (117) AT5G03650 | Symbols: SBE2.2 | SBE2.2 (starch branching enzyme 2.2); 1,4-alpha-glucan branching enzyme | chr5:931924-937470 FORWAI	2.2696276
2.1.2.5	major CHO metabolism.synthesis.starch.transporter	ap13ctg04140_at	Transcript	weakly similar to (114) AT3G08580 | Symbols: AAC1 | AAC1 (ADP/ATP CARRIER 1); ATP:ADP antiporter/ binding | chr3:2605706-2607030 REVERSEweakly similar t	1.5862548
2.2.2.2	major CHO metabolism.degradation.starch.starch phosphorylase	ap13ctg03321_s_at	Transcript	weakly similar to (119) AT3G29320 | Symbols: | glucan phosphorylase, putative | chr3:11252871-11257587 FORWARDweakly similar to (120) PHSL_IPOBA Alpha-	2.5334692
2.2.2.2	major CHO metabolism.degradation.starch.starch phosphorylase	ap13ctg01611-1_s_at	Transcript	moderately similar to (304) AT3G29320 | Symbols: | glucan phosphorylase, putative | chr3:11252871-11257587 FORWARDmoderately similar to (306) PHSL1_SO	2.3238447
2.2.2.2	major CHO metabolism.degradation.starch.starch phosphorylase	othswctg11635_s_at	Transcript	weakly similar to (151) AT3G46970 | Symbols: ATPHS2, PHS2 | PHS2 (ALPHA-GLUCAN PHOSPHORYLASE 2); phosphorylase/ transferase, transferring glycosyl group	2.7006457

Table S6. List of significantly DEGs related to biotic and abiotic stress pathways in four comparisons

List of significantly DEGs involved in hormone metabolism, regulation of trancription factor and abiotic stress pathway in Exp/Ctrl comparison

Hormone

BinCode	BinName	id	type	description	Exp/Ctrl (log2)
17.7.1.2	hormone metabolism.jasmonat	ap13itg70317_at	Transcript	weakly similar to (143) AT3G45140 | Symbols: LOX2, ATLOX2 | LOX2 (LIPOXYGENASE 2); lipoxygenase | chr3:16525437-16529233 FORWARDweakly similar to (199) LOX2	2.5209846
17.7.1.5	hormone metabolism.jasmonat	kanlowctg23007_at	Transcript	weakly similar to (176) AT1G76690 | Symbols: OPR2, ATOPR2 | OPR2 | chr1:28778976-28780355 FORWARDmoderately similar to (241) loc_os06g11210 12006.m05838	4.7242365
17.7.1.5	hormone metabolism.jasmonat	ap13itg71073_s_at	Transcript	weakly similar to (110) AT1G76690 | Symbols: OPR2, ATOPR2 | OPR2 | chr1:28778976-28780355 FORWARDweakly similar to (167) loc_os06g11210 12006.m05838 prot	3.2742903
17.7.1.5	hormone metabolism.jasmonat	ap13itg67010_s_at	Transcript	weakly similar to (115) AT1G76680 | Symbols: OPR1, ATOPR1 | OPR1; 12-oxophytodienoate reductase | chr1:28776982-28778271 FORWARDweakly similar to (128) loc_	2.168549

Transcription factor

BinCode	BinName	id	type	description	Exp/Ctrl (log2)
27.3.26	RNA.regulation of transcription	ap13ctg22648_at	Transcript	very weakly similar to (81.6) AT4G39250 | Symbols: ATRL1 | ATRL1 (ARABIDOPSIS RAD-LIKE 1); DNA binding / transcription factor | chr4:18271457-18271857 REVERSEwe	-1.7655948
27.3.26	RNA.regulation of transcription	ap13itg63308-rc_at	Transcript	very weakly similar to (88.6) AT1G75250 | Symbols: ATRL6 | ATRL6 (ARABIDOPSIS RAD-LIKE 6); transcription factor | chr1:28244463-28245453 REVERSEweakly similar to (	-2.389372
27.3.35	RNA.regulation of transcription	ap13itg72122_at	Transcript	very weakly similar to (89.7) AT1G08320 | Symbols: | bZIP family transcription factor | chr1:2622113-2627451 REVERSEvery weakly similar to (80.5) TGA21_TOBAC TGAC	-2.9408507

Abiotic stress

BinCode	BinName	id	type	description	Exp/Ctrl (log2)
20.2.1	stress.abiotic.heat	ap13itg66986_at	Transcript	weakly similar to (164) AT3G12580 | Symbols: HSP70 | HSP70 (heat shock protein 70); ATP binding | chr3:3991487-3993689 REVERSEweakly similar to (163) HSP7C_PETI	1.7140276
20.2.1	stress.abiotic.heat	ap13itg70020_at	Transcript	weakly similar to (102) loc_os01g65480 12001.m12659 protein dnaJ protein homolog 1, putative, expressed no original description	-1.2925655
20.2.2	stress.abiotic.cold	kanlowctg06768_at	Transcript	very weakly similar to (89.0) LTI6A_ORYSA Hydrophobic protein LTI6A (Low temperature-induced protein 6A) - Oryza sativa (Rice)very weakly similar to (89.0) loc_os07g4	-2.341828
20.2.3	stress.abiotic.drought/salt	ap13ctg19249_at	Transcript	very weakly similar to (92.4) loc_os01g73960 12001.m43394 protein fb2, putative, expressed no original description	-1.1559153

List of significantly DEGs involved in abiotic stress, signalling, regulation of trancription factor and cell wall pathway in Mo/60 comparison

Abiotic stress

BinCode	BinName	id	type	description	Mo/60 (log2)
20.2.1	stress.abiotic.heat	ap13ctg12560_s_at	Transcript	weakly similar to (149) loc_os02g32590 12002.m33703 protein heat shock factor protein 2, putative, expressed no original description	-1.1621747
20.2.1	stress.abiotic.heat	ap13itg70020_at	Transcript	weakly similar to (102) loc_os01g65480 12001.m12659 protein dnaJ protein homolog 1, putative, expressed no original description	-1.47846
20.2.2	stress.abiotic.cold	kanlowctg06768_at	Transcript	very weakly similar to (89.0) LTI6A_ORYSA Hydrophobic protein LTI6A (Low temperature-induced protein 6A) - Oryza sativa (Rice)very weakly similar to (89.0) loc_os07g4	-3.0117598
20.2.3	stress.abiotic.drought/salt	ap13ctg19249_at	Transcript	very weakly similar to (92.4) loc_os01g73960 12001.m43394 protein fb2, putative, expressed no original description	-1.3544257
20.2.3	stress.abiotic.drought/salt	ap13ctg30618_at	Transcript	weakly similar to (129) AT2G18250 | Symbols: ATCOAD | ATCOAD (4-phosphopantetheine adenylyltransferase); nucleotidyltransferase/ pantetheine-phosphate adenylyl	-1.3595558
20.2.4	stress.abiotic.touch/wounding	kanlowctg10783_s_at	Transcript	very weakly similar to (85.9) loc_os04g54280 12004.m10320 protein retrotransposon protein, putative, unclassified no original description	-1.1250397
20.2.4	stress.abiotic.touch/wounding	ap13itg73873_s_at	Transcript	moderately similar to (201) loc_os05g27580 12005.m07046 protein wound-induced protein WI12 containing protein no original description	-1.0588257
20.2.4	stress.abiotic.touch/wounding	kanlowctg09577_s_at	Transcript	weakly similar to (164) loc_os05g27580 12005.m07046 protein wound-induced protein WI12 containing protein no original description	-1.2653005
20.2.4	stress.abiotic.touch/wounding	kanlowctg16979_s_at	Transcript	very weakly similar to (92.0) loc_os04g54300 12004.m78995 protein wound induced protein, putative, expressed no original description	-1.3814937
20.2.4	stress.abiotic.touch/wounding	ap13ctg24346_at	Transcript	very weakly similar to (86.3) AT4G10270 | Symbols: | wound-responsive family protein | chr4:6374805-6375077 FORWARDweakly similar to (115) loc_os04g54280 1200	-1.7902399
20.2.4	stress.abiotic.touch/wounding	ap13itg39065_s_at	Transcript	very weakly similar to (80.5) AT4G10265 | Symbols: | wound-responsive protein, putative | chr4:6373226-6373477 REVERSEweakly similar to (113) loc_os04g54280 120	-1.7631652
20.2.4	stress.abiotic.touch/wounding	ap13itg60359_s_at	Transcript	weakly similar to (106) loc_os04g54280 12004.m10320 protein retrotransposon protein, putative, unclassified no original description	-1.0302726
20.2.4	stress.abiotic.touch/wounding	ap13ctg59032_at	Transcript	very weakly similar to (88.6) loc_os04g54280 12004.m10320 protein retrotransposon protein, putative, unclassified no original description	-1.8604492

Signalling

BinCode	BinName	id	type	description	Mo/60 (log2)
30.1	signalling.in sugar and nutrient	ap13itg74992_at	Transcript	weakly similar to (117) loc_os02g54640 12002.m10493 protein glutamate receptor 2.9 precursor, putative, expressed no original description	1.119977
30.2.11	signalling.receptor kinases.leuc	ap13itg61870_at	Transcript	weakly similar to (116) loc_os11g07240 12011.m04920 protein receptor protein kinase CLAVATA1 precursor, putative, expressed no original description	1.1316558
30.2.11	signalling.receptor kinases.leuc	othswctg12119_at	Transcript	very weakly similar to (80.1) AT3G47580 | Symbols: | leucine-rich repeat transmembrane protein kinase, putative | chr3:17532687-17535810 FORWARDweakly similar to	1.0568756
30.2.11	signalling.receptor kinases.leuc	othswctg27954_s_at	Transcript	weakly similar to (132) loc_os02g40180 12002.m09104 protein receptor-like protein kinase precursor, putative, expressed no original description	2.0184202
30.2.11	signalling.receptor kinases.leuc	ap13ctg04179_at	Transcript	weakly similar to (105) loc_os01g65650 12001.m12674 protein receptor-like protein kinase 5 precursor, putative, expressed no original description	-1.9957428
30.2.11	signalling.receptor kinases.leuc	kanlowctg42715_at	Transcript	weakly similar to (107) AT3G47570 | Symbols: | leucine-rich repeat transmembrane protein kinase, putative | chr3:17527611-17530748 FORWARDmoderately similar to	2.03235
30.2.11	signalling.receptor kinases.leuc	othswctg19246_at	Transcript	very weakly similar to (84.0) AT3G47110 | Symbols: | leucine-rich repeat transmembrane protein kinase, putative | chr3:17347103-17350296 REVERSEweakly similar to (	1.51942
30.2.11	signalling.receptor kinases.leuc	ap13ctg16678_s_at	Transcript	very weakly similar to (81.3) AT5G48380 | Symbols: | leucine-rich repeat family protein / protein kinase family protein | chr5:19604584-19606532 REVERSEweakly similar	1.0412167
30.2.11	signalling.receptor kinases.leuc	othswctg17938_at	Transcript	weakly similar to (115) AT2G15080 | Symbols: AtRLP19 | AtRLP19 (Receptor Like Protein 19); kinase/ protein binding | chr2:6533764-6536715 FORWARDweakly similar t	1.6272969
30.2.11	signalling.receptor kinases.leuc	othswctg17630_at	Transcript	weakly similar to (174) loc_os02g40240 12002.m33414 protein leucine-rich repeat receptor protein kinase EXS precursor, putative, expressed no original description	1.6988038
30.2.11	signalling.receptor kinases.leuc	ap13ctg50688_s_at	Transcript	weakly similar to (105) loc_os04g15630 12004.m06749 protein leucine-rich repeat receptor protein kinase EXS precursor, putative no original description	1.5420277
30.2.11	signalling.receptor kinases.leuc	kanlowctg19800_s_at	Transcript	very weakly similar to (90.9) loc_os02g40180 12002.m09104 protein receptor-like protein kinase precursor, putative, expressed no original description	1.3183337
30.2.11	signalling.receptor kinases.leuc	othswslt25490_at	Transcript	weakly similar to (114) AT3G47570 | Symbols: | leucine-rich repeat transmembrane protein kinase, putative | chr3:17527611-17530748 FORWARDmoderately similar to	1.2138089
30.2.11	signalling.receptor kinases.leuc	ap13ctg03762_at	Transcript	weakly similar to (181) loc_os02g40240 12002.m33414 protein leucine-rich repeat receptor protein kinase EXS precursor, putative, expressed no original description	1.5610776
30.2.11	signalling.receptor kinases.leuc	othswctg18100_at	Transcript	weakly similar to (131) loc_os11g36190 12011.m080025 protein receptor-like protein kinase 5 precursor, putative, expressed no original description	1.3126502
30.2.16	signalling.receptor kinases.Cath	ap13ctg04617_at	Transcript	weakly similar to (143) loc_os06g22810 12006.m06934 protein protein kinase, putative, expressed no original description	-1.1393493
30.2.17	signalling.receptor kinases.DUF	ap13itg41137_at	Transcript	weakly similar to (142) loc_os09g39650 12009.m060077 protein ATP binding protein, putative, expressed no original description	2.0351868
30.2.17	signalling.receptor kinases.DUF	othswctg18108_at	Transcript	very weakly similar to (97.8) AT1G11330 | Symbols: | S-locus lectin protein kinase family protein | chr1:3810372-3813416 FORWARDweakly similar to (148) loc_os04g54	1.9088267
30.2.17	signalling.receptor kinases.DUF	ap13itg69009_at	Transcript	very weakly similar to (90.1) AT4G23160 | Symbols: | protein kinase family protein | chr4:12129485-12134086 FORWARDweakly similar to (166) loc_os05g39130 12005.	1.1350458
30.2.17	signalling.receptor kinases.DUF	ap13itg77404_s_at	Transcript	very weakly similar to (94.7) AT4G03230 | Symbols: | ATP binding / kinase/ protein kinase/ protein serine/threonine kinase/ protein tyrosine kinase/ sugar binding | chr4:	1.5196985
30.2.17	signalling.receptor kinases.DUF	ap13itg39900_s_at	Transcript	very weakly similar to (91.3) loc_os12g14480 12012.m05416 protein ATP binding protein, putative no original description	2.5227633
30.2.17	signalling.receptor kinases.DUF	ap13itg76323_at	Transcript	weakly similar to (116) loc_os01g02390 12001.m42838 protein TAK14, putative, expressed no original description	2.8866818
30.2.17	signalling.receptor kinases.DUF	ap13itg47888_s_at	Transcript	very weakly similar to (86.7) loc_os11g11780 12011.m05370 protein serine/threonine protein kinase, putative, expressed no original description	-1.1535704
30.2.17	signalling.receptor kinases.DUF	ap13ctg07687_at	Transcript	weakly similar to (159) loc_os01g02800 12001.m06920 protein receptor-like kinase ARK1AS, putative, expressed no original description	1.2190235
30.2.17	signalling.receptor kinases.DUF	ap13ctg48706_at	Transcript	very weakly similar to (98.6) AT4G23130 | Symbols: CRK5, RLK6 | CRK5 (CYSTEINE-RICH RLK5); kinase | chr4:12117688-12120134 REVERSEvery weakly similar to (82.8) NC	3.9346542
30.2.17	signalling.receptor kinases.DUF	ap13ctg11071_s_at	Transcript	weakly similar to (102) loc_os07g35290 12007.m07785 protein receptor-like serine-threonine protein kinase, putative, expressed no original description	1.346304

30.2.17	signalling.receptor kinases.DUF	ap13ctg22926_at	Transcript	very weakly similar to (90.5) AT4G23180 | Symbols: CRK10, RLK4 | CRK10 (CYSTEINE-RICH RLK10); ATP binding / kinase/ protein kinase/ protein serine/threonine kinase/ p	-1.065511
30.2.25	signalling.receptor kinases.wall	ap13ctg19859_at	Transcript	weakly similar to (142) loc_os04g43730 12004.m09332 protein OsWAK51 - OsWAK receptor-like protein kinase, expressed no original description	1.6219307
30.2.25	signalling.receptor kinases.wall	ap13ctg14697_s_at	Transcript	weakly similar to (162) loc_os04g03830 12004.m05691 protein OsWAK29 - OsWAK receptor-like protein kinase, expressed no original description	1.7394327
30.2.25	signalling.receptor kinases.wall	othswctg14972_at	Transcript	weakly similar to (130) loc_os02g42150 12002.m09252 protein OsWAK14 - OsWAK receptor-like protein kinase, expressed no original description	3.268809
30.2.25	signalling.receptor kinases.wall	alamoctg14172_at	Transcript	very weakly similar to (88.6) loc_os02g42150 12002.m09252 protein OsWAK14 - OsWAK receptor-like protein kinase, expressed no original description	1.109805
30.2.25	signalling.receptor kinases.wall	ap13ctg23391_at	Transcript	weakly similar to (116) loc_os03g62430 12003.m11119 protein OsWAK28 - OsWAK receptor-like protein kinase, expressed no original description	1.8729477
30.2.25	signalling.receptor kinases.wall	ap13itg48098_at	Transcript	very weakly similar to (94.4) AT1G21230 | Symbols: WAK5 | WAK5 (WALL ASSOCIATED KINASE 5); kinase/ protein serine/threonine kinase | chr1:7429980-7432346 FORW	-1.1851537
30.2.99	signalling.receptor kinases.misc	ap13itg77449-rc_at	Transcript	weakly similar to (138) loc_os05g44970 12005.m08622 protein senescence-induced receptor-like serine/threonine-protein kinase precursor, putative no original descript	1.7617186
30.2.99	signalling.receptor kinases.misc	ap13itg41137_at	Transcript	weakly similar to (142) loc_os09g39650 12009.m060077 protein ATP binding protein, putative, expressed no original description	2.0351868
30.2.99	signalling.receptor kinases.misc	kanlowctg38494_at	Transcript	very weakly similar to (95.9) AT2G28970 | Symbols: | leucine-rich repeat protein kinase, putative | chr2:12443919-12448163 FORWARDweakly similar to (165) loc_os09g	1.0940689
30.2.99	signalling.receptor kinases.misc	ap13itg58665_at	Transcript	weakly similar to (132) AT2G28970 | Symbols: | leucine-rich repeat protein kinase, putative | chr2:12443919-12448163 FORWARDweakly similar to (105) NORK_MEDTR	1.2050234
30.2.99	signalling.receptor kinases.misc	ap13itg48098_at	Transcript	very weakly similar to (94.4) AT1G21230 | Symbols: WAK5 | WAK5 (WALL ASSOCIATED KINASE 5); kinase/ protein serine/threonine kinase | chr1:7429980-7432346 FORW	-1.1851537
30.3	signalling.calcium	ap13itg66751_s_at	Transcript	weakly similar to (117) AT2G18750 | Symbols: | calmodulin-binding protein | chr2:8125827-8128363 FORWARDmoderately similar to (220) loc_os12g36920 12012.m07	1.5560796
30.3	signalling.calcium	kanlowctg04099_s_at	Transcript	weakly similar to (104) AT1G27770 | Symbols: ACA1, PEA1 | ACA1 (AUTO-INHIBITED CA2+-ATPASE 1); calcium channel/ calcium-transporting ATPase/ calmodulin binding	1.0539427
30.3	signalling.calcium	ap13ctg14744_s_at	Transcript	weakly similar to (128) loc_os12g36110 12012.m07401 protein calmodulin binding protein, putative, expressed no original description	2.738117
30.3	signalling.calcium	ap13ctg08973_s_at	Transcript	very weakly similar to (81.6) AT4G37640 | Symbols: ACA2 | ACA2 (CALCIUM ATPASE 2); calcium ion transmembrane transporter/ calcium-transporting ATPase/ calmoduli	1.1298444
30.5	signalling.G-proteins	kanlowctg44370_s_at	Transcript	very weakly similar to (91.3) loc_os06g02130 12006.m31954 protein guanyl nucleotide binding protein, putative, expressed no original description	1.6158048

Transcription factor

BinCode	BinName	id	type	description	Mo/60 (log2)
27.3.26	RNA.regulation of transcription	ap13ctg22648_at	Transcript	very weakly similar to (81.6) AT4G39250 | Symbols: ATRL1 | ATRL1 (ARABIDOPSIS RAD-LIKE 1); DNA binding / transcription factor | chr4:18271457-18271857 REVERSEwe	-1.3755075
27.3.26	RNA.regulation of transcription	ap13itg63308-rc_at	Transcript	very weakly similar to (88.6) AT1G75250 | Symbols: ATRL6 | ATRL6 (ARABIDOPSIS RAD-LIKE 6); transcription factor | chr1:28244463-28245453 REVERSEweakly similar to (	-2.4642951
27.3.26	RNA.regulation of transcription	ap13itg71040_at	Transcript	weakly similar to (106) loc_os01g09280 12001.m07553 protein myb-related transcription activator, putative, expressed no original description	-1.0700543

Cell wall

BinCode	BinName	id	type	description	Mo/60 (log2)
26.4.1	misc.beta 1,3 glucan hydrolases	kanlowctg10503rc_s_a	Transcript	weakly similar to (102) GUB2_HORVU Lichenase-2 precursor (EC 3.2.1.73) (Lichenase II) (Endo-beta-1,3-1,4 glucanase II) ((1->3,1->4)-beta-glucanase isoenzyme EII) (Fragr	1.4965644
26.4.1	misc.beta 1,3 glucan hydrolases	kanlowctg24512_s_at	Transcript	weakly similar to (106) AT3G57260 | Symbols: BGL2, PR2, BG2, PR-2 | BGL2 (BETA-1,3-GLUCANASE 2); cellulase/ glucan 1,3-beta-glucosidase/ hydrolase, hydrolyzing O-gl	2.3199854

List of significantly DEGs involved in hormone, regulation of trancription factor and glutathione S transferases, and signalling pathway in Se/60 comparison

Hormone

BinCode	BinName	id	type	description	Se/60 (log2)
17.7.1.2	hormone metabolism.jasmonat	ap13itg70317_at	Transcript	weakly similar to (143) AT3G45140 | Symbols: LOX2, ATLOX2 | LOX2 (LIPOXYGENASE 2); lipoxygenase | chr3:16525437-16529233 FORWARDweakly similar to (199) LOX2	2.7463124
17.7.1.5	hormone metabolism.jasmonat	kanlowctg23007_at	Transcript	weakly similar to (176) AT1G76690 | Symbols: OPR2, ATOPR2 | OPR2 | chr1:28778976-28780355 FORWARDmoderately similar to (241) loc_os06g11210 12006.m05838	5.94585
17.7.1.5	hormone metabolism.jasmonat	ap13itg71073_s_at	Transcript	weakly similar to (110) AT1G76690 | Symbols: OPR2, ATOPR2 | OPR2 | chr1:28778976-28780355 FORWARDweakly similar to (167) loc_os06g11210 12006.m05838 prot	4.575795
17.7.1.5	hormone metabolism.jasmonat	ap13itg67010_s_at	Transcript	weakly similar to (115) AT1G76680 | Symbols: OPR1, ATOPR1 | OPR1; 12-oxophytodienoate reductase | chr1:28776982-28778271 FORWARDweakly similar to (128) loc_	3.1269832
17.7.1.5	hormone metabolism.jasmonat	ap13itg69033_at	Transcript	weakly similar to (162) AT1G76690 | Symbols: OPR2, ATOPR2 | OPR2 | chr1:28778976-28780355 FORWARDmoderately similar to (220) loc_os06g11290 12006.m05846	2.3178062

Transcription factor

BinCode	BinName	id	type	description	Se/60 (log2)
27.3.26	RNA.regulation of transcription	ap13ctg22648_at	Transcript	very weakly similar to (81.6) AT4G39250 | Symbols: ATRL1 | ATRL1 (ARABIDOPSIS RAD-LIKE 1); DNA binding / transcription factor | chr4:18271457-18271857 REVERSEwe	-1.8795223
27.3.26	RNA.regulation of transcription	ap13itg63308-rc_at	Transcript	very weakly similar to (88.6) AT1G75250 | Symbols: ATRL6 | ATRL6 (ARABIDOPSIS RAD-LIKE 6); transcription factor | chr1:28244463-28245453 REVERSEweakly similar to (	-2.725983

Glutathione S transferases

BinCode	BinName	id	type	description	Se/60 (log2)
26.9	misc.glutathione S transferases	ap13ctg18609_at	Transcript	very weakly similar to (91.3) AT3G55040 | Symbols: GSTL2 | GSTL2 | chr3:20398718-20400305 REVERSEvery weakly similar to (97.4) IN21_MAIZE IN2-1 protein - Zea may	3.4394202
26.9	misc.glutathione S transferases	ap13itg59978_at	Transcript	very weakly similar to (82.0) GSTU6_ORYSA Probable glutathione S-transferase GSTU6 (EC 2.5.1.18) (28 kDa cold-induced protein) - Oryza sativa (Rice)weakly similar to (1	1.8530868
26.9	misc.glutathione S transferases	ap13itg52395_s_at	Transcript	very weakly similar to (89.7) AT1G10370 | Symbols: GST30, ATGSTU17, GST30B, ERD9 | ERD9 (EARLY-RESPONSIVE TO DEHYDRATION 9); glutathione transferase | chr1:33	2.6520345
26.9	misc.glutathione S transferases	ap13ctg23895_at	Transcript	weakly similar to (116) GSTU6_ORYSA Probable glutathione S-transferase GSTU6 (EC 2.5.1.18) (28 kDa cold-induced protein) - Oryza sativa (Rice)weakly similar to (116) lo	2.3023114
26.9	misc.glutathione S transferases	ap13itg64056_at	Transcript	weakly similar to (107) loc_os10g38340 12010.m06607 protein glutathione S-transferase GSTU6, putative, expressed no original description	2.9526143
26.9	misc.glutathione S transferases	ap13ctg24964_at	Transcript	very weakly similar to (93.6) AT3G62760 | Symbols: ATGSTF13 | ATGSTF13; glutathione transferase | chr3:23217425-23218246 REVERSEweakly similar to (135) GSTF4_M	1.6238625
26.9	misc.glutathione S transferases	ap13ctg16606_s_at	Transcript	very weakly similar to (90.5) AT3G55040 | Symbols: GSTL2 | GSTL2 | chr3:20398718-20400305 REVERSEweakly similar to (149) IN21_MAIZE IN2-1 protein - Zea mays (Ma	1.6981143
26.9	misc.glutathione S transferases	ap13itg54282_at	Transcript	very weakly similar to (92.4) AT3G62760 | Symbols: ATGSTF13 | ATGSTF13; glutathione transferase | chr3:23217425-23218246 REVERSEweakly similar to (164) GSTF4_M	1.7701411

Signalling

BinCode	BinName	id	type	description	Se/60 (log2)
30.1	signalling.in sugar and nutrient	ap13itg51186_at	Transcript	weakly similar to (111) AT5G11180 | Symbols: ATGLR2.6, GLR2.6 | ATGLR2.6; intracellular ligand-gated ion channel | chr5:3557261-3561575 REVERSEvery weakly similar	2.129366
30.1	signalling.in sugar and nutrient	othswctg22609_at	Transcript	very weakly similar to (85.1) AT5G27100 | Symbols: ATGLR2.1, GLR2.1 | ATGLR2.1; intracellular ligand-gated ion channel | chr5:9535160-9538311 REVERSEmoderately sir	2.047804
30.2.3	signalling.receptor kinases.leuc	kanlowctg45110_s_at	Transcript	weakly similar to (133) AT5G58300 | Symbols: | leucine-rich repeat transmembrane protein kinase, putative | chr5:23572821-23574871 FORWARDweakly similar to (179	2.0986495
30.2.11	signalling.receptor kinases.leuc	ap13itg61870_at	Transcript	weakly similar to (116) loc_os11g07240 12011.m04920 protein receptor protein kinase CLAVATA1 precursor, putative, expressed no original description	1.5433446
30.2.11	signalling.receptor kinases.leuc	kanlowctg40038_at	Transcript	very weakly similar to (88.2) loc_os02g13510 12002.m06597 protein receptor-like protein kinase 5 precursor, putative, expressed no original description	1.063403
30.2.11	signalling.receptor kinases.leuc	kanlowctg42715_at	Transcript	weakly similar to (107) AT3G47570 | Symbols: | leucine-rich repeat transmembrane protein kinase, putative | chr3:17527611-17530748 FORWARDmoderately similar to	1.6100112
30.2.11	signalling.receptor kinases.leuc	othswctg17938_at	Transcript	weakly similar to (115) AT2G15080 | Symbols: AtRLP19 | AtRLP19 (Receptor Like Protein 19); kinase/ protein binding | chr2:6533764-6536715 FORWARDweakly similar t	1.4178946
30.2.11	signalling.receptor kinases.leuc	ap13ctg31498_at	Transcript	very weakly similar to (85.1) loc_os06g36270 12006.m091721 protein receptor-like protein kinase 5 precursor, putative, expressed no original description	1.7436931
30.2.11	signalling.receptor kinases.leuc	ap13itg69687_at	Transcript	very weakly similar to (96.3) loc_os06g18000 12006.m06512 protein protein kinase domain containing protein, expressed no original description	1.9588789
30.2.11	signalling.receptor kinases.leuc	kanlowctg19800_s_at	Transcript	very weakly similar to (90.9) loc_os02g40180 12002.m09104 protein receptor-like protein kinase precursor, putative, expressed no original description	1.3234391
30.2.11	signalling.receptor kinases.leuc	ap13itg46221_s_at	Transcript	very weakly similar to (91.7) AT3G47570 | Symbols: | leucine-rich repeat transmembrane protein kinase, putative | chr3:17527611-17530748 FORWARDweakly similar to	1.4026259
30.2.11	signalling.receptor kinases.leuc	ap13itg38195_s_at	Transcript	very weakly similar to (86.7) AT3G47570 | Symbols: | leucine-rich repeat transmembrane protein kinase, putative | chr3:17527611-17530748 FORWARDweakly similar to	-1.5182016
30.2.11	signalling.receptor kinases.leuc	ap13ctg03762_at	Transcript	weakly similar to (181) loc_os02g40240 12002.m33414 protein leucine-rich repeat receptor protein kinase EXS precursor, putative, expressed no original description	1.2937868
30.2.11	signalling.receptor kinases.leuc	othswslt25539_at	Transcript	weakly similar to (101) loc_os02g40200 12002.m33773 protein receptor-like protein kinase precursor, putative, expressed no original description	1.0442657

30.2.11	signalling.receptor kinases.leuc	othswctg18100_at	Transcript	weakly similar to (131) loc_os11g36190 12011.m080025 protein receptor-like protein kinase 5 precursor, putative, expressed no original description	2.5745566
30.2.16	signalling.receptor kinases.Cath	ap13ctg04617_at	Transcript	weakly similar to (143) loc_os06g22810 12006.m06934 protein protein kinase, putative, expressed no original description	-1.5115253
30.2.17	signalling.receptor kinases.DUF	ap13itg49945_at	Transcript	very weakly similar to (85.5) AT4G03230 | Symbols: | ATP binding / kinase/ protein kinase/ protein serine/threonine kinase/ protein tyrosine kinase/ sugar binding | chr4:	1.9965222
30.2.17	signalling.receptor kinases.DUF	ap13ctg32578_s_at	Transcript	weakly similar to (101) AT4G23260 | Symbols: | ATP binding / protein kinase/ protein serine/threonine kinase/ protein tyrosine kinase | chr4:12167528-12170055 REVER	1.8421704
30.2.17	signalling.receptor kinases.DUF	ap13itg41137_at	Transcript	weakly similar to (142) loc_os09g39650 12009.m060077 protein ATP binding protein, putative, expressed no original description	1.4479766
30.2.17	signalling.receptor kinases.DUF	kanlowctg22148_s_at	Transcript	weakly similar to (142) AT4G21390 | Symbols: B120 | B120; ATP binding / protein kinase/ protein serine/threonine kinase/ sugar binding | chr4:11394458-11397474 REV	1.0061057
30.2.17	signalling.receptor kinases.DUF	ap13itg75057-rc_at	Transcript	weakly similar to (101) loc_os11g45540 12011.m08386 protein protein kinase domain containing protein, expressed no original description	1.0871619
30.2.17	signalling.receptor kinases.DUF	ap13.13172.m00006_	Transcript	weakly similar to (188) AT4G32300 | Symbols: SD2-5 | SD2-5 (S-DOMAIN-2 5); carbohydrate binding / kinase/ protein kinase | chr4:15599970-15602435 FORWARDweakl	1.8033427
30.2.17	signalling.receptor kinases.DUF	ap13ctg22601_at	Transcript	very weakly similar to (84.3) KPRO_MAIZE Putative receptor protein kinase ZmPK1 precursor (EC 2.7.11.1) - Zea mays (Maize)weakly similar to (123) loc_os03g30890 120	2.7661867
30.2.17	signalling.receptor kinases.DUF	othswctg03176_s_at	Transcript	very weakly similar to (89.4) AT1G16670 | Symbols: | protein kinase family protein | chr1:5697846-5699492 FORWARDweakly similar to (181) loc_os12g41710 12012.m	1.7296312
30.2.17	signalling.receptor kinases.DUF	kanlowctg26345_at	Transcript	weakly similar to (117) loc_os10g04730 12010.m03883 protein protein kinase, putative, expressed no original description	3.9657865
30.2.17	signalling.receptor kinases.DUF	ap13itg69009_at	Transcript	very weakly similar to (90.1) AT4G23160 | Symbols: | protein kinase family protein | chr4:12129485-12134086 FORWARDweakly similar to (166) loc_os05g39130 12005.	1.0446063
30.2.17	signalling.receptor kinases.DUF	ap13itg39900_s_at	Transcript	very weakly similar to (91.3) loc_os12g14480 12012.m05416 protein ATP binding protein, putative no original description	1.944933
30.2.17	signalling.receptor kinases.DUF	ap13itg44603_s_at	Transcript	weakly similar to (189) AT1G66920 | Symbols: | serine/threonine protein kinase, putative | chr1:24965410-24967432 REVERSEweakly similar to (111) KPRO_MAIZE Puta	1.4607781
30.2.17	signalling.receptor kinases.DUF	ap13ctg11652_at	Transcript	weakly similar to (148) loc_os05g03920 12005.m04926 protein protein kinase, putative, expressed no original description	2.3945124
30.2.17	signalling.receptor kinases.DUF	ap13itg62115-rc_at	Transcript	very weakly similar to (87.8) AT4G03230 | Symbols: | ATP binding / kinase/ protein kinase/ protein serine/threonine kinase/ protein tyrosine kinase/ sugar binding | chr4:	3.419504
30.2.17	signalling.receptor kinases.DUF	ap13itg69687_at	Transcript	very weakly similar to (96.3) loc_os06g18000 12006.m06512 protein protein kinase domain containing protein, expressed no original description	1.9588789
30.2.17	signalling.receptor kinases.DUF	ap13.13172.m00009_	Transcript	weakly similar to (132) AT5G38240 | Symbols: | serine/threonine protein kinase, putative | chr5:15277239-15279317 REVERSEvery weakly similar to (91.7) KPRO_MAIZE	1.9894791
30.2.17	signalling.receptor kinases.DUF	kanlowctg36906_at	Transcript	weakly similar to (106) AT4G32300 | Symbols: SD2-5 | SD2-5 (S-DOMAIN-2 5); carbohydrate binding / kinase/ protein kinase | chr4:15599970-15602435 FORWARDmode	1.2610515
30.2.17	signalling.receptor kinases.DUF	ap13ctg10220_s_at	Transcript	very weakly similar to (96.3) loc_os04g52840 12004.m35461 protein serine/threonine-protein kinase receptor precursor, putative, expressed no original description	1.7833976
30.2.17	signalling.receptor kinases.DUF	ap13itg53823_at	Transcript	weakly similar to (149) loc_os05g03920 12005.m04926 protein protein kinase, putative, expressed no original description	2.1282532
30.2.17	signalling.receptor kinases.DUF	alamoctg01412_at	Transcript	very weakly similar to (83.6) AT4G05200 | Symbols: | protein kinase family protein | chr4:2679793-2682309 REVERSEweakly similar to (122) loc_os11g11780 12011.m05	3.216593
30.2.17	signalling.receptor kinases.DUF	ap13ctg21940_s_at	Transcript	weakly similar to (117) AT4G23260 | Symbols: | ATP binding / protein kinase/ protein serine/threonine kinase/ protein tyrosine kinase | chr4:12167528-12170055 REVER	1.703489
30.2.17	signalling.receptor kinases.DUF	alamoctg05899_at	Transcript	weakly similar to (101) loc_os11g39490 12011.m07796 protein jacalin-like lectin domain containing protein no original description	2.349271
30.2.17	signalling.receptor kinases.DUF	ap13ctg05226_at	Transcript	very weakly similar to (87.0) POLX_TOBAC Retrovirus-related Pol polyprotein from transposon TNT 1-94 [Includes: Protease (EC 3.4.23.-); Reverse transcriptase (EC 2.7.7.4	2.6510656
30.2.17	signalling.receptor kinases.DUF	ap13itg73202_at	Transcript	weakly similar to (145) AT4G21390 | Symbols: B120 | B120; ATP binding / protein kinase/ protein serine/threonine kinase/ sugar binding | chr4:11394458-11397474 REV	3.079479
30.2.17	signalling.receptor kinases.DUF	ap13ctg48706_at	Transcript	very weakly similar to (98.6) AT4G23130 | Symbols: CRK5, RLK6 | CRK5 (CYSTEINE-RICH RLK5); kinase | chr4:12117688-12120134 REVERSEvery weakly similar to (82.8) NC	3.1529233
30.2.17	signalling.receptor kinases.DUF	ap13ctg25718_at	Transcript	weakly similar to (133) loc_os01g02770 12001.m06917 protein YRK1, putative, expressed no original description	1.6382468
30.2.17	signalling.receptor kinases.DUF	ap13ctg09428_at	Transcript	weakly similar to (114) AT4G03230 | Symbols: | ATP binding / kinase/ protein kinase/ protein serine/threonine kinase/ protein tyrosine kinase/ sugar binding | chr4:141	1.7944332
30.2.17	signalling.receptor kinases.DUF	ap13ctg22926_at	Transcript	very weakly similar to (90.5) AT4G23180 | Symbols: CRK10, RLK4 | CRK10 (CYSTEINE-RICH RLK10); ATP binding / kinase/ protein kinase/ protein serine/threonine kinase/	-1.2402747
30.2.17	signalling.receptor kinases.DUF	kanlowctg43024_s_at	Transcript	very weakly similar to (88.6) AT4G11480 | Symbols: | protein kinase family protein | chr4:6971408-6973799 FORWARDweakly similar to (167) loc_os11g11890 12011.m	-1.3382376
30.2.17	signalling.receptor kinases.DUF	kanlowctg38437_at	Transcript	very weakly similar to (96.3) AT3G45860 | Symbols: | receptor-like protein kinase, putative | chr3:16863401-16866041 REVERSEvery weakly similar to (91.3) loc_os08g25	1.8606993
30.2.20	signalling.receptor kinases.whe	ap13ctg12364_s_at	Transcript	weakly similar to (142) AT5G39030 | Symbols: | protein kinase family protein | chr5:15620066-15622486 FORWARDmoderately similar to (212) loc_os01g49614 12001.	1.8368353
30.2.20	signalling.receptor kinases.whe	ap13itg40018_s_at	Transcript	weakly similar to (134) AT5G38260 | Symbols: | serine/threonine protein kinase, putative | chr5:15283692-15285837 REVERSEweakly similar to (186) loc_os01g04480 1	1.79677
30.2.24	signalling.receptor kinases.S-lo	kanlowctg40669_s_at	Transcript	weakly similar to (121) AT4G00340 | Symbols: RLK4 | RLK4 (RECEPTOR-LIKE PROTEIN KINASE 4); protein kinase/ sugar binding | chr4:148958-151496 FORWARDweakly si	1.614365
30.2.25	signalling.receptor kinases.wall	ap13ctg19859_at	Transcript	weakly similar to (142) loc_os04g43730 12004.m09332 protein OsWAK51 - OsWAK receptor-like protein kinase, expressed no original description	1.8788317
30.2.25	signalling.receptor kinases.wall	ap13ctg14697_s_at	Transcript	weakly similar to (162) loc_os04g03830 12004.m05691 protein OsWAK29 - OsWAK receptor-like protein kinase, expressed no original description	1.4781888
30.2.25	signalling.receptor kinases.wall	ap13ctg31202_at	Transcript	very weakly similar to (99.4) AT1G79670 | Symbols: RFO1, WAKL22 | RFO1 (RESISTANCE TO FUSARIUM OXYSPORUM 1); kinase | chr1:29976887-29979337 REVERSEweak	1.1971111
30.2.25	signalling.receptor kinases.wall	ap13ctg03484_at	Transcript	weakly similar to (139) loc_os09g29584 12009.m06109 protein OsWAK84 - OsWAK receptor-like protein (OsWAK-RLP), expressed no original description	2.2482157
30.2.25	signalling.receptor kinases.wall	ap13ctg09468_at	Transcript	very weakly similar to (90.1) AT1G21240 | Symbols: WAK3 | WAK3 (wall associated kinase 3); kinase/ protein serine/threonine kinase | chr1:7434303-7436702 FORWARD	3.0043032
30.2.25	signalling.receptor kinases.wall	ap13ctg23391_at	Transcript	weakly similar to (116) loc_os03g62430 12003.m11119 protein OsWAK28 - OsWAK receptor-like protein kinase, expressed no original description	1.6576523
30.2.99	signalling.receptor kinases.misc	ap13itg41137_at	Transcript	weakly similar to (142) loc_os09g39650 12009.m060077 protein ATP binding protein, putative, expressed no original description	1.4479766
30.2.99	signalling.receptor kinases.misc	ap13ctg30066_s_at	Transcript	weakly similar to (101) loc_os03g05470 12003.m06070 protein nodulation receptor kinase precursor, putative, expressed no original description	1.4151226
30.3	signalling.calcium	ap13itg60345_at	Transcript	weakly similar to (108) AT2G18750 | Symbols: | calmodulin-binding protein | chr2:8125827-8128363 FORWARDweakly similar to (181) loc_os12g36110 12012.m07401	2.19195
30.3	signalling.calcium	ap13ctg23761_at	Transcript	weakly similar to (105) loc_os12g36110 12012.m07401 protein calmodulin binding protein, putative, expressed no original description	2.2064536
30.3	signalling.calcium	ap13ctg14744_s_at	Transcript	weakly similar to (128) loc_os12g36110 12012.m07401 protein calmodulin binding protein, putative, expressed no original description	3.343881
30.3	signalling.calcium	othswslt36625_s_at	Transcript	weakly similar to (114) AT4G23650 | Symbols: CDPK6, CPK3 | CDPK6 (CALCIUM-DEPENDENT PROTEIN KINASE 6); ATP binding / calcium ion binding / calmodulin-depende	1.9494499
30.3	signalling.calcium	ap13ctg13690_s_at	Transcript	moderately similar to (202) AT1G76040 | Symbols: CPK29 | CPK29; ATP binding / calcium ion binding / calmodulin-dependent protein kinase/ kinase/ protein kinase | ch	-1.1846402
30.3	signalling.calcium	ap13ctg15995_at	Transcript	very weakly similar to (100) AT4G35310 | Symbols: CPK5, ATCPK5 | CPK5 (calmodulin-domain protein kinase 5); ATP binding / calcium ion binding / calmodulin-depender	-1.2281529
30.3	signalling.calcium	ap13ctg08973_s_at	Transcript	very weakly similar to (81.6) AT4G37640 | Symbols: ACA2 | ACA2 (CALCIUM ATPASE 2); calcium ion transmembrane transporter/ calcium-transporting ATPase/ calmodul	1.11844
30.3	signalling.calcium	ap13ctg15995rc_at	Transcript	very weakly similar to (100) AT4G35310 | Symbols: CPK5, ATCPK5 | CPK5 (calmodulin-domain protein kinase 5); ATP binding / calcium ion binding / calmodulin-depender	-1.1966637
30.3	signalling.calcium	ap13itg72133-rc_at	Transcript	weakly similar to (135) loc_os05g05460 12005.m05073 protein calcium ion binding protein, putative, expressed no original description	-1.5819521
30.5	signalling.G-proteins	ap13ctg29685_at	Transcript	very weakly similar to (89.4) AT4G21130 | Symbols: EMB2271 | EMB2271 (EMBRYO DEFECTIVE 2271); nucleotide binding | chr4:11274308-11276286 FORWARDweakly si	1.7296008
30.5	signalling.G-proteins	kanlowctg21689_s_at	Transcript	weakly similar to (200) AT1G56050 | Symbols: | GTP-binding protein-related | chr1:20963793-20966181 FORWARDmoderately similar to (231) loc_os03g56840 12003.m	1.4021398
30.5	signalling.G-proteins	ap13ctg56018_at	Transcript	very weakly similar to (91.7) loc_os03g58540 12003.m35462 protein small GTP-binding protein domain, putative, expressed no original description	1.0517408
30.5	signalling.G-proteins	ap13ctg29685_s_at	Transcript	weakly similar to (118) AT4G21130 | Symbols: EMB2271 | EMB2271 (EMBRYO DEFECTIVE 2271); nucleotide binding | chr4:11274308-11276286 FORWARDweakly similar	2.074366
30.5	signalling.G-proteins	ap13ctg06899_at	Transcript	weakly similar to (136) AT1G48630 | Symbols: RACK1B_AT | RACK1B_AT (RECEPTOR FOR ACTIVATED C KINASE 1 B); nucleotide binding | chr1:17981977-17983268 REVE	1.5308554
30.7	signalling.14-3-3 proteins	kanlowctg01295-2_s_	Transcript	weakly similar to (107) AT1G64790 | Symbols: | binding | chr1:24065232-24081908 REVERSEweakly similar to (163) loc_os03g51140 12003.m10094 protein GCN1-like p	1.5249975
30.1	signalling.phosphorelay	ap13ctg09520_at	Transcript	very weakly similar to (100) AT3G16360 | Symbols: AHP4 | AHP4 (HPT PHOSPHOTRANSMITTER 4); histidine phosphotransfer kinase/ transferase, transferring phosphoru	-2.3115356

List of significantly DEGs involved in hormone metabolism pathway in Se/Mo comparison

Hormone

BinCode	BinName	id	type	description	Se/Mo (log2)
17.7.1.5	hormone metabolism.jasmona	kanlowctg23007_at	Transcript	weakly similar to (176) AT1G76690 | Symbols: OPR2, ATOPR2 | OPR2 | chr1:28778976-28780355 FORWARDmoderately similar to (241) loc_os06g11210 12006.m05838	5.7484293
17.7.1.5	hormone metabolism.jasmona	ap13itg71073_s_at	Transcript	weakly similar to (110) AT1G76690 | Symbols: OPR2, ATOPR2 | OPR2 | chr1:28778976-28780355 FORWARDweakly similar to (167) loc_os06g11210 12006.m05838 prot	4.396457
17.7.1.5	hormone metabolism.jasmona	ap13itg67010_s_at	Transcript	weakly similar to (115) AT1G76680 | Symbols: OPR1, ATOPR1 | OPR1; 12-oxophytodienoate reductase | chr1:28776982-28778271 FORWARDweakly similar to (128) loc_	3.1115298
17.7.1.5	hormone metabolism.jasmona	ap13itg69033_at	Transcript	weakly similar to (162) AT1G76690 | Symbols: OPR2, ATOPR2 | OPR2 | chr1:28778976-28780355 FORWARDmoderately similar to (220) loc_os06g11290 12006.m05846	2.4289663
